# Examining public views on decentralised health data sharing

**DOI:** 10.1371/journal.pone.0282257

**Published:** 2023-03-02

**Authors:** Victoria Neumann, Gail Davidge, Mike Harding, James Cunningham, Nigel Davies, Sarah Devaney, Gary Leeming, Søren Holm, John Ainsworth

**Affiliations:** 1 School of Computing and Communications, Lancaster University, Lancaster, United Kingdom; 2 Centre for Social Ethics and Policy, The University of Manchester, Manchester, United Kingdom; 3 Division of Informatics, Imaging & Data Sciences, The University of Manchester, Manchester, United Kingdom; 4 Civic Data Cooperative, Faculty of Health and Life Sciences, University of Liverpool, Liverpool, United Kingdom; 5 Centre for Medical Ethics, University of Oslo, Oslo, Norway; Jazan University Faculty of Computer Science, SAUDI ARABIA

## Abstract

In recent years, researchers have begun to explore the use of Distributed Ledger Technologies (DLT), also known as blockchain, in health data sharing contexts. However, there is a significant lack of research that examines public attitudes towards the use of this technology. In this paper, we begin to address this issue and present results from a series of focus groups which explored public views and concerns about engaging with new models of personal health data sharing in the UK. We found that participants were broadly in favour of a shift towards new decentralised models of data sharing. Retaining ‘proof’ of health information stored about patients and the capacity to provide permanent audit trails, enabled by immutable and transparent properties of DLT, were regarded as particularly valuable for our participants and prospective data custodians. Participants also identified other potential benefits such as supporting people to become more health data literate and enabling patients to make informed decisions about how their data was shared and with whom. However, participants also voiced concerns about the potential to further exacerbate existing health and digital inequalities. Participants were also apprehensive about the removal of intermediaries in the design of personal health informatics systems.

## Introduction

Traditional patient health data management services have followed a highly centralised model with public and private health providers acting as data custodians on behalf of patients and as intermediaries for third-party data consumers such as pharmaceutical organisations. More recently, researchers have begun to explore the possibilities of using Distributed Ledger Technologies (DLT), also known as blockchain as an alternative, decentralised form of personal health data management, which can support individuals to have more direct control over their health data. The capacity of DLTs to facilitate more secure data sharing and offer verification between disparate healthcare information systems such as personal electronic health records (EHRs) and other digital personal health monitoring technologies offer additional benefits when compared to more traditional, centralised models [1, 2].

Current research has predominantly focused on developing proof-of-concept solutions to interoperability challenges between different healthcare management systems and resolving issues regarding incompatibility with data protection legislation, originally designed for centralised models [3]. It remains imperative that research and innovation continue to work towards addressing some of the challenges that surround integrating independent healthcare systems [4, 5]. However, research in this field is often led by the agenda of technology enthusiasts rather than being driven by the views and needs of people that have a personal stake in sharing or using health data. Therefore, there is a further need to recognise the role of patients as data subjects and to examine public perceptions of the social and ethical ramifications of introducing DLT within health data sharing eco-systems [6, 7]. This paper aims to address the gap of user-oriented design research in this field, report on the initial phase of a longitudinal co-design process that aims to create a technical platform to enable (i) patients within the UK to better manage access to personal health data and (ii) provide clinicians and third-party consumers with more seamless mechanisms for health data acquisition.

In contrast to previous work [8–10], we aim to prioritise and account for a broad range of end-user needs and concerns. We therefore report on the findings of a series of user engagements which contribute to emerging knowledge on the design of future personal health data sharing mechanisms [11]. This piece of work explicitly aimed to identify user requirements as an initial assessment of laypersons attitudes to DLT features and how they contextualized them within their experiences of interacting with technology-enabled healthcare. These insights can be used to advance user requirements and develop nuanced applications that are not only technically sound, but also socially desirable. Following an interdisciplinary, user-centred design approach, our research explores public perceptions of transitioning towards more decentralised models of health data management through an extensive, qualitative analysis of group discussions involving 36 participants. In particular, in this paper we report on:

1. Public attitudes towards fundamental capabilities (i.e. immutability, decentralisation, automation) of DLTs in healthcare; and
2. Insights into methodological approaches that seek to engage lay audiences into debates about emerging DLT technologies.

### Related work

Sharing personal health data from clinical records, wearable fitness and location-tracking devices enables clinical researchers to deliver more timely and effective medical interventions to address significant health challenges in society [12]. However, the consequences of high-profile cyber-attacks on care institutions and the subsequent implications for patient data privacy [13] have led researchers to consider alternative data exchange models for better securing and protecting patients’ health data, including Internet of Things (IoT) approaches [14, 15] and DLTs [16, 17].

DLTs offer a decentralized peer-to-peer database architecture, consisting of a network of participants referred to as ‘nodes’. Each node in the distributed network stores an identical copy of the entire blockchain and contributes to the collective process of validating digital transactions for the network. Consensus algorithms, such as proof-of stake (PoS) or proof-of-work (PoW), are used to reach an agreement to add a block of transactions to the chain. This combination of distributed, agreed upon copies, together with the use of consensus algorithms largely prevent the unauthorized modification of data. This enhances the appeal of utilising blockchain in contexts whereby a ‘tamper-proof’ option for securing sensitive data is vital to maintaining data integrity. As the DLT provides both a transparent and immutable ledger of all transactions to all nodes, this holds significant appeal in terms of auditing capabilities [18]. In addition, DLTs use a range of privacy-preserving security and cryptographic protocols such as public/private key pairs, zero-knowledge proofs and pseudonymity, to ensure that all data is encrypted and only authorised parties have access. Within healthcare, DLT proposals often store sensitive data off-chain for additional protection [2, 18]. Further, some blockchains can manage data access agreements via Smart Contracts. These are computer programs which are only executed when certain conditions are met. This type of automated ‘contract’ has drawn particular interest as a way of enabling greater patient control over the integration of granular consent protocols and the execution of the consent choices [1, 2].

#### DLT applications in healthcare

Extensive investigation has been conducted to assess the technical feasibility of leveraging novel applications of DLT technologies in healthcare [10, 16, 19–21]. Several studies have explored the use of smart contracts, zero-knowledge proofs [5, 17, 22–25], and non-fungible tokens (NFTs) [26] to provide patients with new data-sharing capabilities that prioritise user privacy, data integrity and security. Moreover, several studies [23, 27] have investigated the use of smart contracts to streamline the sharing of patient healthcare records. An awareness of these emerging domain applications and assessments of blockchain in a healthcare setting served to inform the development of several narrative scenarios (as outlined in the Materials and methods section) to introduce DLT concepts during public debates. However, this paper does not attempt to assess the technical feasibility of a particular blockchain-based solution as this has been investigated within other phases of our research [23, 26]. The focus of this paper outlines our research on public views and expectations. We therefore (i) acknowledge the importance of patients, as health data subjects, whom such systems are ultimately being designed to serve, and (ii) aim to augment existing technical DLT design research with a grounding into the social concerns and public perspectives in the potential application of decentralised mechanisms (e.g. self-sovereignty over health data [28]) in the management of health-related information.

#### Stakeholder perspectives on DLT in healthcare

A limited number of studies have been undertaken to examine a range of stakeholder perspectives on the use of DLTs in the health sector. For example, Yeung [29] assessed the likelihood of blockchain’s theoretical potential (e.g. security & privacy protections) in the transformation of healthcare by reviewing state-of-the-art applications of DLT. In contrast to our focus on public concerns, Yeung [29] engaged with care professionals and blockchain experts to derive implementation challenges of blockchain-based applications based on views from healthcare organisations. Similarly, Hau et al. [9] surveyed healthcare professional and patient attitudes towards the use of DLTs in managing medical information and found care professionals demonstrated greater negativity towards the technology than patients.

Through focus groups with the public, Lu et al [8] identified early insights into the intentions of care consumers towards the adoption of a DLT-based health record system that provided individuals autonomy over their personal health information for sharing purposes. In particular, the study provided a stark characterisation of patient attitudes which suggested a limited appetite for such a system due to a number of similar concerns our participants discussed (e.g. irrevocability of data on a blockchain). However, our participants appeared to go further in highlighting broader social, societal and ethical dilemmas regarding the use of DLTs in managing personal health data, such as concerns over the digital literacies of healthcare professionals (HCPs) and the ability of traditional health regulatory frameworks to protect patient data (see Results section for further details).

Lemieux et al.’s [30] work is representative of an early attempt to involve the public in the co-creation of a blockchain-based technical artefact that aimed to seed follow-on discussions exploring attitudes towards the use of DLTs to “manage, control and share” [30] personal health data. Our work aligns with several findings that emerged from this work, such as a perceived lack of understanding of how the technology worked, resulting in public concerns over their ability to trust and accept it in a health context.

## Materials and methods

In order to engage with patients and elicit their attitudes towards DLT capabilities within a healthcare context, our methodological framework drew upon ‘upstream’ models of public engagement [31, 32]. This type of approach is often used for engaging a lay audience with unfamiliar, emerging technologies, such as discussion methods derived from clinical research [33] and contextualising properties of technology within more familiar terms of reference [34]. Upstream engagement takes place in areas of emerging technologies, which have not fully developed yet or where no significant public discourse has taken place. This is also true for the application of DLT in healthcare. While there are similarities to traditional risk communication, however, upstream engagement aims for values and future visions as Pidgeon & Rogers-Hayden [35] note (p. 205): “[…] ‘upstream’ public engagement on emerging health technologies like nanotechnologies, to be successful, must move beyond conventional ‘risk communication’ based dialogue, to be future focused, broadly framed, and to explicitly incorporate questions of both public values and technology governance.” This extraction of underlying values, mental models and public understandings is particularly useful for design research in Human-Computer-Interaction and to elicit user requirements for future developments of technologies.

In order to frame participants’ understandings and conceptualisation of the potential use of DLTs, the project team developed a series of narrative scenarios. Narrative scenarios are stories commonly used for prototyping and speculative design in Human-Computer-Interaction as well as science communication [36] to increase engagement and foster comprehension of non-expert audiences [37]. This engagement allows for the end users of such technologies to add their view points. These insights can subsequently be used to further the design elements of such technologies to fit user requirements. Our scenarios characterised more familiar user interactions with key features of a DLT-supported data sharing technology. For example, unique features of a DLT based data donation platform were introduced through a narrative about a patient deciding to share their own health data with researchers of a rare disease. This was a deliberate strategy to steer the discussion towards debate around how blockchain might be used by the public in healthcare contexts, rather than directing the focus towards educating the public about the intricacies of the technology itself. In order to enrich participant discussions and broaden the debate, we included a range of alternative stakeholder viewpoints and data sharing contexts to help the public to imagine a wide range of perspectives [38]. This enabled our approach to be as inclusive as possible and create an imaginary space in which public stakeholders were able to think through the possibilities of blockchain-based health data sharing applications for themselves and others, identify points of concern, and relate potential use-cases to their own everyday lives, needs and future requirements without having to have prior technical expertise and knowledge of DLT. Our approach followed a structured, and iterative process detailed in the following sections.

First, we developed a set of narrative scenarios that reflected how blockchain technology could be used by a wide range of public and professional actors in healthcare data eco-systems. Second, we selected scenarios that illustrated everyday health data sharing contexts such as patients engaging with medical research or sharing health monitoring device information or application data with healthcare professionals (HCPs). Third, we tested a draft of our focus group resource materials, timing and framing with a pilot focus group. Finally, building upon feedback from the pilot group, we further refined the focus group presentations and scenario resources for subsequent focus groups.

### Scenario development

Public engagement materials were designed and refined over a period of five months as a result of reviewing related literature, iterative work and collaboration between a multi-disciplinary research team. This included members with expertise in the fields of computer science, health informatics, ethics, law, psychology and social sciences. Final drafts of the scenarios were also edited following feedback from an international advisory board. The resulting series of narrative use-case scenarios were located in the wider area of blockchain and healthcare data management and included material drawn from related work into recent research trends and use cases (For an overview please visit the CDIP project website: https://cdip.lancs.ac.uk/). We decided to focus on three scenarios that placed fictionalized individual persons as the central protagonist of each story and imagined contexts in which an individual would make decisions about sharing health data with a range of different stakeholders and organisations such as primary care physicians (in the UK also called General Practitioners), other healthcare professionals, health researchers, charities and businesses via a DLT supported infrastructure (for an overview see Table 1).

**Table 1:** CDIP public engagement scenarios overview.

Application	Features Highlighted	Related DLT concepts
Health App Data Exchange	Privacy-preserving security; Tamper-proof records; Automated & Granular Consent	Secure Data Access
Data Donation Platform	Automated & granular consent; Permanent & Immutable records; Transparent data transactions	Smart Contracts
Health Passport	Privacy-preserving security; Tamper-proof records; Decentralisation	Cryptographic features

One scenario featured a secure data exchange where patients could track and share health data via medical devices or health apps with healthcare professionals. Another narrative and featured a health passport offering an authoritative, tamper-proof record of a person’s health or immunisation status [39]. This potential use case was framed around a topical context relating to the Sars-CoV-2-pandemic and highlighted how tamper-proof properties afforded opportunities to gain access to social or business settings, in Sars-CoV-2-related contexts without directly sharing personal, identifying information. The final scenario highlighted a data donation platform that enabled citizens to share medical data with research organisations such as pharmaceutical companies or universities.

**Fig 1:**
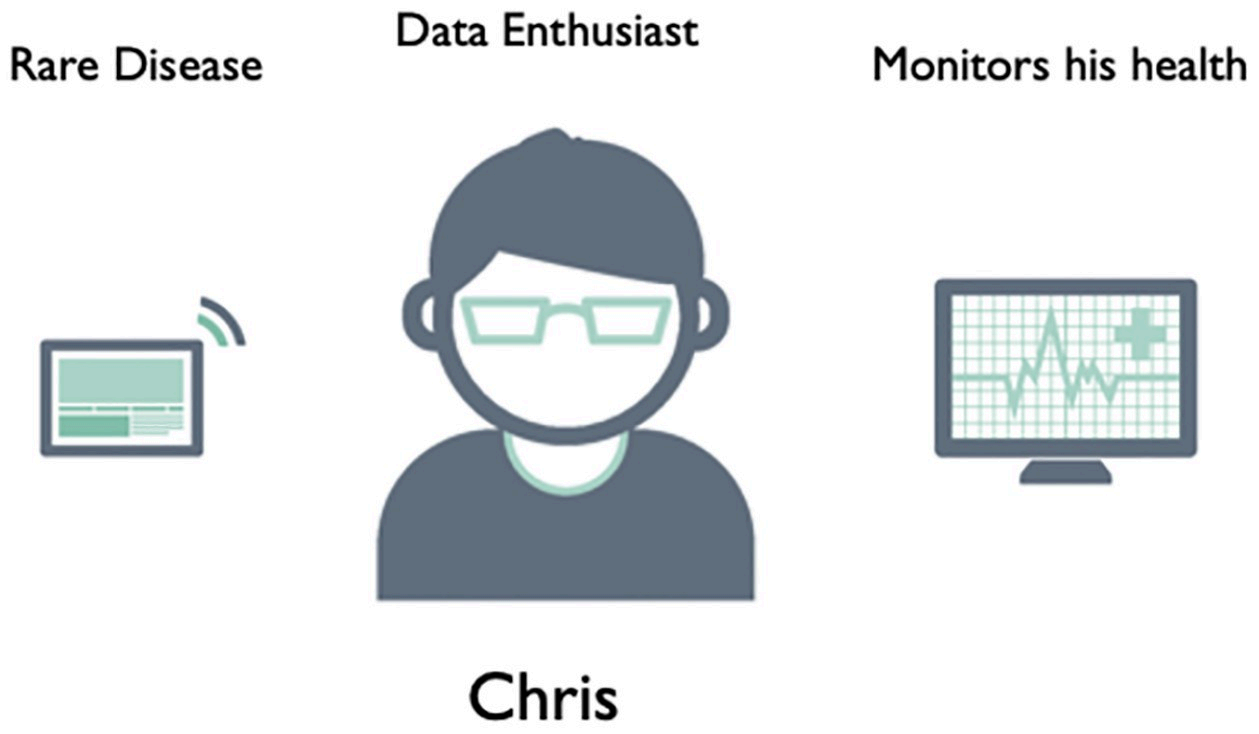
This slide was presented to a subset of participants during the focus group to introduce the data donation scenario with a fictionalised character with the following script: “Chris has a rare liver disease. He is also a data enthusiast and uses various devices to track and monitor his movements, behaviour and bodily functions to stay as healthy as he can. He is keen to help use his medical data to try and advance treatments for other people who share the same condition. Despite undertaking a lot of online research, he doubts that pharmaceutical companies will invest in finding a cure for his condition because it only affects a tiny proportion of the population.”

All scenarios presented to the focus groups participants were structured in the following way: The first slide introduced a fictional character within an everyday context of sharing healthcare data (see Fig 1).

**Fig 2:**
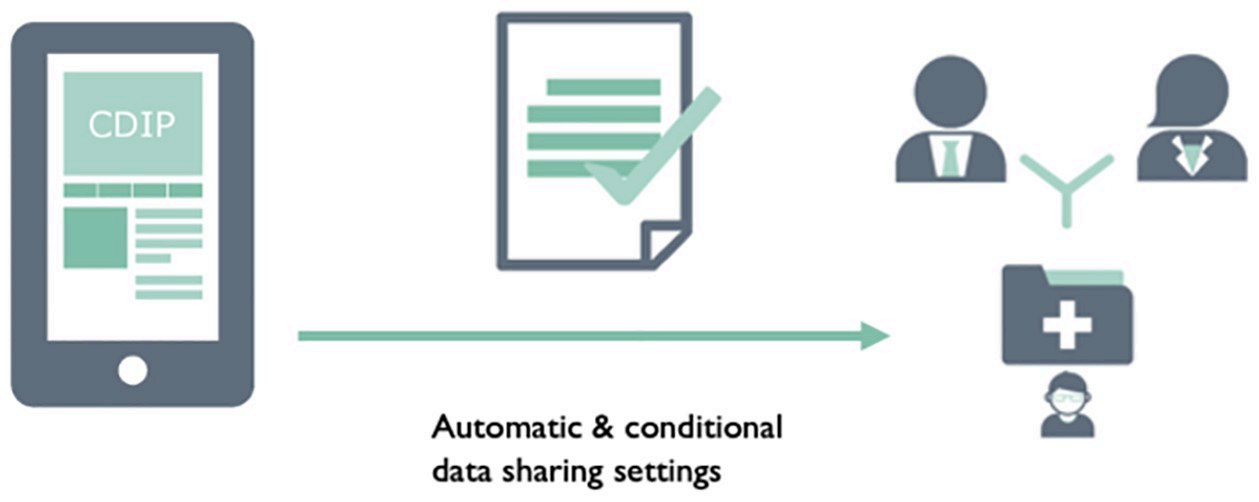
This slide was presented to a subset of participants during the focus group to highlight the blockchain features of the data donation scenario. There were three feature slides for each scenario. This data donation blockchain feature slide was presented to participants with the following script: “Chris decides to download the CDIP data donation app on his smartphone and registers his interest in sharing his health data as well as taking part in health research and clinical trials. He considers which health data he would like to donate and then sets up a smart contract that automatically approves his consent to share anonymized data from his electronic health record with the British Liver Research Trust.”

**Fig 3:**
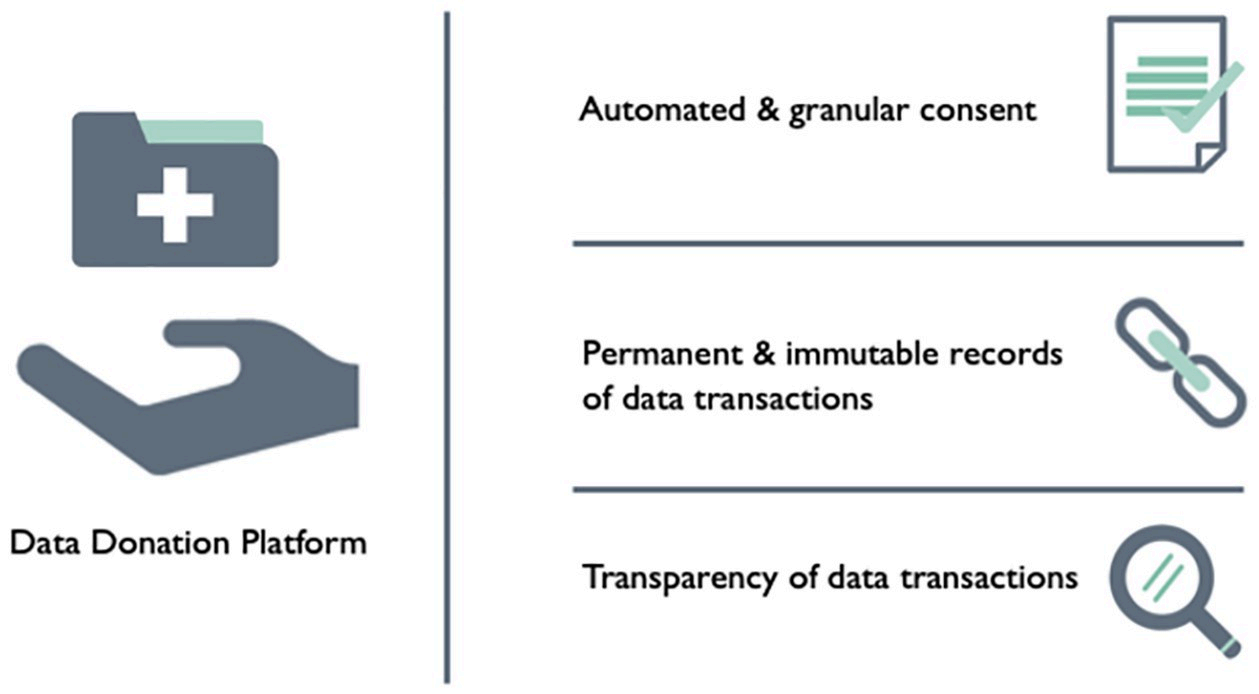
Data donation scenario blockchain feature summary slide. This slide was presented to a subset of participants during the focus group to summarise the blockchain features of the data donation scenario with the following script: “1. Automation & granular consent: Patient information exchanged via the CDIP platform can be de-identified and permission to access this can be granted through smart contracts. This consent is a digitally encoded agreement between two people in the form of computer code which will only be executed if a number of conditions are met. For example, Chris can decide and say that his GP can access his Fitbit data, but pharmaceutical companies cannot. For example, a smart contract might be written to automatically enable healthcare professionals to access a patient’s medical record only if certain conditions are met: (1) that the patient has consented Healthcare Professionals (HCP) access and (2) the HCP can prove authority to access confidential patient files. 2. Immutability: Transactions on the blockchain are permanently recorded. For example, this makes it almost impossible for a user to alter details of a person’s medical history or the results of a clinical trial. 3. Transparency: Personal data will NOT be stored directly on the CDIP platform but ‘pointers’ of all data transactions will allow any users to be able to trace the flow of data between different entities. Transactional data stored on the blockchain will be visible to all approved participants. You can see/verify who is accessing your health data at every stage, for example if your doctors have accessed or viewed your data.”

This was, followed by three slides that highlighted features of a potential blockchain-based application to encounter this issue (see Fig 2). Finally, we summarised the DLT features which were utilised in each application use-case (For example, see Fig 3).

The highlighted features (as seen in Table 1) in each scenario allowed for reflection of DLT technologies within a common healthcare setting familiar to the participants without the need for a deep understanding of the underlying technical complexities. Further, DLT features highlighted in the scenarios were present in at least two scenarios, allowing us to make more generalized claims on public perceptions on such features.

### Online focus group and format

During September and December 2020, the research team facilitated five online focus group discussions which explored public perceptions on emergent features of blockchain in healthcare with members of the general public located in England. Thirty participants were recruited from a range of health research charities and patient and public involvement groups. In addition, six PhD students also took part in earlier discussions as part of a pilot focus group which was used to test out the draft scenario resources. Project participants were provided with an information sheet about the study and the project team obtained written, informed consent to take part in the project prior to engaging in focus group discussions. Participants received a shopping voucher as a thank you for taking part. Ethical approval was sought from The University of Manchester Proportionate Research Ethics Committee (Ref: 2020–9648-16110).

Discussion groups lasted between ninety minutes and two hours. Each focus group included discussion based on at least two of the aforementioned scenarios which were rotated between discussion groups in order to ensure equal exposure. The focus groups were held via the video conferencing tool Zoom as this phase of fieldwork took place during the pandemic with social distancing policies in place. Discussion groups were capped at a maximum of six participants in order to maximise opportunities for all participants to express their views. Three researchers were involved in the facilitation of the focus groups in order that a researcher was readily available to address any technical issues at any point during the discussions. Participants were also encouraged to use the chat facility during focus group discussions to comment or raise concerns whilst another participant was speaking or if they felt unable or uncomfortable with verbal interaction. Chat comments were moderated by a researcher and any points raised via this facility were introduced to verbal discussions throughout the duration of the focus group. The pilot was conducted to test if engagement materials, timing and format of the focus group designs were appropriate and accessible to a wider public audience. The pilot revealed that, despite considerable effort to produce easy-to-understand information (short animation, beginner level technological introduction into basic concepts) about DLTs, participants spent the majority of time questioning and trying to understand how blockchain technology worked, rather than discussing design requirements for the CDIP platform and scenario use-cases. Consequently, we omitted the animation and reformatted the materials so that they highlighted the features of DLTs (such as transparency, immutability or decentralisation) rather than attempting to articulate a beginner’s guide to understanding blockchain technology more widely. We also introduced other stakeholder perspectives such as lawyers, researchers, laypeople as end-users) to trigger a deeper exploration of the topics based on the previous work [38].

The restructuring of the focus group format and presentation proved to be much more successful in generating useful points for further deliberation and debate between focus group participants. The final format utilised within focus group discussion sessions included an overview of the project aims and objectives, a short description of the current health data sharing context and presentation of two use case scenarios.

After the presentation of each user story scenario, we asked participants the following questions:

1. What are the major benefits and drawbacks of this use case example?
2. What choices and controls would you like to have over your own health data?
3. What information is important for you to know before using a platform like this?

Focus group discussions were recorded on Zoom and transcribed by an independent transcription service. Members of the research team undertook an in-depth thematic analysis of the data following a grounded theory approach [40] using NVivo software [41]. This analysis was discussed in a full team analysis half-day workshop and subsequent meetings. The next section highlights an overview of participant’s responses to the narrative scenarios and the resulting concerns and design requirements that have been articulated thus far in the platform co-design process.

## Results

The views conveyed by focus group participants were complex and potential DLT solutions were often considered in relation to the current conditions in healthcare ecologies. Participants were particularly enthusiastic about enhanced transparency and valued the ability to monitor access to their health data as well as being able to independently authenticate their health or immunisation status to other third parties situated outside of the NHS context. However, there were concerns about the feasibility of utilising blockchain technology within the current UK, public-health National Health Service (NHS) context. For example, many questions arose in all focus groups concerning how DLTs may be able to integrate into healthcare organisations with already poorly-functioning IT systems and significant interoperability problems. Participants also raised concerns about levels of digital literacy amongst HCPs or worried that this type of system might over-burden primary care physicians and exacerbate existing heavy work-loads.

### Immutability, transparency and tamper-proof recording

Immutability was a controversial topic. On the one hand, some participants were confused about this feature and considered that it may potentially jeopardise their right to be forgotten and infringe respective data deletion options granted by the EU-wide General Data Protection Regulation (GDPR). Other participants were concerned that if they granted consent via a blockchain platform that this would mean that they could no longer utilise their right to withdraw from existing research trials and projects as part of the existing informed consent process: “In a lot of research, the participants get the option, up to a certain point, of withdrawing their data for whatever reason, and I’m just wondering why does this have to be permanent? Why can’t people withdraw their data if they want to?” (P40). Whilst the feature of immutability was perceived as a significant benefit in terms of auditing and monitoring historic access to their health data, immutability was also seen as a potential obstacle due to concerns regarding incorrect information being written within their health record. This was connected to a strong desire to be able to interact with their own health record, such as editing or amending data, to which the idea of an uneditable record was viewed as an obstacle to participation. Nevertheless, participants also had possible suggestions to lever potential negative impacts such as pop-ups that flag up a later-added amendment or correction.

The scenarios presented to participants highlighted features that only enabled the permanent recording of permissions and transactions ‘on-chain’ and underlined the fact that health data would continue to be stored ‘off-chain’. Nonetheless, misunderstandings and anxieties about immutability came up repeatedly, needing clarification in almost all focus groups. In addition, despite this repeated clarification about health data being stored outside of the blockchain, participants wanted further reassurances that a transaction record of consenting to engage with a particular trial would not make them identifiable or impact them negatively in other ways, such as preventing participation in future trials. In contrast, other participants saw immutability as an ‘honesty’ feature:

“I personally wouldn’t be bothered. I don’t really see any downside to that, unless it stops me getting another trial on the back of it, but then that shouldn’t be the case, because otherwise you’re lying, so I really don’t see an issue with that” (P34).

Enhanced transparency was highlighted as a benefit that would incentive people to use DLT platforms, regardless of use case. Participants were particularly enthusiastic about the promised transparency of DLTs as this could also serve as a mechanism for monitoring data access in the form of an immutable audit trail. They saw the benefit in an accessible history or ledger of their own health data: “…the idea of the audit trail is fabulous, so you can see who’s looked at my medical records, it’s one of the main, I think, positives” (P37). A small subset of participants also saw immutability as a beneficial tool for professionals working in information governance. Some participants drew attention to the fact that existing centralised systems do not afford comparable levels of transparency regarding who has accessed their medical data at present: “…people actually have access to what data they want to share and they actually know what specific organisations …what sorts of information they take from them. Because I don’t necessarily know of any other way, at the minute, that you can do that” (P11).

Participants linked having a more transparent, accessible data record with other benefits such as added convenience or reducing bureaucracy and administration costs. they highlighted examples such as being able to authenticate test results and vaccinations, or sharing bills and proof of treatments with private insurance companies (which are operating in addition to taxpayer-funded, non-profit NHS in the UK), occupational health, or immigration officers. Greater transparency of individuals’ access to records that show which data have been used, when and by whom, was also seen as a means to increase data literacy and control over data privacy in the general population: “So I think this technology could have a potentially other function of making people more data literate in that they have to think about it more when they are deciding how it’s used, which is what I think is a good thing about it. It’s great” (P04).

### Privacy-preserving security

Privacy-preserving features and minimal data sharing had considerable overlap with discussions around granular consent choices and balancing high levels of choice and control with the need to only share necessary data. One participant drew attention to potential problems around data-sharing literacy and other challenges in terms of adhering to data minimisation principles contained within GDPR guidelines. For example, one participant questioned: “…who is the decision maker about what is absolutely necessary in this kind of transaction [*deciding which data to share*]? …That might be quite burdensome on the individual, not just in terms of time, but in terms of understanding as well. You know, the necessity for that data being limited” (P03). Some participants saw anonymity as a high priority and the possibility of sharing relevant data without revealing an individual’s identity was attractive. However, participants also demonstrated awareness of the limits to promises of anonymity such as re-identification through secondary data access, via criminal or illegitimate activities, or by the presence of statistical outliers: “In relation to the kind of rare disease aspect, in the vast majority of circumstances, no matter how much you anonymise it, it’s identifiable. If you are one of ten people in the UK with a particular disease or illness, your data is going to be identifiable” (P03).

Other discussions focused on the risks of potential abuse of power and social exclusion that neither anonymity nor privacy-preserving technology could prevent. In particular, the idea that health data sharing becomes a prerequisite to accessing certain services in the case of proving one’s health status, for example, was seen as perpetuating existing inequalities that could lead to discrimination: “The other concern is, of course, there’s older people that won’t have this technology. So you’re limiting it almost by definition, you’re limiting it to younger people. And that’s a major concern to me” (P29).

In addition, within many discussions, the risk of exacerbating existing problems connected to digital poverty was evident when the discussion around digital divides and health data literacy emerged alongside the consent themes, as one participant puts it: “And it’s the equity I think, you know, people without smartphones, it’s not equitable. People with a learning disability, you know” (P28). Moreover, whilst it was acknowledged that the use of blockchain technology may support the greater empowerment of some citizens, participants were keen to know what else could be done to try and make this type of application more inclusive and accessible.

### Automated and granular consent

Discussion in focus groups around smart contracts echoes the current research around informed consent in healthcare [42]. On the one hand, participants indicated that they would like to increase their individual choice and have a maximum of granularity to set data sharing preferences. On the other hand, they were aware of the limitations of individual consent such as overburden, obstacles to be informed all the time, social pressures, or mental capacity issues. Despite this, they remained curious about the automated execution of consent preferences. Participants connected automation with the possibility of making their preferences interoperable across different healthcare services. It was also connected to streamlining consent processes, which would make it easier for example to join studies or trials: “I think it’s a great idea. I can imagine lots of different situations where it’s easier to invite people to participate…I can imagine that this would make it a lot more seamless for those that choose to opt-in or opt-out” (P04). Additionally, streamlining was also seen as time saving, especially for people who share data on regular basis: “I think the positive thing that I’ve noticed is, like, just to share some information. I’m someone who has a rare disability… I’ve given a lot of data and done trials, and the amount of consent that you have to do is quite onerous really” (P02).

### Decentralisation, regulation & responsibility

Decentralisation was one of the most contested discussion points. Some participants saw benefits in decentralisation when it was combined with localised spending or health interventions. However, most participants connected decentralisation with de-regulation, and ambiguity around responsibility and accountability. Overall, participants favoured a model where oversight was included: “I think this is too serious an issue to be left alone, it has to be monitored and regulated” (P30). Another participant noted that a completely decentralized, public model might be, “wide open to abuse” (P34). There was also confusion around what decentralisation means for the different actors using this technology and many questions and concerns centred around clarifying the ‘ownership’ of the platform. Lack of a central authority was also linked to the question of who might undertake roles around the verification and gatekeeping for data users:

I think you’re going to have to have some kind of governance system or process that actually monitors companies. There needs to be a formal process through which companies, in my opinion, would have to go before you would want them on the platform, and that is a big concern, especially with so many fake companies, companies moving around, going into liquidation and so on. So there needs to be a formal process. (P39)

The distribution of responsibility for regulatory oversight was generally viewed through the lens of existing systems, reliant upon external modes of authority, with no clear preference of who or what might undertake this regulatory function within a DLT based, emerging context. Potential candidates ranged from independent bodies, to government oversight, to public health services. Despite this, however, there was also some recognition of the capacity of this type of technology to offer a more, collective community approach: “…like, crowdfunding effort, crowd-led ethics, … rather than having an independent committee that might be beholden to other people” (P10).

## Discussion

Overall, our early engagements with the public highlighted a number of social dilemmas regarding the use of DLTs in personal health data sharing that characterise the interrelatedness between leveraging the technical properties of DLTs (i.e. immutability, decentralisation and automation) and important social considerations such as, equity, digital literacy, regulation and moral responsibilities. Introducing DLT through narrative scenarios created a space within which the public was more able to re-imagine the relationships between their role as health data subjects and data consumers, and articulate a vision of distributed responsibility. For example, focus group discussions brought into view the ways that participants began to make sense of the technical aspects of DLT by debating the wider social impacts and questioning the potential effects of decentralised data management upon the roles and responsibilities of different stakeholders and actors within healthcare ecologies.

Immutability and transparency properties were seen as useful features for individuals, and to a certain extent for data custodians, via their potential to provide permanent audit trails. Many participants were in favour of being able to audit and retain ‘proof’ of health information stored about them. Participants even highlighted other potential benefits such as supporting people to become more literate about their health data and make informed decisions about how it was shared and with whom. In relation to this, participants were exposed to new possibilities of interaction with data, a capability which they believed current centralised systems do not provide. However, whilst we explicitly emphasised that personal health data would not be held ‘on-chain’, the idea of permanency and inability to edit, change, or remove data remained a significant concern to participants, despite our attempts to reassure. This highlights a need for designers to consider how such systems communicate how data is being stored and underlines the utility of providing potential users with educational tools that can simply convey how user data is stored and handled.

Participants’ views on greater granularity and automation around consent and data access choices were not always regarded as a positive feature, but an essential one to exercise autonomy. At the same time, they expressed concerns that being given greater control and extension of choices regarding consent options might also result in an increased burden and possible exploitation of vulnerable populations. In order to navigate this dilemma, participants indicated preferences around maximising individual choices, with the caveat that extra safeguarding, education and regulatory oversight are made available to support all patients, especially more vulnerable populations, in the management and understanding of the consequences of different consent decisions.

Broadly speaking, participants regarded decentralisation as a feature of DLT that afforded them more active participation, with the potential to re-frame their role as a more proactive patient or healthcare consumer. For example, participants identified benefits that included improvement in health outcomes as a result of sharing data from devices and apps with healthcare professionals, as well as, added convenience and control over the administrative aspects of sharing their health data status with different organisations. However, the concept of decentralisation did not sit comfortably with many participants in terms of potential impacts upon governance and regulatory issues. Participant discussion indicated that understandings of different stakeholder’s roles concerning the distribution of responsibility and accountability within health data sharing domains appeared to be grounded in more traditional schemas. For example conceptualising responsibility for regulation by drawing upon prior knowledge of archetypal, centralised models. Questions and debates around which actors or organisations were ultimately responsible for the regulation and governance of the system highlighted concerns and further questions about moral responsibilities over the handling and processing of such sensitive data. Participants also underscored an almost unanimous desire for a visible, named actor upon which they could consult or rely upon to help ‘if something went wrong’. They regarded the role of an intermediary as necessary to ensure that possibilities for abuse are minimised. For example, ensuring that robust checks and verifications are undertaken to establish data consumer integrity. This indicates that there remains a significant amount of work to be done in order to understand how to generate enough trust in the properties of DLT to persuade members of the public to adopt alternative models of decentralised data management on a wider scale. In addition, these findings also highlight opportunities to explore which models of governance and ownership have the most potential to garner sufficient public trust and appeal.

The concerns highlighted by our participants echo existing debates within legal literature around DLT application with regards to legal frameworks such as GDPR [3, 43, 44]. Legal scholars conclude that there is a clash between frameworks that are modelled on centralised data storage models and therefore do not address issues arising from decentralised modes of data transfers [3]. Accountability and local jurisdictions for decentralised organisations remain a problem that are yet to be addressed on a greater scale. For example, parallels can be drawn to legislation introduced in Wyoming where blockchain enabled, decentralized networks still have to register with a named contact to resolve liability and agency issues [45, 46].

To conclude, the successful deployment of DLT platforms in healthcare data sharing ecosystems requires the cooperation of multiple actors, institutions and stakeholders. Acceptability amongst healthcare consumers is not only dependent on the technology itself but also the organisational structures around it. These transformations bring challenges for the design choices and future applications of health data sharing initiatives based on blockchain technology. Analysis of participant discussions has revealed that in order to be socially and ethically desirable, DLT technology needs to extend public and user participation within healthcare systems, whilst also ensuring sufficient protection via technical architecture, more granular controls and effective institutional oversight. These evaluations broaden the current debate which has thus far exclusively centred on claims made by researchers and developers, rather than end-users.

## Conclusion

Public perceptions of blockchain are highly contextualised. Our research set out to generate a ‘bottom-up’ approach to understanding what the public desire along with the identification of related concerns towards new models of health data sharing. Participants highlighted a number of important requirements and concerns that demand further exploration in the next phase of the co-design process. Further work is now required in order to generate more in-depth discussions around alternative models of governance and regulation within DLT models, as well as generating further understanding about the importance of trust and reputation in a business model that will be socially desirable. In phase two of our research, we will re-engage with the same cohort of participants and present technical probes to explore the viability of a range of different business model implementations including a fully decentralised, public blockchain, a hybrid and a private model of a DLT based data sharing platform. These probes will also include work that explores how the public can engage with different forms of incentivisation for data sharing across a range of transactions with various data users.

## Supporting information

S1 Appendix Anonymized transcripts. Appendix has been submitted with this paper and contains anonymized transcripts from all six focus groups (including the pilot).
(PDF)
