# Supplementary material for: Examining public views on decentralised health data sharing
Source: PLoS One. 2023 Mar 2;18(3):e0282257. doi: 10.1371/journal.pone.0282257 (PMC9980819; doi:10.1371/journal.pone.0282257)
Supplement: S1 Appendix — Appendix has been submitted with this paper and contains anonymized transcripts from all six focus groups (including the pilot). (PDF) [file pone.0282257.s001.pdf]

**File Name(s)**	55407_FG1 CDIP-pilot-focus-group
**Total Recording Length**	46 minutes
**Number of Participants**	9 (P01-P06) Researchers GD, MH & VN
**Final Word Count**	6,711
**Total Number of Pages**	15
**Date Completed**	25 September 2020

GD: How would it be if we just go…first, initially, just go through each person and ask you for your initial views? So P05, you're top of my list at the moment. Have you got anything that you'd like to add?

P05: Yeah, I guess it's more of a clarification question though perhaps on my…I'm not quite getting the blockchain thing. It's just it seems very centralised as an app. If the focus is on decentralising data and things, it seems almost like a portal for people to log on and actually put their information in than it is centralising it for them. So I'm just kind of trying to get my head around the bit that's almost the decentralised element of that. 'Cause I kind of thought with blockchain it's almost like open to everyone, that you can actually add blocks into the chain as it were and there's that transparency. So yeah, just trying to sort of pull it apart in my head to spot how this isn't centralised.

GD: Okay. I'm just…

VN: I think there's two differences here. So one is, of course, if you have an app on your smartphone, it looks centralised to you as you using it. But the technology behind it depends a bit on how we set it up. So if you set it up in a way as major cryptocurrencies do, then everyone can sign up for themselves and everyone has the same kind of roles and abilities to actually participate in them. What you could also do is make like a commissioned or hybrid form where you have somebody who has to approve you, which is like semi-centralised but it's not really. Because once you're approved, you kind of have still this ability to add blocks or to verify them. Did that clarify it a little bit?

M: Yeah, can I just add to this as well? So obviously, typically with these types of system, you know, you can imagine that the NHS may be the sole kind of management authority over the datasets. So they might control the app, they might control the database, and obviously with a blockchain implementation, you could potentially have multiple different institutions that might validate the data that's written to that database.

So kind of one of the key features that you get with a DLT or a blockchain is this aspect of disintermediation, so instead of just relying on the NHS to manage your data, there may be other trusted third-party entities. So it could be another government agency or somebody else that may also have an ability to validate the data that's written to that database.

P05: Okay, so who would be those institutions? Would that be decided by people that are creating this platform? Or would I as a user say, actually I'm not happy with that agency being one of the people that can see and validate data because I just don't trust that particular government agency or whatever?

GD: That's a great question, P05. This is why we're doing the focus groups really, is to find out what people want to be able to do, how much control people want to have, whether people don't want to be blinded by questions

and want to be able to just say, yes I'm happy to share my data with anybody, or if they want to make caveats as to who can access that data. This is what we're trying to explore now, the kinds of organisations that you'll be happy to share data with, or whether people would want some kind of trusted intermediary like an information governance office or a separate party that they want to have control over it. 'Cause with a blockchain at the moment, if we set it up as a completely public once, everybody has equal access and rights over…is that right Mike?

M: Yeah.

GD: Over what happens on the blockchain. But as Victoria said, there are opportunities to change that and make it a bit more private if you want to. So I guess what you're saying P05 is that you're asking the question, if you were to use something like this, you'd want to be able to see who has access in advance?

P05: Yeah, I probably would want to. It's almost like you're not putting your trust in one entity now, you're putting it in several, or being asked to kind of trust several, I guess, institutes and controlling bodies behind it. I just would probably feel more comfortable knowing who they are. It wouldn't necessarily put me off, I just, I suppose, would want to know who they were.

Or the other concerns I would have would be, would it just be UK-based, is it various countries, where's all the data being stored? So it's like how big is this network of health data that's going to be scattered across the…well, potentially the world I imagine, or is it just trying to control it in the UK if you can do that? So it's like, what are the laws that are going to be governing that data?

GD: Okay. I think maybe one of the essential things that we need to maybe explore is this issue around trust, and Mike or Victoria, you might be able to offer a bit more here. But the idea of the technology is because it's decentralised, you're not trusting in one particular organisation, you're actually trusting the technical architecture of the platform because it has these qualities of not being able to…or being very difficult to amend data on there, rather than putting trust in one particular organisation. But I'm not sure that's come across.

This is one of the things that we want to explore, because blockchain itself is quite a difficult concept to get your head around before you even start thinking about the platform. So this is really useful for us, P05, to know that.

P05: Good.

GD: Can you see any major benefits? Is there anything about it that interests you?

P05: Yeah. I guess it seems a little bit simpler from the participant perspective, and that you've just kind of got an app and you've got… If you kind of trust

the architecture behind it, it sounds quite simple that you can say what you're interested in, get the alerts through to tick a few boxes. It sounds much simpler than, I don't know, for people particularly like with rare diseases at the moment probably having to scour the internet for those research studies that peak their interest. Or you rely on the NHS to tell you what there is that's coming up and that you might be a good fit for. So in that sense, I guess it's a bit more empowering for the person that might want to participate in research trials.

GD: Aw, thanks P05, that's really helpful for us to know. So P01 have you got any initial thoughts about having seen that first use case scenario, any questions?

P01: Yeah, I mean, I would agree with what P05 just said about this probably simplifying the process. Like, everything sounded quite straightforward to me the way you described it, like quite user-friendly. One thing that just occurred to me, I mean, in this scenario the person is obviously…they want to make an active contribution to help and participate in trials. But I'm wondering, like especially for quite rare diseases, whether people who receive data in this way, for example, were receiving updates on trials that are ongoing – if I understood that this is also a part of it – that they would learn about new research? Or am I wrong here?

GD: Yeah, no, that's certainly one of the benefits, I think…

P01: Yeah, I was…

GD: …with the, try design into it.

P01: I was just wondering…this might be completely like off the topic. It just made me wonder whether some people then might end up feeling like some sort of sense of duty maybe to participate in such research, as in that they would see this contribution more directly than if it were a clinical trial just advertised, I don't know, on the internet. So basically, whether it would maybe create on one hand like a bigger incentive, but on the other hand maybe also some pressure to participate. I don't know, that's just my initial thoughts.

GD: No, nothing's off the wall though – just say whatever's on your mind – and it's all really helpful for us to know. So I suppose to kind of summarise, you're a bit concerned that people might feel obliged to take part in research perhaps? Is that what you're saying?

P01: Maybe. But I can see also on the other hand that obviously it might also increase people's incentive to participate and maybe would dispel some of the fear around it or the complications around it. So I guess there's two sides to it, as usual.

GD: Yeah, okay. Is there anything else that you wanted to add? Have you got anything else?

P01: Not right now, no. I'd be curious to hear what your thoughts on this are, and other people's obviously.

GD: Brilliant. Okay, thank you. P04, would you like to join us and let us know what you think?

P04: Yeah. So I think it's really interesting. I love it. I think it's a great idea. I can imagine lots of different situations where it's easier to invite people to participate. Like, in maternity services, you always get a bunch of leaflets or studies to join, and I can imagine that this would make it a lot more seamless for those that choose to opt-in or opt-out. And like in the situation now where we are in a global pandemic, you can see how with research studies, or research information, or with vaccinations and rolling that out, how knowing where data is to be stored or who you can donate that to is super-helpful.

I have questions. I don't really have necessarily very many concerns. So from what I understand, the blockchain keeps information of where data is stored and doesn't store the data itself. Right?

GD: Yes

P04: That's correct. So when I think of blockchains or how they are administrated, I've seen people have personal computers, but it's not necessarily like you're on your PC, some people run separate units that are powered constantly under this idea that that's maintaining, or that they participate in mining for blockchains or whatever, and they get a payment in return. With this system, are people going to be expected to have their own personal machines, and are they going to be receiving payments every single time they mine the data successfully? How does that work?

GD: The actual mining side of it is not really…it's all open at the moment. We've not actually started designing a platform, so we'd be very interested in what people think about that and how it would be powered through that. It may be that the mining side of it is done separately, so not directly by participants. At the moment we've got a completely open book, and we could design it in any way possible. So if people had a lot of concerns around that, then we would try and address those within the design process.

Mike, I don't know if you know anymore about the mining side of it?

M: Yeah. So anything is possible at this stage. As you say, you can have blockchain systems where actually the participants that are giving up data to the chain can actually be miners themselves and garner some kind of fee or reward for participating. So that's potentially an interesting incentive to get people to actually give more data.

If you look at proof of stake models where the more that you kind of… Well, proof of stake mostly is operating within a kind of cryptocurrency world, so

the more currency you have, the more chance in terms of randomised selection that you'll be able to get fees back based on your stake in the chain. But that can be diffed against more commission-based chains where you might have institutions that actually fund the mining and the validation of the transactions that are written to the blockchain. But yeah, yeah, no, it's an interesting design challenge that we need to address.

P04: So I understand that the proof of stake models are sort of more efficient when it comes to just basic computer power than the other form. So I guess my second question is that, I'm personally not sort of like personally orientated to the idea that we should value data privacy anyway necessarily. I think we give our data away to lots of different people all the time in quite identifying ways, and potentially even more identifying than specific bits of health data.

But I think my secondary query would be, is that even if the information sold in the first instance is relatively anonymous, it is dependent on the people that have that health data also use it for what the conditions are bound to. I know that in the US and stuff, for example, lots of problems have happened because of this secondary market of purchasing data.

So I think my concern is that it's reliant on conditions being as they are now when it comes to how people's personal data is stored. Like, I can imagine that if the NHS changes and we're moved to more private health models or if regulation changes, I can see how it becomes less helpful in protecting people's information as it signposts more people to getting that information. Am I making sense?

GD: Yeah, I can see what you're saying. I think part of the idea of the blockchain will be that you would have more control over that, so you could only authorise parties that you agree to to access that. But I see your point, and it's a very valid concern, that what happens to that data afterwards? But that will be outside of the blockchain. So I suppose that's always an ongoing concern for anybody sharing data in any kind of way, that once that data is shared, you're reliant upon that person acting with integrity and not…

M: I think…

GD: Go on.

M: Just to add to that, I think, obviously one of the capabilities that you have with a blockchain is you can map the provenance of data. So if you initially gave access to particular, I don't know, a pharmaceutical company, and then it transpires that some other organisation has actually accessed your data, you can go back to the blockchain and look at who you initially gave that data to.

Then there's kind of mechanisms there where you can investigate and work out potentially who the bad actor is in that process where that data has been

leaked to somebody else. Or, like you say, regulation has changed, which actually meant that that initial organisation that you gave authority to now has the right to just give that data to somebody else. 'Cause this is a big problem I think in the kind of gene profiling domain at the minute. So like you have these, you know, AncestryDNA, where they're basically reselling everybody's data but nobody really knows…nobody's really aware of it. So yeah, no, interesting point, for sure.

GD: Thanks P04. Is there anything else that you'd like to ask us?

P04: No, not right now, but I could go on further [without planning to 16:50].

GD: No, no, and we hope that you will do. So just to get everyone's initial thoughts really. So P06, have you got anything that you'd like to add?

P06: Yeah, I've got a couple of thoughts. Most of them are just meanderings. Well, I'll start with the plusses, that I think it's been said before that something like this would be good at highlighting rare conditions and bringing together separate bits of data that may be stored on different systems together. So that Person A in the South West and Person J up in Scotland can actually realise they both have like a deadly allergy to sunlight or whatever it is. In regards to questions, I guess the first one is about the smart controls that you'd be able to put on in regards to who can access data. Has it been done on this large scale before? Would it be effective? Is there a precedent for this?

GD: Not really. There are a lot of small use case research out there, but not on a large public scale like this before.

VN: And these were for health data. So there's lots of smart contract research in other areas, which we know that they work on a theoretical basis, and it would work. But if we would apply it to the health domain, there's lots of proof of concepts of those for the health domain, but we haven't really tried them out. One of the challenges would be to make this very easy and accessible for everyone, so even for people who don't really have a big technical knowledge, so that this can be easily set up even with the help of somebody at your GP, for example.

P06: Okay, okay. And yeah, my second question is sort of one of terminology. In the example you use, you said Chris donates healthcare data, and I just found myself tripping up on the word donate 'cause we don't normally think of that. Like, when you pop into your GP and tell them what's wrong with you and they put it into a system, you don't think, I'm donating my data here to some sort of big, large NHS system. You think, I'm just telling them what's wrong with me and then they can use that to figure it out. Then I got sort of stuck up on wondering whether we do donate information at the moment, but it's just an odd choice of terminology, I think.

GD: Interesting. Yeah, well that's really good to know. I guess it's quite subjective really in terms of how people perceive what it is that they're

doing. I guess with the idea of this particular platform, it's a bit more of a proactive response rather than just calling into your GP and telling them what's wrong with you. You are proactively saying, this is a bunch of information about me and my condition, or my health, my lifestyle, and I'm sharing this. Maybe sharing, I don't know, whether it's all semantics.

P06: I think part of the reason I'm just tripping on it is it's the idea that if you donate something, you're not expecting anything back; it's like a gift. Whereas with this, if you donate your data to the system, you would reasonably expect some sort of return, whether that is just the knowledge that you've helped someone else with a condition or whether you're actually going to get a tangible response from it.

GD: Well, that brings us to an interesting point. We're going to introduce some stakeholder perspectives in a minute and talk to you a little bit about what happens to that data, and how people might be either tangibly rewarded for it, or whether it's just an altruistic act, how that might be recognised. So we're going to come to that shortly, if that's okay.

P06: Yeah, yeah.

VN: I mean, does it…

P06: Yeah, that's everything I had.

VN: Okay, maybe I can just add on to this. So the idea of data donation as a term kind of came from the quantified self movement, if that rings a bell. So when people started to use digital devices to monitor themselves, there was like a movement of people who would then use this mainly quantifiable data to then see if they can derive self-improvement from that. From this movement, the idea of actually data donation kind of arose, and this was now then applied to different ways of how we might see data sharing in healthcare differently than we do now.

Because currently I think the system in the UK is set up like this, that you are not sharing data if you don't actively consent to it. Of course, but there's this also…there's a duty of care, and this duty of care is for direct health treatments. But then there's the research bit of it which the entities that hold data, for example, clinical or data trusts, can use anonymised data that can't be traced back to the individual and can then use for specific research after they've got ethical approval and went through a lot of hurdles to actually get to that point. So [they're voices overlap 22:34] in that thing.

But I think that it was said earlier that it's more the idea of actively doing it instead of this happening passively behind in the big healthcare system. I hope that clarified it a bit.

P06: Yeah.

GD: Is there anything else you'd like to add at this stage, P06?

P06: No, no, that's everything.

GD: Brilliant. Okay, P02, can we go over to you?

P02: Sure. I had a couple of questions and one thing that's positive. I suppose I would start with the negatives. I think one thing is if all the computers are linked and distributed ledger, is there an environmental cost to that? I suppose if you're one of those people who thinks that whether you're seeing it as an ethical thing you should consider it from a planet centric point of view instead of possibly just a person's centric point of view, I would probably want to know what the environmental cost was. I'm not saying I'm one of those people necessarily, but maybe to know that and what the benefits are to weigh it up against that.

I think the other thing was just that, how does this sit with the fact that it's a permanent record and immutable with the idea of having a right to be forgotten? There's some people who argue for that, I think. There was a case I seem to remember – I can't remember it – where it was established that maybe that's something that should be quantified, and if that's not possible with this technology, is that something that people might consider? I don't know whether that's relevant.

GD: It's absolutely relevant. It's a really big issue around adhering to the GDPR rights and responsibilities when we're looking at data. This is a huge challenge and hurdle that lots of different people in different disciplines, not just health data, are coming across. Because there is this issue of, if the data is immutable, the data subject, the person that's offering their data, has to have that right to have something either rectified or have something deleted. So there's all sorts of different kinds of technological alternatives that are starting to come out now, and a way around it is to design it into the platform, so privacy by design. That's something that we're looking into.

Also, speaking to some of the other professional stakeholders about it. So once we've done our first round of focus group meetings, we'll be conferring with different legal professionals who specialise in privacy law and also smart contracts and blockchain, to think around, once we get to a design stage whether we can actually adhere to the GDPR rights or not. So that's really important. And again, the environmental thing is really interesting actually. So maybe that's something we need to think about in the design process, to find out how we might be able to quantify the environmental impact of using something like this. So I'm really glad that you've brought that up, thank you.

M: Yeah, it's interesting. They've already done that around bitcoin, haven't they, where they can tell you what the equivalent cost is environmentally of like one transaction. So moving a bitcoin from one person to another works out something as like, I don't know, the energy consumption of one UK household per transaction. Which is obviously…

GD: Really?

M: Something like that.

P02: Yeah, I had read something like that before. It's terrible.

M: Scary.

P02: [Voices overlap…26:11]

GD: Absolutely.

P02: I think the positive thing that I've noticed is, like, just to share some information. I'm someone who has a rare disability, and I live quite close to a research centre. I've given a lot of data and done trials, and the amount of consent that you have to do is quite onerous really. And the clinicians, you see how much time they've given up when you participate again and again and again.

So it would be nice if there was a way to streamline that, because you're conscious of how much time that that takes every time they have to go through the same process and make sure you've understood, and if they want to reuse it, they get in touch again. I think if there was a way to control that with the technology and just give the permissions and control who you trust, that would be probably a good thing, I think.

GD: That's great. Thank you P02. And you say that that's a positive, but I guess from someone else's perspective, that might also be a negative in terms of giving consent and wanting there to be more time spent on that. But we'll maybe come to that shortly. P03…

P02: I think it comes down to, if you trust somebody enough, you might be able to say to them that I trust you repeatedly to use this data however you want, but if you've got that control about who that is. It's something I would be interested in, for sure.

GD: Right. Oh, thanks P02. Thanks for sharing that with us, that's really helpful. Okay, P03. Sorry, last but not least, have you got anything you'd like to add?

P03: First thing to say is, this sounds really interesting, so I'm really glad to be invited to be…

GD: Oh, good.

P03: …a very small part of it. The first thing that came to mind for me, I think regulators will bite your hand off to be involved in this, because one common issue to professional healthcare regulators is the time needed for their fitness to practice processes. So often those processes are held up by administrative burdens such as getting the accurate health record or the

relevant health record from wherever they are contained because they are pertinent to the fitness to practice case. So that is one major benefit that I would particularly identify, that it would really help to speed up that fitness to practice process.

Questions I have? I suppose it is more questions again rather than concerns. In relation to a kind of information governance point, I suppose the principle of only giving as much information as is absolutely necessary, or third parties only accessing as much information as is absolutely necessary, I suppose, who is the decision maker about what is absolutely necessary in this kind of transaction? And is it the individual donating the data, and if they are, they're going to have to be able to go into quite a lot of detail. That might be quite burdensome on the individual, not just in terms of time, but in terms of understanding as well. You know, the necessity for that data being limited.

For example, I'm just thinking about a situation where I go on an app and I say, right, okay, I [welcomed 29:21] a complaint at a professional regulator, the fitness to practice team are investigating it, I want them to access my health data from Date A to Date B. That might include quite a lot of stuff that's not pertinent to this particular appointment with this particular healthcare professional that I want them to investigate. So the granularity that would be required, I would perceive that on the face of it to be quite in-depth, I suppose.

Another point, another question, I suppose that I have is I think about the failed Care.data project. Obviously, a lot of that being a lot of the disquiet, the discomfort with that, and the reason for lack of sign-ups was attributed to patients not having the appropriate trust levels in the NHS authorities, et cetera. But some of the comments arising out of that from patients were, well it's not so much them, it's the data that's the problem, it's the intimacy of that data, and it's the detail that it goes into. So whilst a lot of the discomfort around an untrustworthy organisation might be allayed by this, there's still the added element of, it's really, really sensitive data.

And in relation to the kind of rare disease aspect, in the vast majority of circumstances, no matter how much you anonymise it, it's identifiable. If you are one of ten people in the UK with a particular disease or illness, your data is going to be identifiable because there are only so many permutations that it is known to exist in.

So one last point in relation to data itself. I suppose a question I have would be, [what is their 31:22] data, what do we [need 31:23]? In the new COVID-y world where remote consultations have skyrocketed and traditional health records are being supplemented by recordings such as this one, would that be included in the data? The initial assumption would be that it wouldn't because obviously a recording is really quite identifiable and you can put a face to the information that's being discussed, but if that's an important consensual part of that individual's health data, won't they be able to donate

it if they so wished? Yeah, one practical benefit and lots of kind of musings and ramblings from me.

GD: Thank you. No, that's given us some really interesting things to think about. I guess the central thread of what you've been saying, a lot of it is around that granularity, and it's deciding in advance what kind of options people will want and what level of control people want to have over specifically exactly what kind of data we're consenting to share or donate within a platform such as this. When we move on later to talking to people in the general public, we want to find out more about the levels of control people would like to have over what they share and with whom.

And in terms of the anonymity, yeah, it's a really important concern, and I hear what you're saying. The idea around this is because none of the data at all will be held on the blockchain; any kind of interactions regarding the data would be off the blockchain. So this is a facility really. It's a platform to enable A to get in touch with B, and then once that's off the blockchain…and again this is another thing that's open for discussion, is then what happens and how do you regulate that. So you brought some really important points. Thank you P03.

Does anyone have anything else they want to add based on some of the contributions from your colleagues?

No? Okay.

Victoria, do you want to introduce the perspective part?

VN: Yes. So let's see if we can…ooh, one too much.

[VN reads out scenario/stakeholder perspectives.]

GD: Thank you Victoria.

VN: I will just keep them up so you can have a re-read if you want to.

GD: I'm just aware of the time actually, sorry, guys. Do you want to just take a quick break now? Does everyone need a break? Or are you happy to carry on? Is that alright if we carry on and push through just to half past two? Is that okay? Brilliant okay.

So has anyone got any strong views about, say, for example, the pharma company perhaps paying people for accessing their data or be rewarded in some kind of way? Was it P06? Did you say something about that? Or was it P02? It was P06. It was you.

P06: I'm not sure if it was me, but I do have a thought about it. Which is just obviously pharma paying patients for anything is problematic straight off the bat. But I'm guessing it's linked to this idea of individuals being able to put their data up themselves and an idea about quality control. How do you

know that what people would be putting up on the system is genuine, especially if there's a potential financial reward for putting all their stuff on there? How do you do this quality control?

GD: Yeah, that's a really important point. What about if they were to make a donation to a health charity? Does that skew your responsibility?

P06: I mean, it may deter people who believe…or who are after a financial reward themselves, but it might encourage people who think pharma companies have way too much money and they should give it away anyway and then they can just redistribute it via this mechanism. Probably a very new…

GD: It's a really important… It's a really important point though, how do we ensure that the data that people actually contribute is what they say it is.

P06: Yeah, and especially if this is built on an idea, a sort of algorithm, you know, data-in, data-out. If the data you're getting in becomes corrupted, then what you're going to get out is going to be equally if not more corrupted.

GD: Thanks P06. Has anyone else got any views on that?

P05: Yeah, just related to that point I suppose about quality of data. Is it just patients that will be submitting their own data in the sense of they need to understand what their diagnosis is, what the implications of it are for them, or is it going to go through their GP or somewhere so it's kind of being put on their medical record to ensure accuracy? 'Cause I know a lot of patients don't actually have a thorough understanding of their condition and may not be sharing the right bit. I suppose it depends what's being asked for.

But in terms of, if it's like what medication are you on, for example, they might not quite know what they're on or why they're on it or anything like that. So are they responsible for sense-checking their own data that they're submitting, or will there be any healthcare professionals involved?

M: Yeah…

GD: Yeah, go on, Mike.

M: Sorry, yeah, I was just going to say that potentially we could have functionality where you can delegate control of your data, and so that might be to your GP or whoever that might be, you know, some kind of health practitioner that already is helping curate that data if it's some kind of treatment. So yeah, no, that's an interesting point.

And then just related to the one previous about kind of crap-in and crap-out in terms of the data quality. There's a big area within DLT research around oracles and how you verify [real-world 40:32] phenomena data and mechanisms to kind of do that. So particularly around representing digital identity within a DLT, there's mechanisms where you could get your friends

to verify certain credentials about your identity before it's actually written to a blockchain. So potentially, in this scenario, we could use health practitioners to actually verify data before it's actually written or accessed 'til the data is actually given.

GD: Thanks Mike. How about the other perspectives, the patient or lawyer, has anyone got anything that they'd like to add or anything that's resonated with them particularly about those?

P03: Just an additional quick point on top of the records issue, is that a lot of healthcare professionals are still really bad at making records and they don't do them in a uniform or consistent way. That's particularly true for, in my experience, from the allied health professionals rather than doctors, nurses, that kind of thing. Because we see the complaints to fitness to practice and the regulators of full record-keeping, of inconsistent record-keeping, or record-keeping that is not legible even by another practitioner when they come to take over the case.

So it does seem a bit reliant on the healthcare professional being exemplary in their documentation for this to really work. So I wonder if a way to get around that would be to get the regulators on board and reinforcing their guidance and requirements around record-keeping.

GD: Right, thanks P03. Yeah, that's a really important point. It relates back to the crap-in, crap-out scenario as well. What about if somebody donated some data from an app, so a fitness app or something like that, that they had, so it was data that was collected on their phone so it wasn't anything to do with a healthcare professional? What do you think about that?

P03: I think you've still got the same issue, haven't you, of potentially corruption by user, in that different people will record their data in different ways. That's particularly the case if it's qualitative rather than quantitative, because obviously quant can be benchmarked and relatively sense-checked but qual less so, I guess.

GD: P04, did you want to say something?

P04: Yeah. So I think one of the things that I think I'm getting confused about is that the quality of the data that's produced by the healthcare professionals isn't really necessarily important to the function of the blockchain – from my understanding – because that quality is going to be what it is anyway. The blockchain signifies where that data is, so the blockchain isn't necessarily dependent on the quality of the information. I don't feel like that's what this generally does.

Then when it comes to sort of like sharing information from apps and things, I feel like that's important. Like, Google can tell if you're pregnant before you can in lots of cases and articles that I've read. Google can tell if you're going to have a heart attack in the next year. Like, this is a legal issue that they're having in the US with medical health insurers getting access to

people's search history because of what it can determine about their health outcomes.

So this is what I mean when it's like, this technology is reliant on that it doesn't prevent people accessing certain things in the future, so like you're saying that how you share the information is going to be an important function of blockchain. I think one of the things, like when it comes to health data that you may share from watches, the algorithm in itself is quite good at excluding false information. Like, health things that monitor heart rates and so forth can usually tell when it is a manufactured result – from what I understand of it – so that seems like a good thing to share. And we already are sharing that information with our iPhones and so forth anyway, so to be able to share it to a better use seems fair.

I think one of the concerns is that there is like a potential for the generalisation of consent when we allow our cookies and so forth, or when we sign the GDPR waiver. Everyone acknowledges that we're not actually reading those things and we sign away our privacy relatively ad-hocly all the time anyway. So I think this technology could have a potentially other function of making people more data literate in that they have to think about it more when they are deciding how it's used, which is what I think is a good thing about it. It's great.

GD: Thanks P04.

P04: [Radical perhaps 45:53].

GD: No, it's not... It's really good to hear an alternative perspective. Has anyone else got anything they would like to say in response to that?

End of transcript

**File Name(s)**	56347_CDIP-FG_13-10-2020-Part-1<br>56347_CDIP-FG_13-10-2020-Part-2
**Total Recording Length**	68 minutes
**Number of Participants**	8: FG2 (P7-P12)
**Final Word Count**	9,481
**Total Number of Pages**	21
**Date Completed**	28 October 2020

[56347_CDIP-FG_13-10-2020-Part-1]

GD: Having heard about the idea of it, a data donation platform, I'm just wondering, initially, what do you think might be the major…the good points and bad points about it? What does it make you think about?

P07: I was just thinking then, if you, say, get contacted by a pharmaceutical company, can you kind of…will they have access to all of your health data? Or can you specify which bits they have access to and which bits they don't? If that makes sense?

GD: Victoria, do you want to answer that or do you want me to?

VN: Yeah, so you can specify what kind of data can be accessed by any person. So in this scenario, Chris was just enabling notifications, like if a study is being done that is connected to his disease, that he has an interest in maybe taking part in it. So that was all in that case, and, yes, it will be very granular that you can just decide who gets what kind of data.

GD: Thanks, P07. Has anyone else got any sort of advantages or disadvantages they can see as a result of using something like this?

P11: I think one of the benefits is that people actually have access to what data they want to share and they actually know what specific organisations…what sorts of information they take from them. Because I don't necessarily know of any other way, at the minute, that you can do that, so you don't know where people are finding information about you.

P10: My major benefit is the fact that it clearly makes…it would make running research on rare…the rarer the disease, the more beneficial the short platform would be. And obviously it would make it a lot cheaper for new drugs to be developed. But one question I do have is, you know you can…? Who would be able to see what data has been transferred? Would you have permission controlling, like, the general public can see that my data has been transferred between these people, or just other researchers?

GD: It's kind of all up for discussion at the moment because we haven't actually built anything yet. This is why we're doing the focus groups, to think around what level of visibility would people want. But generally, it just would be anyone that's involved in the actual platform itself. So it would just be you and if there were, say, for example, a regulatory body that was involved in the platform that oversaw everything that was going on, they might be able to check. So like an ethics committee or something like that, and the company itself. But at the moment, it's all really up for discussion, so I'm wondering, what are your thoughts around that? Who would you want to be able to see those transactions?

P10: I'd want basically everyone to see it, because you'd end up with, like, crowdfunding effort, crowd-led ethics at that point, rather than having an independent committee that might be beholden to other people. But I know

that some people might not want the crowd, the sort of anarchy of ethics at that point.

GD: That's really interesting, thank you. Does anyone else have a view on that?

P09: I think it's good, but would you have the right to withdraw your data at a later date, if that makes sense?

GD: Do you mean if you gave permission to a company, say a pharmaceutical company to access your health data, and then you changed your mind?

P09: Yeah. Could you take that access away from them at a later date? If that makes sense.

GD: Yeah. I guess you could retract it and not let them take the data. as an ongoing thing. So are you saying that you would want the capacity to be able to say, I want that data back, I want to know that [voices overlap 0:04:23] no longer using it?

P09: Yeah, just like the option. If that makes sense.

GD: Yeah, and that's an important point for us to consider. How about anyone else, how do you feel about that? If you gave permission to someone to have your data and then you changed your mind, would you want to be able to have confirmation that it's been withdrawn?

P07: Yeah, definitely.

P08: Yeah.

P11: I have a question, though, sorry, just for clarity. It says that the data will be anonymised, so I don't know if you've mentioned, but how would, like, pharmaceutical companies, like in that example, contact you if your data has been anonymised? Do they get sent your information and everyone else who doesn't get to know, or do you know what I mean?

GD: Yeah, so the idea of the platform is that you're using that platform as a way of kind of connecting with other organisations, but the actual data that they're requesting from you would be sent outside of the platform. So it would be within whatever mechanism that they would normally use. Is that right, Victoria?

VN: So for that example, there are different ways of doing it. So they can, for example, just make a request of everyone who has liver disease to be contacted, but they will not get the individual names and contacts of it, but you will then receive a notification that there is this out there. And if you then choose to contact, to go back to them, that's your decision, if you like, but they will not get information about you personally.

P11: I see, thank you.

GD: And what about the anonymisation side of things?

VN: It is for me, or…?

GD: Yes, sorry. I have a question for you.

VN: So the idea of this kind of blockchain is that you have an immutable record, and so far, even though the data is not being transferred but only the record of the data, everyone could see the transaction of the data. But it's very hard to actually look back, like to draw this back to you as an individual person, so people can see transactions, but not necessarily reidentify you.

P11: Okay, thanks.

MH: Can I just ask on that, how would you feel if these types of transactions were actually associated with your identity? Would you be concerned?

P11: Personally, I think it would be a little bit of a concern, in the sense that unless you give them permission, like you've already said that you want to share your information, it leads to questions. And I mean, there are ways to make it secure, but it leads to questions about who can access your data and is there a way if, like, god forbid, anyone was able to get into the system and find your information. And then find out all the rest of the information about your health and all the different conditions and things like that. It would be a bit of a concern.

GD: What would make you less concerned? Is there anything that would reassure you about that?

P11: I think just really being able to opt into things. I mean, if you're like the guy in the example, if you were really keen to try and get someone to help you with a specific condition and you got a generic email because it's on the system, even though it's not you in particular, it sends you a message based on the data. And then you get to choose that you want to partake in the study, or something like that. I think personal choice is important.

GD: Brilliant, thank you. Can anyone see any drawbacks? Is there anything, other than around the security, that would make you feel concerned about using something like this?

P07: Not thinking about me personally, but if, say, you have vulnerable people who perhaps aren't… I don't know what act or whatever it is, that don't make decisions for themselves because of their condition, I wondered whether there would be protections in there for people like that so that… I don't really know any examples, but whether there would be any protection for people like that.

GD: Okay, so thinking around how people have capacity to consent.

P07: Yeah.

GD: So some kind of safeguard in that perhaps a GP or a healthcare professional could have some kind of identifier on it.

P07: Yeah.

GD: Okay. What about anyone else? Has anyone else got any ideas around who might struggle perhaps to use something like this?

P11: This is a little bit of a generalisation, but it tends to be like older people might struggle with using apps and things. So it's kind of like how is that accessible and is there some sort of like…I don't know, like video tutorial to teach them how to use it? Something as simple as that, so it can guide them and they know the benefits and drawbacks of it themselves, maybe.

GD: Yeah, that's a good point, P11, thank you. What about you personally, what kind of controls would you like to have over the health data? So earlier, we were talking about healthcare professionals and people in the NHS, but also in wider society, companies and app developers might be able to…you might be able to share your health data with them. Do you have any particular ideas around how you'd want to control that?

P11: I'm not so sure, actually. I think, again, it's just if someone approaches…if you need something in particular and somebody approaches you, again, it goes back to what I was saying about how you kind of need to know what sort of information they're looking for. And then need to know that you yourself, and not anyone else, are passing on that information. I think that's just the biggest thing for me personally.

GD: Great, thanks, P11. Were you trying to say something then, P09:, sorry? Okay. So thinking around now, how would you make a decision about saying yes or no to somebody wanting your health data? What would you take into account?

P10: The main thing I would take into account, and this is a very personal thing, when I tried to apply to join the Navy, they refused me basically on the fact that I'm on the autistic spectrum, but not in any noticeable way, but they still refused me. That's the main thing I would be concerned about, is potential employers seeing something and just ticking a box that stops me, but not actually seeing whether it stops me. That's the only real issue, that's the only thing I would think about if I was stopping data getting transferred. I mean, something like this would have been very useful when I was trying to apply to the Navy because it would have been far easier to find medical records.

GD: That raises a really important point around a person's right to share different parts of their own health data. So I'm wondering how you feel around… When we were talking before about choices and controls, if you would want…how would the ability to actually say, I'm willing to share this part of

my health record, but not this part with this person or that person? It sounds like maybe that might be something that could have been useful in that instance. Would you want to be able to be that specific and say, I'd like health professionals to be able to see my health record, but a certain amount of sensitive data I don't want to share with anybody?

P10: Yeah, I'd want that sort of level of granular control. And the problem is, though, it's like I could say, well, I don't want anyone to see my PDD – I think it's PDD, I can never remember the acronym – diagnosis. But later on, I could have a diagnosis that links me with PDD. So how would you end up with futureproofing choices? Because I could just say generically, all mental health conditions, but I might not care if I end up having a stroke. If that's technically a mental health condition, I don't know. Don't laugh at me!

GD: That's a really important point. So how do people feel about being able to futureproof what you choose or what you control? At the age of 25, and you might have a different view on it when you're 45, for example.

P07: I think if you could have something built in so that, say, every amount of time you have to go back and look over what data you actually want to share, so that if you… Because I know I just wouldn't check it and then just not like sharing lots of things, or sharing lots of things that maybe I don't want to. So that if you had to constantly…well, not constantly, but every so often you had to kind of update your preferences for data sharing, just so that you're only ever sharing what you want to, if that makes sense.

P09: I was going to say the same as P07, say if you got like an email like every few years, check what you can share and stuff so that you can change it when you want to.

MH: So would it be interesting then to be able to kind of set conditions, such as expiry dates on agreements that you make in terms of who you share what pieces of data with?

P09: Yeah, I think that would be a good idea.

VN: Would you also want to have like an automated thing that if you don't reply to those changing of the settings that it just puts a block in that no data is being shared?

P09: Yeah, sort of. You know when you get an email and it's like over a year since you've looked at them, so it's like, do you want to unsubscribe, that type of thing, then you get that option.

GD: What about if you could be completely open and say, I'm happy to share my data with anybody that wants to look at it, so that you're not constantly getting asked? How would you feel about that as an option for people?

P10: Options are always good. I wouldn't take the option but it's always good to have them.

GD: Okay.

VN: Are there any other reactions to the scenario in itself? Were there any thoughts that haven't come up yet or your first reaction to those which may be not related to our questions?

P08: I was thinking about…you know like with children under 16, obviously their parents could consent for a lot of things, would it be a case of no one under the age of 16 being able to have it? Or would it be, if they're under 16, their parent chooses for them, like the consent of what data gets shared? And then they change it when they're old enough to? Because it's like if my mum chose to share all my data when I was younger, I wouldn't want that personally, so when I'm older, I'd then want to change it. But would it just not be an option for children to have it shared or…?

GD: That's a really interesting point, P08, and for discussion. How would you feel about it? Would you want it to be open until you were 16 so you could make you own mind up, or not?

P08: Because the thing is, it's like even under the age of 16, like a lot still happens, like within the healthcare, so I feel like personally, I wouldn't want my data shared just because my mum said that it could be. So I don't know whether it would be worth having it for anyone under the age of 16 when they can't really give their own consent or not.

GD: What about everybody else, what do you think?

P07: I agree with what P08 said. The only thing is if, say, you didn't have the option for under 16s, say, if you've got the thing in this scenario with the pharmaceutical company, if an under 16-year-old has a rare disease that they want treatment for, if they can't use the platform, that means that they then can't maybe be part of trials or anything that might help them personally. So I think if you had like a limited account for people under the age of 16 so that there were certain things they could and couldn't do, I guess the parents could say they can… Well, like there's like a limit on what their parents can share, just so that you get like people aren't getting decisions made for them, but also if they really need help with, say, a rare disease, then they can get that help.

GD: Thanks, P07. Anyone else? No one with strong views on that? Okay. We're thinking around your perspectives now, but we've also got a collection of other ideas, other concerns or benefits that other people might have regarding this. We just wondered if you just take a look at these and let us know which…just identify maybe one viewpoint that you think is most interesting or concerning and highlight that to us. Have you got the next slide, Victoria? So we've got Rose here, she's got concerns about app developers selling data to other companies, but not asking for her permission, and that's something that she's worried about. Or May, "could

a company say they're going to anonymise the data and then reveal her identity to the government without even asking her?" The next one.

And then Pavo, he's got some questions around how does he know if an organisation app wants to access his data? How does he know that they're actually a legitimate health company? And Jonas is a parent, and it goes back to the earlier comment that you were making, that he's got three children and does he have to then manage their data? And what if he makes a mistake or he shares his children's data? He's going to feel bad about making that decision. And he's wondering if the GDPR might help, if data needs to be corrected, or what will happen if they want their data to be deleted?

And then we've got perspectives from pharmaceutical companies, which goes back to the point you raised, P10. Is it's quite expensive and it takes an awful lot of time to get enough participants to do a clinical trial and it can put people off doing different pieces of research. Especially for things like rare diseases where they might not be a huge population affected by it. And they're saying that could really help them. And also, that they'd be happy to pay for that data, perhaps make a donation to charity, or actually pay the patient themselves. How would you feel about getting paid for sharing your health data?

And then finally we've got Mahmood, who's a lawyer, and this is another thing that was raised earlier about consent. So if there's no other witness, like a healthcare professional, how can we be sure that that person was actually competent enough to give their consent, or they didn't do it when they were drunk on a night out?

So we just want you to have a think about some of those comments and perspectives. And if you don't mind, each of you, if you would like to just maybe highlight one that has made you think about this in a different light perhaps.

P07: I think the one on the… I don't know where it is. The one about the government. But I think that it kind of ties in with the other one, giving people the kind of legal right to their data as their own property and that the government can't access it in any scenario without consent from people, but you'd need a proper protection on it. Because I guess, in a way, the government could just pass a law or whatever making this all kind of available to them. I think that is a massive concern for if…you never know what will happen in, like, 15/20 years, and if you get not the best government in, that wants to kind of use your personal data, then that's kind of worrying. And as well, with the paying for your data, I think that would be like a really good idea, because giving people the right to their own data and basically their data being the product that they can sell would be a really interesting idea.

GD: Has anyone got any thought about that, paying for your data?

P11: I thought the paying for the data one was quite interesting. I mean, as a question leading up to my point, I'm guessing all this information is going to be collected from GPs, so you can' make up any data just so you can get some money out of it. Do you know what I mean? Is it all going to be like actual data from GPs and things like that?

GD: That's a really interesting point, in that there's also data from apps and Fitbits, and all sorts. There are more and more health applications/lifestyle applications out there and data is the new gold, it's more valuable than oil. And so would that incite somebody to falsify their data just so that they could sell it?

P09: I think you'd get the odd few people that actually would do that. Like if they're struggling for money, there are so many different scenarios that the would do that just for that case, which would also make them quite vulnerable. So concerns about consent as well, like just giving your data out because you're in a vulnerable situation and you need money, kind of like makes a problem with the consent. If that makes sense.

GD: Yeah.

F6: I think on top of that, as well, is would there be like an age restriction? Because if you're like…I don't know, like a student and you need some extra money and you share that data, and then in a few years' time you regret that and now it's public to everyone, sort of thing, maybe.

GD: Are you saying, just reflecting that, that you perhaps think there should be some safeguards around…?

F6: Yeah, it's like something, maybe like an age limit or something like that, or just like extra precautions to take so that the kind of information people share at one point in their life might not necessarily come back and then they regret it.

GD: What about if they made a donation to a related charity? So say we had that scenario with Chris and the pharmaceutical company has made a donation to the British Liver Research Trust instead of to a person? Has anyone got any thoughts about that?

F6: I think it's probably better in the sense that you know you're helping a charity and I think people would probably feel worse if they've made it up, if it's going to a charity that's actually trying to help people with these conditions. So I think there's a benefit to that.

GD: Okay, so they've got some kind of moral obligation.

F6: Yeah, pretty much.

GD: I guess earlier, P10, you were saying about crowdfunding and things like that. Can you see a use for incentivising people to share their health data if the charity would be rewarded?

P10: I don't feel like it would be a very big incentive for the charities. But I have no idea, I'm not particularly involved in the health charities, beyond going to the furniture shops. I guess we don't really know what the health charities do, beyond a set of furniture shops. I guess there must be lots of research, but the sort of people who would falsify data, I don't think maybe would really interested in if it was going only to charities. I also can't really see…I'm trying to think, but I can't see any way you would really scale a falsifying data attack. Because surely it would be pretty easy to tell if you're randomising data. And you can't exactly do a [granny farm 0:27:58] data because it needs to be randomised, it needs to actually be of the illness.

GD: Okay, thank you. P09, were there any perspectives that really stood out to you?

P09: Yeah, it's similar to what I said before, like the one about the consent with the lawyer. Because obviously consent is really important for things like this and you can't really tell over the phone or over an app if someone is vulnerable or if they're drunk or… Like what situation. Like even at the simplest thing, as like just having an argument can affect what you want to do. So something you could do to adapt that would be to say, like, ask for consent one day and then…but not take that consent, but like ask in three days to see if they have the same…they want to do the same things. So they have time to think about it before they make that decision.

GD: Yeah, that's a good idea. Has anyone else got any ideas around the consent side of things, what sort of safeguards or controls might be useful?

P10: Especially the checking up point of view, it could maybe have part of the GP's job is then to help them set it up and make sure it's set up correctly. And then they would also take consent at the end. So they may have set it up like a month ago, when being drunk, but then afterwards, the fact you have to go to the GPs and had it set up properly and have them sign for it. Yeah, I like P09's idea.

GD: Ultimately, who do you think should have control over whether you consent or not?

P10: It's not consent if you don't have control over your own consent.

GD: I'm just thinking about some of the comments around more vulnerable people. So do you think it would be a good idea for people just to be able to give their consent and have it checked, say, in three days' time and double checked? Or do you think that some other third party should be involved if somebody is vulnerable, to say yes or no, that person can give consent?

P09: I think it would be handy to have like a health professional check, say like mental health reasons and stuff like that, whether your consent is… Like whether you should really give consent or not, if that make sense. Because some people obviously don't really know what they're doing, like there are just lots of different conditions. So maybe having like your GP or someone, who knows your background, to have a look before it's actually put on there for everyone to see and just check they are not vulnerable in that instance.

GD: Okay. What about the comment, making sure that organisations who access data are genuine, has anyone got any concerns around that?

P11: Would an organisation who accesses information need to go through…almost like a screening process to make sure they're real? Because if anyone could just sign up to it and say, I'm this organisation, would they be able to access your data? So maybe going through a process to confirm their identity might be useful.

GD: Okay.

P10: I mean, at that point you could even have it be like an online journal of data behind like a paywall of, like, seven [inaudible 0:32:09] that only the universities and big companies pay, or the [inaudible 0:32:16], and that stops a lot of small people because, you know.

GD: Would that stifle innovation, do you think? So like smaller app developers who might have a brilliant idea of collecting the data, but aren't financially able to do that, would that bother you, or not?

P10: Well, like the fact that it would be behind a paywall would mean the smaller developers can't do things? I mean, that does stop that specific thing, but I have no idea what sort of pay. You could probably balance the cost of the paywall [inaudible 0:33:05]. That would be really difficult, though, to do.

GD: Are there any organisations that you particularly trust that you would be happy for them to verify who can and can't access health data?

P07: The NHS could do this, or one of the health organisations like Public Health England or something like that, just to verify that people are actually companies and what their reasons are for wanting the data in the first place.

GD: So linking that back to one of our questions about what information you'd like to have before using a platform like this, would that be something that would help you to make that decision, or would it not really matter? So knowing that a particular organisation has overall control over who can access or not access a platform.

P10: They could just have like a grading system, a five star grading system of companies. And if they think the company is shady, they put a really low grading on it. But the company then can, if people then decide to go with it, it can then slowly build up its rating. And if a company manages to lie, there

could be pretty bad penalties for it, but I have no idea what the penalties would be.

GD: That makes me think about TripAdvisor or Amazon and things like that. How would you feel about if other patients or other people using the platform could rate people that use it and perhaps give reviews?

P10: Well, not like the people who use it, but the companies that are accessing the data. I mean, it would be a bit weird rating the patients, like, your liver disease is bad. I mean, rating a shady company, because that would also allow small companies to join on, but it might make them very slow at starting, but the review process would hopefully give them like a medium rating, I don't know.

GD: What about you, P07, would that make you more inclined to share your data with a third party, if there was some kind of system in place that either reviewed them or gave them a rating?

P07: I think so. I still think it would be good to have screening to make sure that every company is approved that wants the data. But also protections against false reviews and people who have maybe been kind of pressured to give certain reviews. Say, for example, if there's bribery and stuff like that, if there's something like that and companies wanted good reviews, they might end up just trying to encourage people to give them good reviews.

P11: I think as well, with the rating sort of thing, there might need to be some sort of safety measures involved. Because a few people might end up learning the hard way, that a company is not trustworthy, and then they've given valuable information to a dodgy company, or something like that.

GD: Okay. Has anyone else got any strong views around that kind of system?

MH: Can I just ask on that, on the ratings and the reviewing kind of model? Would you trust reviews more from an actual human being or more from a system that could potentially detect misuse of the data?

P10: I'd trust both of them equally. It would really depend. If it was a system, especially with an algorithm, I'd want the algorithm to be open. As in you'd know if it was a machine learning, you knew what…they would put what they were teaching the algorithm on. I'd specifically want to know what they were teaching it if it was an algorithm. But I'd trust them both equally.

GD: Okay. It might be a good time to take a really quick break now, just for five minutes, and then we'd just like…

[56347_CDIP-FG_13-10-2020-Part-2]

GD – reads out 2nd scenario , Health cert app

VN: So also in this scenario we would also like to highlight three different features. So first of all, it's privacy preserving and minimal data sharing. So once it's set up, the system doesn't check for the identity of Alisha but it just checks the likelihood of the immunity passport being correctly issued. So this way, people can keep their privacy and don't have to prove their identity all the time, and so minimalizing possibilities of overarching surveillance.

The second thing is that it's tamperproof, so the records in the systems are really hard to fraudulently change without somebody noticing. So this is an added layer of securities that make sure that these records and passes are also correct at the time when they're issued, and that this is a way of checking this.

And the third one is that there's no central authority that controls this, so it enables all the parties involved, authorised to use this platform, to interact directly with each other without having a central third party act as an intermediary. So traditionally, you would normally have the NHS in the UK handling your data and other data control, but here it would mean that multiple users and institutions have control over that and not just one. So back to you.

GD: Back to you. Again, as we did for the first scenario, has anyone got any initial thoughts about the potential benefits and drawbacks of using something like this?

P07: Kind of just thinking about it, so it's like immunity to, say, like COVID-19 or something like that?

GD: Yeah.

P07: The only thing is, because I know that it's selling the scenario of something about her clients getting the app as well. In terms of, like, if somebody doesn't have access to…hasn't been vaccinated or hasn't got immunity to it, would that mean that they could be blocked from accessing certain aspects of society? If that makes sense. So if you had somebody who, say, couldn't be vaccinated or something like that, then would that then mean they have less access to things that people who could be do, if that makes sense?

GD: Yeah, that's a really important ethical issue around the whole idea of having an immunity certificate, more generally, on any kind of application used on any kind of platform. I guess what's different about this is that it would put Alisha in control of how that information is shared, so she could choose who she shares that information with, or not with. But yeah, the point that you're making is an important one for society generally, in terms of accessibility and excluding people from particular places, yeah.

VN: We already do have that to a certain extent. So, for example, if you are HIV positive, lots of countries actually bar you from entering the country themselves. But it is an important question to then ask, okay, if we set things

up like this, and then do we expose people with it? Or is there a problem with having just…temporarily not having access, or is this going to be permanent, and things like that? So it's a good question.

P10: Sorry, I'm a bit confused about the actual scenario. Is it that only Alisha goes back to work, but only requires people who go to her salon to have documentation that they're immune? It's basically that, but the app's doing it instead of just a piece of paper? Because I'm surprised you couldn't just do that with a piece of paper. And it's a bit like you've just got, essentially, on your ID, you've just got an ID certificate at that point. I agree that it has got the problem that P07 said, that you can exclude people, but I don't see any reason why the app would make that any more of a problem than the [modern 0:07:35] system. It will make it a bit easier but…

GD: I guess one of the things about having this kind of app using blockchain technology is that it enables…it's much more difficult to be fraudulent. So with a piece of paper, it might be a lot easier to fraudulently copy that certificate, but because it's using blockchain technology and it has to be verified within the platform, then the person that's checking that, that's enabling someone access to a place or not, may have more reassurance that this has been verified, that it's not a fraudulent certificate.

P09: I think similar to the first scenario, if it's on an app, is that kind of similar to track and trace? Like how would that work for the older community who don't have access to that? Because if they don't have access to that, does that just mean that they just wouldn't be able to go into the salon because they don't know or…? I know my nan wouldn't be able to download that app, so where would that leave people without the access, if that makes sense?

GD: Yes, that's an important point in terms of accessibility and ensuring that it's available or usable for most people. Is there anything that you think we could do to encourage older people? Sorry, I shouldn't generalise, people that aren't as familiar with digital technology to use something like this? Do you have any ideas around what might be helpful?

P08: I don't really know, because unless, let's say, I was to go out of my way to buy my nan an iPhone, I wouldn't be able to give her that opportunity anyway. And obviously it would be very expensive to go and buy everyone who doesn't have a smartphone a smartphone so that they could do it.

MH: What about the use of biometrics, or say your nan could go to the hairdressers but have, I don't know, her retina scanned, and then that could identify her in the system and show that actually she has been to a GP and has had the immunity test? Would that concern you or…? Obviously at that point, then, she doesn't actually have to carry a mobile device.

P08: I didn't even know that could have been an option, to be fair. Yeah, I guess that would help.

P10: Retina scanning feels a bit too fancy for a small hair salon.

MH: Yeah, I guess the bigger point there is around the fact that she could potentially demonstrate her identity without having a device. And there are various techniques you could use to be able to support that kind of interaction. But then obviously you kind of tie back into then the issues around associating identity with the data.

GD: What about some of the other features? So not only does Alisha use it to confirm her immunity, but she could also share her blood group, if there was a capacity within this kind of application. For example, a paramedic or somebody could access her health record in an emergency and know that she was O positive. Or she goes on holiday and wants to enter a country that will only let you in if you've had certain vaccinations. Has anyone got any views around that?

P11: I think being able to access vaccinations is really interesting, because actually, like you said, people who go to countries need specific vaccinations. Like, I tried to go to summer camp this year, besides the fact that didn't happen, you had to pay loads of money to get the doctor to sign a form saying which vaccinations you've had. And I think if you can have access to that yourself, I wonder if there's a way it could save you that little bit of extra cash to confirm. Because again, like you said, I had to get it printed and then scan it into them, and that makes it very easily to be…fraud…

GD: Yeah, counterfeit.

P11: Counterfeit, that's it. So I think that could be quite useful, and the same with if you have to have certain vaccines to go to other countries, being able to show people on the app would probably be a lot easier.

P07: With the vaccination thing, can't you already on like… I can't remember what it is. I've got like a Patient Access app or whatever it's called, and it shows my medical record but it also shows my vaccination history on it. So I guess isn't that already kind of like a feature that could be…? How would it be different to what is there already with the…like the fact that I can access what vaccines I've had online?

P11: I didn't know that was a thing. But going off that, I think it could be different. I don't know if it's signed off by a GP on that sort of thing. Because usually you kind of need a GP to say, yeah, this is definitely what they've had. I don't know if they've got that sort of permission on that app or whatever already.

P10: To be fair, I thought what this focus group was on was on an app like that. I thought it was on a Patient Access app. I didn't know it was mostly about blockchain sharing and stuff.

GD: I don't know, and Victoria, correct me if I'm wrong here. So the idea will be it will be like a central platform that uses blockchain technology or distributed latest technology, and then these apps would be accessed via the platform. So I guess in the same way that you would access Patient Access off your mobile phone, that's directly linked to patient access, whereas this is much more open than that and it's available to all patients and citizens. And they access or transact with different people, sharing their health data with people they give permission to via the platform, rather than just off one app on its own. Going back to what we were saying originally about health data being stored in lots of different places. Does that make sense, P10?

P10: Yeah.

GD: You don't have to say yes. I'm struggling to get my head round it too.

P10: Yeah.

VN: I think when we first discussed the scenario within the research team, what we were thinking about is, like in the current pandemic, there are lots of localised breakouts, so of course you can't do nationwide lockdowns if there's just one town or city that's being affected at the moment. And I think there was also the idea again, so what can we do to help local governments keep an eye on that, and then also incentivise data sharing? For example, downloading a track and trace app, and then you get an incentive that you can get a voucher, for example. So similar to the other app. But instead of data sharing, you kind of do the community protection sharing.

GD: What about if we did incentivise people to get immunity through an app like this? So when we were first thinking around this, we thought that perhaps people could get paid or have access to, I don't know, local authority services, or a reduction on their council tax bill if they shared their data with the local authority. How would people feel about that?

P07: I think that's very good and helpful. I mean, obviously I don't know if I'm right on this, but if I couldn't just get an antibody test right now, could I? So I think another aspect would be the test to see whether you are immune to it would have to be paid for, because I think you can get it privately at the moment but it costs quite a lot of money. So, say, basically, the local authority will also pay for your test to see whether you're immune or not.

GD: Yeah, there are lots of debates even around whether it's possible to say that someone's immune or not, this is all quite hypothetical really. But yeah, I guess that's an important point as well, about who pays for the test itself.

F6: I think incentivising, in general, is a good idea because everyone always wants something out of it themselves. People like to know they're getting something out of helping other people as well, at the same time.

GD: What if there was some kind of non-financial gain? You know like people can collect stickers or badges, or when people use Strava, they get a rating. If somebody was sharing their health data for good causes and they had some way of…so they could perhaps share that on their Facebook account or Instagram, or what have you, do you think that might incentivise people to share their health data? [No response]. I'm guessing not.

P10: I think that would highly depend on the person.

GD: What about you, for example, P10, would that incentivise you?

P10: I wouldn't. Money all the way. Because I can't go outside and buy a chocolate bar with it, I don't want it.

GD: Fair enough. How about you, P09?

P09: I don't think it would make much difference to me either. That wouldn't really incentivise me at all.

GD: I'm guessing by everyone else's silence you neither.

P11: I think it's a little bit different but I suppose, like, you know how if you sign up to a certain insurance company you get Meerkat movies or something like that. I don't know, something, even though it's not money, you're still getting something out of it. I wonder if that would incentivise people in a different way. I don't know, sharing your health data is a bit more serious than that.

P10: You get access to [voices overlap 0:19:36].

GD: Okay, fair enough. In a minute we're just going to ask you for a little bit of feedback on the focus group itself. But before we bring it to a close, I just wondered if anyone has any final thoughts or ideas around if somebody was to approach you and say, we have this health data sharing platform and it uses blockchain technology, what would your immediate thought be? What would you want to know about it before you invested time in looking into it?

P10: I'd want it to have a better name, because CDIP to me sounds like a virus.

GD: Fair enough. Can you think of one, P10?

P10: No, I can't think of one. I would also quite like to be pointed toward a layman's description of what a blockchain is. Because I was looking on Wikipedia earlier and it was pointless, because what I got out of it was, it was a list, and it was like, mm.

GD: So you would want t know a bit more about the actual technology itself, to trust it?

P10: Yeah, because it does kind of feel a bit like the magic word of technology now, yeah.

GD: Brilliant, thank you, P10, that's really helpful. Anyone else?

P08: I was thinking it depends on what the company is as well, because if a random company just came up to me and was like, we've got this app, I'd kind of be like a bit more cautious of it. Whereas if it was like a company that was in partner with like the NHS or something, I'm more likely to go further with it. It's like on the streets, when people are out with charity workers, you're more likely to allow someone from like Ofsted to approach you than a random charity that you haven't heard of, because then you know it's more legit.

GD: Okay, that's really important, thank you.

P07: I just think the privacy thing, but I do think an app like this would be very beneficial, especially with the blood types. It's just allowing paramedics or anyone in healthcare to do a better job. And it could be quite good for in the future, if there are future viruses and stuff like that, knowing whether you have the immunity, and I think it would help local authorities to be able to make better decisions based on… Say, for example, you've got a spike of cases of something in, say, Manchester, but there's also a high amount of people with immunity, then you've got different policy responses you can do. But I think it is just all about privacy and having control of your own data which would be the only thing that would be concerning. But that's what I'd like to know a lot about before I used an app like that, just to know where… And in a simple form, not just like 300 pages of terms and conditions where nobody reads them anyway, so it's just very clear and short. If that makes sense.

GD: So when you say about privacy, so you want to know where your data is, who's in control of it, or…?

P07: Yeah, where the data is, who's in control of it, and also, like what P08 said, under an organisation that… I think any organisation in partnership with the NHS is something I would trust a lot more, and I think a lot of people would because a lot of people quite trust the NHS. So I think if it's in partnership with an organisation that a lot of people trust, then it's going to be a lot more efficient and effective.

GD: Great, thanks, P07, that's really helpful. Has anyone else got any key information that they would really want to know first before they would even think about using something like this?

P08: I was thinking about another thing. Like people with the salon are using it, could they not overuse their power and just refuse entry to people? Like some people might just… I don't know how to explain it. But could people abuse their power, in the sense of allowing people to come in, and kind of be like, I don't want you coming in? Does that make sense?

GD: Yeah, I hear what you're saying, P08. I think perhaps something like this would stop that from happening because it's something that's completely objective. It's just an app and it says either you're immune or you're not immune. Whereas if you had some other kind of system, it might be more open to abuse. I don't know, that's just a reflection on that.

VN: It's a good comment, because obviously we don't want to give people a system that they can easily abuse towards something like that. Often people make the metaphor that it's similar to trying to enter a club and then have to verify that you are 18 or over. But then in looking at the actual…as an ID that identifies you, just the likelihood that you are actually 18 and over and not putting all your data out there. And then we don't want a bouncer that then goes, even if I can verify, I'm not letting you, because of your issues or whatever.

GD: So the beauty of this kind of technology is that you don't have to share everything, so you can set up ways of, like Victoria's just saying, if you want to prove that you're over 18 but you don't… You know, I don't want to tell someone that I'm 47, but I can prove that I'm over…not that I'd get I'd get ID'd anyway! But if I want to prove… So you could have some kind of mechanism within this to satisfy certain criteria. To say, yes, this person can go into a nightclub, or, yes, this person has immunity, or, yes, this person has blood Group O positive, but you don't know that I've got other sensitive health data that I don't want to share.

Okay, great. Sorry, I'm just seeing that it's already five o'clock. Would you mind, maybe all of you, just telling us one thing that's gone well today or that you've particularly liked about engaging in the discussion, and one thing that would be better next time?

P09: I thought it was quite well organised and it was explained well, because I'm not going to lie, at the beginning I was bit like, I don't understand it. But once you started talking about it properly, it all made sense, if that makes sense, and I was able to have my own opinions.

GD: Great, thanks, P09. When you say when it was all explained, is that more like in terms of having the scenarios?

P09: Yeah, like right at the beginning, I was a bit like, I don't understand the words, but once it was actually talked about by everyone, it made sense to me.

GD: Great, thanks, P09.

P11: I liked how you sent us the scenarios beforehand so that we could have time to think. It's like if you're being interviewed and somebody sends you the questions beforehand, you're kind of not caught off guard and you have time to prep.

GD: Great, okay, thank you.

P10: Yeah, that was especially useful for me when I got confused on the second scenario. I just opened up the link that was leading it, and was still confused, but…

P08: I liked the reassurance of when you rang us to check. Because I was really nervous before then, and you ringing and just running through it quickly and making sure that we were all okay with it was like a really good thing.

GD: Oh good, thanks, P08. What about you, P12, have you got anything you'd like to share? That's fine. P07?

P07: I think breaking it up into scenarios is really good because you can kind of see how it will affect each person. Whereas if you just said, this is what the app is, then kind of what are the benefits of it, it breaks it down so you can see more. Like for different groups what the effect will be, as opposed to just trying to pick out random effects on random people.

GD: Great, thank you. Anyone else got anything they want to feedback?

End of transcript

**File Name(s)**	56418-CDIP-FG3_15-10-2020-Part-1<br>56418_CDIP-FG3_15-10-2020-Part-2
**Total Recording Length**	55 minutes
**Number of Participants**	9 (P13-18)
**Final Word Count**	8,236
**Total Number of Pages**	18
**Date Completed**	28 October 2020

[56418_CDIP-FG3_15-10-2020-Part-1]

GD: …in this platform, what you think might be, you know, the major benefits and drawbacks of using something like this are.

P18: Well obviously, just to make a start, if people that are treating you can see what's wrong with you, what's already happened, what medication you're taking, your medical history and so forth, then that allows them to offer you better treatment. So that's an obvious benefit to the patient. There's also…I'm assuming that the data is in some way anonymised, so when it's passed through to organisations that are not directly regarded with your health, so pharmaceutical organisations, people like that, that they get different sets of data than my physician would get under the same circumstances.

GD: Yeah, that's a key thing, P18:, is that it would put you in control of deciding who is able to access which bits of data.

P18: How much time, though, would you spend involved with that. I mean, these things can become a little…I can't think of the word, but you can get complacent with them. And if somebody's asking you on a regular basis for information, you're not necessarily certain who that's coming from and how much guarantee is there that the pharmaceutical organisations are not then moving that information sideways. Once they've got the data, can they download it, can they store it, can they use it for other things?

GD: So it sounds like you're saying that you would want some kind of assurances from whoever you send your data…from whoever you're transacting with on the platform, you'd want to have a very maybe simple way of knowing exactly what they are able to use that data for in the first instance, is that right?

P18: Absolutely, yeah.

GD: Yeah.

P16: Can I ask then, does this platform…it brings in, for example, the sort of health record that's created when you go to your GP surgery and ties that in with if you then go to a hospital appointment and they're putting information on your record at hospital, so then brings in any information you might put into your Fitbit, for example, that draws that all together in one place, does it, is that correct?

GD: At the moment, it's all up for discussion because this is…we're at the very exploratory phase in the research. So first of all, we want to know what people would like to do and what kinds of features and functions would be helpful to people. So from what you're saying, P16, it sounds like you would like that sort of meeting up of all the different records so that it can be shared.

P16: Well, I suppose the thing is, I guess what you don't really want to do is create another kind of separate entity where it's storing something else and doesn't tie all these things together. Because from what I understand at the moment, you know, you go to the GP and there's a record there, you go to the hospital and there's a different record there, and the two things don't seem to know what information's in either of those. And then you might use a Fitbit and that stores something else, I don't know whatever other sort of medical data collections things people might use.

And that was something else that none of the things talk to each other. So I suppose I wouldn't see the…I didn't see a benefit of using another kind of way of storing more data that is separate to all these other things, as opposed to one of the benefits I guess should be to tie those all together somehow so that the individual can access these things from one place, and so can a health professional wherever that health professional might work, I guess it's obviously trying to achieve. Is it?

GD: Yeah. Well, one of the key things is that none of the data would actually be stored on the platform itself and the platform is more of an area where a patient would have the ability to give permission for different professionals or health researchers or pharmaceutical companies to access specific bits of data. So this is more of a mechanism really that a patient would use to say…say they were contacted by a pharmaceutical company and they say we're doing some research into diabetes, you know, are you happy to share your data. And this platform will be a way of corresponding with them to enable them to eventually access that data. But the actual data itself is not situated on the platform.

P16: Right, okay.

GD: Does that help?

P16: Yeah, I think so. I'd have to…I'm trying to get my head around how that would kind of fit in with everything if you like.

VN: Just to intercept maybe, sorry, just to follow up. So in this current scenario, we would have been trying to have a platform that kind of provides an overview of different data sources and whether data access has been made, and to have like a record of where is your data stored and who has access to it. So kind of like drawing it altogether but not as a data storage themselves but just as an overview.

MH: Yeah, so it becomes essentially like an aggregation layer that you need to kind of encourage data providers to engage with this type of platform and this type of ecosystem that you would be able to set up around the technology. So it's more around aggregation and how you bring these different data sources together, but also providing patients with more control in terms of where that data gets shared to beyond the platform.

P14: So am I right in thinking, if you had…say you had multiple health conditions, so with diabetes I have a Freestyle on my phone, but say I had diabetes and asthma and COPD, and I had similar technology, could I pick and choose who I shared my information with? So for example, my diabetes specialist I'd share my Libre information, my asthma I just want to share my asthma information. Is that what you're saying?

GD: Yeah. At the moment, it's all up for discussion and it's all around what people would want too. I guess the central idea is you having that [voices overlap 0:07:00].

P14: Patient control, yeah.

GD: Yeah, over what you share with who, and what people think about that. Because a health professional might have a different view and think well, actually I need to know what's happening with your diabetes while I'm treating your asthma.

P18: Yeah, well, there's a couple of issues there that crop up. Firstly, as far as I'm aware, GPs should hold most, if not all of the medical information that they have about you. So hospitals treat you and send information to the GP, which is stored in your file. So theoretically, the GP should have everything. The problem with that is that GPs have that data in such different ways, lots of them still have paper-based records that never would get uploaded. So that information is never going to be available to you unless we can get that uploaded.

The other thing is what you're discussing is what I want. So from a…if I'm seeing a health professional, I want them to know as much as they possibly can about me because then they can make their informed decision. So I'm not going to hold anything back. However, yeah, if I'm dealing with a pharmaceutical company or any other individuals interested in for this for their own purposes, then that's a different story. So you'd want the two stages of that really.

P16: Yeah, I agree with that, a health professional I'd be much more inclined to share things with than, as opposed to the sort of commercial organisation that, yes, on one hand might be looking to, you know, improve…come up with a new drug or something like that, but obviously they've got an ulterior motive as well. Well, I think they have, maybe they haven't.

MH: How do you feel, like, if you take the NHS as an example obviously that, you know, a lot of their services now are actually outsourced to corporate organisations such as like merging care, for example, how…obviously, with this type of platform you could potentially set some very fine grain conditions around, you know, you may only share certain bits of data with the NHS, but if they then try and share that with Virgin Care, you could actually put a stop on that. But then potentially there's a lot of impacts then or implications of not sharing that data.

P18: Well, you're right, yeah, if you're moving outside of the NHS into sort of paid for private healthcare, then that becomes a different [inaudible 0:09:30].

P13 Would you be able to put like a temporary sharing thing on it, so like you could share it, say you were having a knee operation and that was outsourced to a private hospital and that treatment was going to take three months from start to end, including the physio, would you be able to say I want to share my information for this amount of time and then put a stop on that?

GD: Potentially, yeah, that's a really, you know, important point and it has come up again in another focus group that people are interested in having that really fine grain control, not only with who they share it with but for how long.

P13 Yeah [my mum 0:10:12] had a knee op and it was done private, like, hospital, paid for by the NHS. And she had…like, all her stuff was dealt with at that hospital and they obviously had access to only certain things. So it would be interesting if you could say I want to share everything but only between these dates.

P15: Can I just go back to a basic question…

GD: Yeah.

P15: …about security. Because when this was first mooted quite some time ago I think, people said…I didn't say this, that they were worried about being traced or people looking for them, you know, no matter how obscure the material was, someone would find a way to access their medical records and other bits of data about them. The overriding thing is that a thing called the open door syndrome with security systems that no matter how secure the security system is, someone's going to leave a door open somewhere and someone's going to find a way into the secure process or whatever it is that you're trying to protect. So I think that's where most people are at fundamentally in the sense of security, they want that overriding confidence that whatever they engage in is going to be protected.

GD: Do you have any ideas around what would make you feel more secure about that?

P15: I don't know, I mean, people don't trust politicians and presumably they don't trust pharmaceutical companies to a large extent. And to a varying degree, I mean, criticising the health service is like criticising your parents, most people don't like doing it. But people do complain, you know, so you've got to have an open view about this, I think.

GD: That's a really important point. Thanks, P15, for raising that. I'm wondering how everyone else feels around what kind of assurances would people want in terms of the security?

P16: I don't know, I think some people are always going to be sceptical about it and some people are more relaxed about it. Like, I'll always be on the sceptical side of things, so no matter what Facebook say about your data's secure, I'll never put anything too personal on there because so many times years down the line, there'll be a breach of security. But then other people aren't bothered. So I don't know, I think there's always going to be some people aren't bothered and some people are bothered, regardless of, you know, what assurances do you give them. My opinion, but I don't know what other people…

P14: I think…

GD: Sorry, go on.

P14: Sorry, I was going to say I think if there's a way that…I'm not technical, but there's a way that your data's held, so say all your health conditions, past operations, whatever it is, but it's kind of like de-coded so your name and your NHS number are not…there's some way to make them not attached. I don't know how easy that is. But like at the surgery I work in, certain things…there's certain security processes on the system, SystmOne, so there are ways to protect patient information. So there needs to be something to make sure that it's not connected to you, but I don't know how easy or hard that would be, if it can be done, I don't know.

P18: There's some technical issues there. I mean, anonymising data like that is something that's done I think quite regularly for research purposes. So, you know, organisations collect large amounts of data about a particular area and then they don't know who's involved or what. All they know is patient X did this and patient Y had that.

P14: Yeah.

P18: I think part of the problem is it's a security issue is an issue. The NHS aren't renowned for their technological abilities, which is not where their strength is, is it, you know, the doctors and nurses. So if this data's left open on a screen, can it be collected by somebody else, can information be made available that shouldn't be made available by mistake, which is I think where P15's aiming, someone goes on a screen, gets information, downloads it, it's on there, it's already available, someone comes along with a pen drive and says, oh, I can use that and takes it away.

GD: I think when we come to look at the second scenario, we focus on this a bit more, but as you've brought it up into conversation. One of the things about this type of technology is that, unlike the NHS which is one big organisation, they are the controller of the data and it's all held in one place. And that does make it a bit more susceptible to data breaches and hacking. Whereas the idea of a blockchain type technology, I don't want to get too much into the really technical side of it, maybe might…Victoria you want to chip in, but the idea around blockchain technology is that it's distributed. So that kind

of risk is shared across lots and lots of different points, rather than actually at one point which is the case at the moment with the NHS.

VN: Also, so there's been a few points been made which are very important about security. I just want to clarify that with a system like this data donation platform, because the data is not stored on the actual platform, we don't really have a way of making sure that the databases or the systems where the data is held are secure. But what we can do with this is, like, if data has been taken in or out, then we can track it. And then, so if there has been some movement of this data which seems suspicious or doesn't seem to be either involved in direct care or according to your preferences, it can be, like, highlighted. So there could potentially be people regularly checking if the data that has been moved around is actually…that this has actually been legal and in a way that has been according to what the patients want. If that makes sense.

P17: Yeah, I'd like to ask a question. You say we can control it. Who are we? When you said we can control the information that goes out onto the various platforms, who can control it? Because you could end up with thousands and thousands of platforms with all different interested parties trying to get information through the patients who give them permission, okay, but this is going to be a massive task. Who is actually going to control what data is going out, whether it's been treated properly, whether it's been anonymised, whether or not it's been deleted in due course? Who's going to do that? It seems like a job for MI5.

MH: I can give some insight into that. So you heard this kind of concept about smart contracts where you can essentially establish rules which is written in code, which are verified on this kind of distributed database essentially. And so in theory, the idea is that you shift some of the control away from these kinds of centralised institutions where, at the minute, you know, you might have just the NHS managing your health records. And actually you're shifting control to yourself where you can define the rules that are embedded within these contracts that then essentially manage how the data is shared. So there's this idea that you're actually shifting kind of the [locus 0:19:15] of control away from centralised companies and institutions, and more into code and rules which actually the patients define.

P15: The only trouble I've got with some of this is that technical authors who write instructions don't write instructions for ordinary people, they write instructions for other technical authors. So, I mean, I've got a TV, it's got about 200 different functions and I don't know what they mean, and a lot of the explanation of it is difficult to follow. And it's as much about a [concept 0:19:58] as the information as well because I don't know what some of the functions can actually do. And whether they're using it or not is another completely different question.

But that's going to be one of the troubles with this is conveying the actual information in a format that people…normal, not ordinary people can

understand. So you're talking about coding and things like that, a lot of people just won't relate to that at all, I don't think.

GD: That's really important, P15, thanks for bringing that up. And this is exactly why we're doing these discussions early on to find out how to explain maybe something that's an alien technological concept to most people in a way that people can understand it enough to want to use it. So it sounds like you're saying that, you know, you want some really straightforward explanation of how it works, is that right?

P15: Not so much straightforward. If you went back 40 years and you gave someone a video recorder and told them you can record programmes off the TV, right, most people learnt off other people, they didn't learn off the instructions or what they were told. But the actual concept was difficult to grasp at first. Do you understand what I mean?

GD: Yeah.

P15: If you've never seen a white swan or a black swan and people are talking about it, you can't envisage something you haven't got a concept about. Do you understand what I mean? You're inside the tent shouting out, the people outside the tent don't know what you're shouting about. Do you understand what I mean?

GD: Yeah, I get you, P15. I'm just wondering what other people think around that. So quite a lot of people use say, for example, internet banking and they don't really understand how that actually works but they still go ahead and use it and are able to kind of make transactions. From your perspective as patients and members of the general public, what do you think is important for people to know before they can engage with this idea?

P18: I think they need to be told how they're going to benefit, firstly. I mean, in order to get people to release this information, which the very first line on your presentation was that this data is valuable, not just to us as individuals but it's valuable to the NHS, to medical organisations and to private companies. Insurance companies would love to get hold of this information and they could be all sorts of interesting reasons. So your first question, how do I benefit from you doing this, what's my benefit. And then from there, we can see whether or not it's worth my while. I benefit from internet banking because I don't have to go out, I can do it from here, my bills are paid and kids get all the money, and there you go.

P17: Well, I'd like to say that in this particular instance with P18:, to me the story unfolds in a way that I think most people would approve that. You know, I looked at…having read his scenario, he's got a specific illness, it's in a controlled…the study is controlled, he forms a contract with the platform, whoever it is, the liver people. He registers that it's all okay for him to be contacted and he's assured that it's a secure platform. And so it's to his personal benefit, the whole thing is to his benefit. And it implies, although it

doesn't say so, it implies that the only records that are going to be divulged are the ones that he approves.

The problem is, right, that having done all that, once he's onto the platform, they can ask him any questions that might be outside that personal…those particular details about his liver or whatever. I mean, they could be asking…there's several people on here who suffer from diabetes, they might be asking about whether or not he's got diabetes and whether that's affected his liver.

And that…slowly but surely, especially if you're involved in several platforms, it might become a general thing, before long you're just giving all that information out there. And although a company might guarantee to give security guarantees, there's no such thing as a company really, there's no such thing as the NHS, they're only people that work within it. And a chain is only as strong as its weakest link. So if you get a person within any of those organisations who's offered half a million pounds to give a load of information about people who suffer from diabetes, trust me, they will provide that information and provide it for a lot less than half a million pounds, they'll provide it for ten thousand pounds.

So unfortunately, data security is weak, is going to be weak. But, you know, if we're going to move forwards in terms of health, this has obviously got so many benefits, that's the way to go. But we can't pretend that by putting in security levels it's going to protect that data. It won't protect it. It can't protect it. Look at the US military information, even that was stolen. Information will get stolen. That's my opinion.

GD: Thank you, P17. You summarised a lot of the benefits and features of this particular data donation platform really well there. I'm conscious of the time. We've been thinking around, you know, everyone is different and has different perspectives, which is why we really want to talk to you today and to find out yours. We've also got a couple of other perspectives we'd just like to show you that are from people in different roles and might use this in different ways or have different kinds of concerns.

So we just want to quickly show you these perspectives and ask you to think about…just choose one that really speaks to you or that you think is interesting or raises maybe a concern that you share or that you oppose. So myself and Victoria will just show you a couple of these perspectives and see what you think.

So we've got Rose here. She's got concerns about an app developer possibly selling her data then onto other companies, once she's corresponded with them outside of the platform, without asking her permission.

And there's also May. She's thinking around, well, can they say they're going to anonymise my data but then, you know, reveal my identity to the government, for example, without actually asking her.

There's Pavel. He's thinking about, well, okay, so there's all these organisations interacting on the platform, how do I know they are who they say they are, how do I know they're genuine.

And then there's Jonas. He's got three children and he's thinking about, you know, he's got to manage all their data as well and what about the responsibility of making those decisions on their behalf. What if, you know, some data is wrong and needs to be corrected, or they want to have their data deleted, how would that affect them.

And then Michelle. She works for a pharmaceutical company and she's explaining that it takes a long time and is very expensive to try and get enough patients together to do a clinical trial, which delays the process and can possibly make some research just not happen because it's not viable. So she's explaining that it would really help research, medical research if they could access the right kind of patients quickly in one place. And also, that they're happy to pay, so they might be happy to pay for that data, either to the individual patient themselves or perhaps make a donation to charity.

And then finally, we've got Mahmood. So he's a lawyer and he's got some concerns around consent. So he's thinking, well, if there aren't any other witnesses like a healthcare professional, how can we be sure that that person has actually consented to share their data or they weren't drunk or something.

So we'll just bring up a little summary of these different perspectives. And I just wondered if you wouldn't mind, all of you, just choosing one that you'd like to perhaps highlight or you've got a view on.

P17: Have we actually seen these? I haven't seen this, I've only seen the two scenarios.

GD: Yeah, this is new information. We didn't send these in advance because we were interested in what your perspectives were first, without thinking about what somebody else might say or think.

P16: I mean, I think they're all really quite interesting actually and sort of almost sum up the kind of things we've been talking about. But the one that stood out to me was the one from the pharmaceutical company which rang alarm bells, to be honest. If they're talking about paying money for data, then that makes me think about people, you know, feeling under pressure financially and thinking, well, I'll give up my data to these people in exchange for money. Which is more worrying, I think, you know.

But, I mean, one thing that I thought with the benefit from the P18: scenario you presented was the fact that he found out about a research trial that he may not have been able…may not have found out about and they found a participant that they may not have identified, other than through using this platform. So yeah.

GD: Thanks P16.

P15: Form a risk assessment for how can I be sure that organisations that have access to my data are genuine. If there was like a tick box risk assessment thing I could access which was simple, I could look at it and I could weigh the questions up beforehand myself, and if it was in a relatable form to my normal life. Because the way that's been structured, it's not going to be something I will be able to understand quickly, will it, it's a kind of professional perspective to look at it in this kind of way.

GD: There's a few different things that we could do around that. How would you feel if maybe another organisation kind of oversaw the platform, so they would either allow access or not for companies to use the platform, so they would check that in advance, how would you feel about that?

P15: There'd have to be a code of practice, wouldn't there, with someone in charge who you could trust. At the end of the day, who was either going to be prosecuted or lose their job, or they're going to be in some kind of jeopardy if things go wrong. Like the Health and Safety Executive or somebody who have got responsibility for overseeing workplace health and safety. So the way I'd look at things is most people know about risk assessments and that's the sort of way a normal person would think about things nowadays, I would think anyway.

GD: Brilliant, yeah. Okay, so how about if the people that use the platform were able to display in some way their experience? For example, on TripAdvisor you've got people that put up reviews that say, you know, I donated my data to this charity and this has happened and I had a positive experience, or something like that. How would you feel about something like that?

P15: You could have a kitemark system I would have thought. The Which report is a bad example I think, or Trustpilot or something like that, because some people just don't trust them as well. So I don't know who you could trust, the House of Lords [inaudible 0:33:40] trust them, you know. So I really don't know. I suppose if someone hasn't got a financial stake in the situation, then they're going to be exonerated if something goes wrong because they're not going to get any pecuniary advantage, are they? That's one thing.

GD: Thanks P15. Has anyone else got anything to add to that or a different perspective they want to look at?

P17: I'd like to ask a question. Does an individual patient always…is that patient always asked personally, directly, for the information? Or does he or she sometimes just give permission for some information to go directly to the platform user? In other words, could your GP say, listen, the British Liver Trust are carrying out some exploratory work, would you mind me giving them your information. Because if that was the case, then that patient wouldn't actually have control of the data, would he or she? I'm just asking,

is that what could happen or does it always…is the patient always contacted by the platform?

GD: The idea for this would be that it's the patient themselves that would make that decision and that would set that level…different levels of consent. There is an opportunity maybe for someone that's really interested in research that they could potentially say I'm happy to share all of my data with any NHS approved research organisations or any universities but not pharmaceutical companies. Or I'm happy to share my data with anybody if it helps [find 0:35:35] a cause. So it enables people to decide for themselves, you know, how little or how open they want that.

P17: Well, if that's the case, I would suggest that it's even more critical that a patient has the ability to be able to see his or her own records. Because although in theory we can gain access to our own records, in practice we can't, it doesn't happen. And maybe that's because what P18: said, you know a lot of stuff is kept on paper records, perhaps it's not as sophisticated as I tend to think it is. But, you know, I think before patients are going to be prepared to have their information shared, they want to know what information is on their records.

GD: Absolutely. Yeah, that's a really good point.

P18: There are practices that do allow patients to see all of the data that's stored. If they've organised it correctly, then you can have access to your data. I know of a couple of people who I've worked in the past and they've been with a GP practice that allows them to see everything, every line that's written about them is available to them, so all the medications, any treatments, et cetera. And that's useful for them. But yeah, most people, you can see what you can get as a repeat prescription and you can make an appointment, can't you. So electronically, it's very little control.

And yeah, this would be a boon to that if this allows you then to access your data as an individual and make sure a) that that data's correct and up-to-date and a true reflection of what should be there, that would be important. And equally, then if you've got control over who can see it and what they can see, when they can see it, how long they can see it for, then all those things will be beneficial, not just to you as an individual but also to other organisations.

GD: Thanks P18:.

P13 My old GP, I could see absolutely everything. And then I had to move back home after uni and obviously register with a different GP, they literally don't let you see anything. It's the bane of my life as someone with multiple, like, stuff going on. So yeah, definitely the idea that I could access and see everything without having to battle with the GP constantly because they don't think I should have access to my own data, would be very helpful. So I'm definitely on board, like, if it meant I could access my own data even just for that use.

MH: Why do you think that is with your new GP, is it more that they don't want the administrative kind of burden?

P13 I have absolutely no idea. My old GP was the uni health service, so they're obviously very hot on stuff like that and new stuff coming through. With my new GP, I can't get a sensible answer out of them.

P18: There is definitely a cost issue isn't there, GP surgeries have to pay to have that data stored, updated, corrected, maintained. And they're not willing to pay people to do that.

P13 It's even just having blood test results though, like, it's an impossible rigmarole, especially at the minute, having to go through just to get hold of, like, some blood test results.

GD: Yeah, that's really important, P13, thank you. P16, were you trying to say something?

P16: Well, yeah. I mean, it was partly that I think it is a really good idea for the patient, an individual to be able to access their own records in one central way and tying up, you know, primary care records with, like, hospital records and all of that kind of thing in one place. I mean, I've got no idea how I'd access my hospital records. But also, one thing…I have looked at my GP record recently and it's very patchy anyway. But one thing that struck me is what happens if you don't necessarily agree with what has been written in that record. I understand obviously that would have to be dealt with, with the person who put it in, but then if I'm then sharing that with a third party and they're looking at information that I don't necessarily agree with or perhaps it's been…maybe it's an error or it's maybe just an interpretation and the professional interpretation is different to my interpretation, and I'm sharing it with a third party. Then, you know, that sort of…the quality of that information in the record is obviously vitally important for that then to be shared with someone else or, you know, a third party.

GD: These are all really important and very valid points, and slightly outside of the platform really in terms of your relationship with your own general practitioner and your own record. What this platform does is it enables people to see a permanent record to be there of who has access to your data. So if you have allowed somebody, a healthcare professional or a researcher or a company to access a particular bit of data, your permission is stored there permanently and you can prove that. And likewise, you know, if somebody's tried to access something that you said that they couldn't, it's very, very difficult within this kind of technology to change anything. So although it doesn't speak directly to the issue of having perhaps incorrect health records, it does highlight one of the sort of benefits of this in that it is a permanent record, so it's very good for like an audit sort of purpose.

I'm just looking at the time. Do people want to just have a really quick five-minute break before we…

[56418_CDIP-FG3_15-10-20-Part-2]

GD: So because of the time, either maybe from your own perspective or something from one of the other stakeholder perspectives, if maybe everyone could just say one thing about this particular scenario, either what particularly appeals to them or concerns them about it.

P18: [Inaudible 0:00:18] have to see the benefits from the point of view of the hairdresser, the café owner, obviously they can get to work and they can guarantee that they're free for any customer coming in. I'm not sure about the customers wanting to download an app to say that, okay, I'm free as well. I mean, how important is a haircut? From the lady in bed, yeah, obviously that's a different scenario altogether.

P16: Yeah, I suppose…I mean, I can see the sort of benefits of being able, in this scenario, to kind of self-certify that you're immune, but does it mean that for it to be…for it to work, would everybody have to also do the same thing you're coming into contact with. Because then, obviously that is not just one person doing it, it's every single customer who comes in or every person who…I suppose it's a bit like the contact tracing app isn't it, I guess it only works really if everybody's doing it, if I understand it correctly.

P14: And I agree with the lady that's been, you know, sharing data with other healthcare professionals but she wants to pick what data she can share. So I've had…I've got five health conditions that I've had since I was a child and I want to share some information with some healthcare professionals and some with others, maybe not all of it. So I think it's good that a patient can have the choice.

P17: In this particular scenario, you can see her holding a telephone and presumably a portrait…her photograph is on it. And then there's a barcode isn't there? Is that a barcode?

VN: It's a [inaudible 0:02:18].

P17: Yeah, whatever it's called, that little square thing that you [voices overlap 0:02:22].

GD: A QR code.

VN: QR code.

P17: Yeah. So, I mean, in this practical situation of a ladies' hairdressers, what's that saying to anybody? I'm just asking the question. I can see that the picture of herself is identifying her as being herself, but what's the barcode doing? Does that mean to say that the hairdresser has to have some equipment in the shop that can read that barcode?

VN: That's a good question. So once it's set up, so you only have like a picture or issuing the identity once. So once you have set that up, you can have just a barcode without any information about the person itself on it. So then if you meet another person who also has a smartphone and the app, they could scan this and then verify that this has been…they have been in contact. But without the kind of like track and trace but removing the surveillance part of it. So it's not a person's identification that's been changed but only the kind of barcode and the number that's issued with it. So you don't have to worry about the information being shared with lots of people that you meet, or if you go to a certain space where they kind of require that. Yeah.

P17: I didn't understand what it was, but I understand now, thank you.

P15: P15. I've got to go shortly. I just find this episode a bit overwhelming information wise, trying to look at all these screens and what have you, and trying to put it in perspective. I've got a dyslexia statement but that's not really important but just the way this has been presented is a bit much. I'll have to go back to this again and look through it all again to make sense of it, quite frankly. If it was presented in a different way, I think I could relate to it a bit better. As I say, I'm going to go now anyway. Okay.

GD: Thank you, P15. That's a really important point, thanks for raising that with us. It's quite hard when we're doing it all virtually. In an ideal world, we'd have something a lot more interactive and be face-to-face and be able to do this slightly differently. But that's a really important thing for us to take account of, so thanks for raising that. Thank you.

VN: Thanks P15.

P15: Right, okay. See you then.

MH: Thanks.

P17: Well, again, this is quite a specific app isn't it and I can't see many people objecting to something as straightforward and useful as that.

GD: What about the capacity…so taking the immunity side of it out for a moment, although that's a very pertinent subject to everyone in the current times. What about being able to say, for example, have an emergency feature so that if you wanted to share your blood type or anything about your condition with a paramedic, for example, if there was like an override feature on this, how would people feel about that?

P13 I think it's a good idea. I mean, I have all of my information, because I've got an iPhone there's like a feature on that where, in an emergency, you can just access any information you want to store. So for me, I've put that I'm diabetic and I've put emergency contact and medication I'm on, and I have a pump and a Libre and all of that, so they know what they're dealing

with. So I struggle to see where that's any different and it sounds like it would be harder to access than just like the lock screen, hit the emergency tab and then it comes up. I don't know how you could get much easier than that, so I don't know. That's purely an iPhone thing though I think.

GD: Yeah, I think that's the only way it might potentially be different is that this will be across the board, regardless of what kind of phone you have, you might have the capacity to say. So when we were talking before about consent and different types of control, on the flipside of that there may be occasions where, you know, under normal circumstances you don't want to share your data, but if you could have another option to say in this particular instance, so in an emergency, I'm happy to share all of my data. I just wondered how that might sit with people.

P16: I don't know, again, for me, I feel like I would be uncomfortable going into a hairdressers and showing them something that they can see, you know, I find that very kind of intrusive. But I can obviously understand if you're at, you know, death's door, if you like, and you're rucking up at hospital and they need to know your blood type. And obviously, they can't get on your health record, which they should be able to, you would hope, because it's all stored electronically but it sounds like they can't.

So obviously, I can see in that situation the benefit of it. But I would feel very reluctant to casually show it to my hairdresser, it feels like, oh, I've been approved to continue with my life, do you know what I mean, it seems a bit intrusive. It's kind of like, I don't know, branding people almost, do you know what I mean, it feels like you're branding people they're safe, they can do things, this person can't do things, this person can do things. I don't know.

GD: Thanks P16. I'm really wary of the time. I just wondered before we close if anyone has any particular questions or ideas around this, how this left people feeling about the potential of having this kind of level of control over access to health data.

P13 I like the potential from a sharing between healthcare settings. I really like that because [inaudible 0:08:44], like when I was at uni, how I was seen both at my uni clinic, at my home clinic, and they didn't share anything with each other. So I was responsible for carrying that data backwards and forwards. And having a way to do that digitally would have been amazing. Less enthusiastic about the private company side of it.

GD: In what way, that they could ask you for data?

P13 Just like the bigger it gets, obviously the more likely there's a risk with things, I guess. So like the more people that you share the data with, someone else raised it earlier, like, eventually you might get to the point where you're just sharing everything anyway.

GD: Okay. Thank you, P13.

P16: I think that sums up…I would totally agree with what P13 just said really.

P14: Yeah. And I agree as well, the sharing…it's so ridiculous that the different hospitals and different GPs can't communicate. We're in 2020. So that would be amazing for people with long term conditions. But I disagree about the pharmaceutical companies. I think as long as you can select that you just want to help with research, for example, I think that's a brilliant thing. But it is a bit of wariness of if they get different information. As long as it's secure and you can select exactly what you want them to know, I think that could be really helpful to finding treatments and possibly cures for different conditions.

GD: Great. Thank you P14

P18: I think we have sort of a built-in scepticism about some of these things. I mean, it's very difficult to understand the procedures involved. When you're talking about this blockchain technology and distributed [inaudible 0:10:28] technology and it's very difficult to understand, in terms of how it's actually managed. And in fact if I'm aware correctly, it's not managed, it manages itself. So it's set up so as that it looks after itself. If someone puts incorrect information in, it throws it out, if someone puts the correct information, it collects it.

And it's really complex stuff. So for most people, it's going to be, I don't get it, how…I've got this data, you want my data, can I let you have it or not, is it going to be beneficial to me, is it going to be beneficial to my kids. Doing that research that then solves a problem that means that my kids don't suffer what I've suffered, then that's great. But, you know, how do I choose.

GD: It's the really big paradox really or problem that we're grappling with right at the very beginning of this project, in that how much information do people want to know. Because like you say, blockchain technology is quite difficult for anybody with no technological experience, such as myself, to understand and get their head around. Is it more important for people to say, look, these are the benefits, this is what you can do and it uses this kind of technology called blockchain but you don't really need to know about that. Or do people think, well actually, no, if I'm using a platform like this, I want to absolutely understand how this works. So thanks for raising that, P18:.

P17, have you got any final comments?

P17: Yeah, I think there are many benefits for these platforms, research and general health improvement. I've probably stressed, I feel there's a lot of potential for harm as well. And I was just thinking about…I was out this morning and went to have a look at some bikes. And you can buy a chain or you can buy a very…you can buy a strong chain or you can buy a very strong chain to prevent the bike being stolen. But I suppose if you gave

everybody 25 years prison if they stole a bike, it wouldn't get stolen, would it.

So when you're putting all these restrictions on people or on the platform themselves, they've got to have proper punitive fines or punitive things that will happen to them if they breach the data. Because we know of a lot of scenarios where there are supposed to be restrictions in place to protect information, like with journalists and all the rest of it, papers. But, you know, when the fines or the punishments are minor, it doesn't mean anything. So I would say that that's an important part of this for the platforms themselves.

GD: Right. Oh, that's really helpful. Thank you, P17.

End of transcript

**File Name(s)**	56782_CDIP_FG_27-10-2020_Part1<br>56782_CDIP_FG_27-10-2020_Part2
**Total Recording Length**	80 minutes
**Number of Participants**	7 (P26-P30) VN/GD/MH
**Final Word Count**	10,946
**Total Number of Pages**	23
**Date Completed**	11 November 2020

[56782_CDIP_FG_27-10-2020_Part1]

GD: …perhaps the benefit of using something like this.

P28: Well, I guess for me, the major benefit is the emphasis on patient control there. I'm both a professional but also a user of a similar kind of app because I have the FreeStyle Libre, you know, the diabetes, where you feel like…well, you are constantly tracked. Particularly since I've got a new phone where you used to have choice over when you uploaded it, the data, now it seems to me that everything is uploaded. And I haven't as yet, I've only had this latest phone within the last couple of weeks and I haven't seen anywhere where I choose which bits…who sees which bits. So for me, that issue of patient control is really important. Not only that but because of the potential…and it's like, I'll go on Facebook and I'll see FreeStyle Libre pop up on it. So it's where this information is shared across these platforms. It's a minefield, I think.

P29: Definitely. I've not used that…I mean, I've been diabetic for many years now and actually I've managed my condition very well just using stuff myself, just putting stuff into a spreadsheet and that kind of thing has been perfectly acceptable for me. But then I'm lucky that I have a scientific background and I understand my condition and I can actually manage it very well.

One of the concerns I think P26 made a point about this, you know, actually there's a lot of people out there that don't have the knowledge to do that. That's my concern, you know, they will actually be either just using it happily and not even considering what data they're giving away, or they just won't be able to do this sort of thing. The other concern is, of course, there's older people that won't have this technology. So you're limiting it almost by definition, you're limiting it to younger people. And that's a major concern to me.

P26: The only thing about that, P29, is the young are very good at showing their parents and their grandparents. My friend isn't very good with IT but her daughter is a whiz with it. So I think they can be supporting the family. Although I do agree with you, you know, the health literacy and things like memory and cognitive impairment, all of those things are going to come into play with this.

P29: But they may not have the technology, that's the point. My parents would never even…even though I had a computer then, my parents would never even have considered having one. Likewise, with my in-laws, you know, they're really…even though you could show them, they just don't have the technology to do that. They've got a very basic phone.

P30: I think that's a very well-designed app, as other people have pointed out, that gives the patient so much control over which data they can share. And I am generally in favour of sharing data. I am aware of certain platforms that could really be integrated into the NHS and perhaps I am in favour of a

model where patients feed back as much as they can to assist doctors and also, prevent developing certain diseases, so earlier recognition. So I think this system, this app is excellent, some of the features that you can see the history of information exchange and you have so much control over consent, it's excellent.

GD: Great, thanks, P30. I noticed, P26, you mentioned a couple of things in the chat, I just wanted to bring those in for everyone to discuss. Is that alright with you, P26?

P26: Yeah, fine.

GD: Yeah. So P26 was wondering about issues regarding health literacy. She says that she has an illness in the same family as RA, so it would be great.

P26: Yeah, it would be absolutely fine. I've got to say for me, I've got limited IT skills but I think enough to be able to, you know, manage this. But if I can just give a brief scenario. My friend with rheumatoid arthritis decided to stop taking her meds because she was putting on weight. She didn't tell the doctor, she just did it off her own accord. And she had a stroke. So then her health was in crisis, she went into hospital, she did survive the stroke. But I'm just thinking of those kinds of adverse events that can happen to anybody. And at that point, you would be in control of the app, you know, and then it would need updating. And I just wondered about that side of it. I don't want to be too negative because I think this is a great thing.

GD: Yeah. No, it's fine. I mean, all the negatives are good at this point because this is where we've got a chance to kind of try and change the design or address people's needs or concerns, which is why we're having these conversations before anything gets built. And in terms of the crisis situation, when we move onto our next scenario, we've got another idea for a health certificate. And within that, we were going to talk about things like…they call it break glass technology, is that right, where there can be like an override so if somebody is in crisis and they have specific health data they need to share with healthcare professionals, maybe there's an opportunity around that to override consent preferences. How do people feel about that? If we move on a little bit to the kind of control that you'd want to have over how this data is shared.

P30: Can I just add something?

GD: Yeah, sure.

P30: I would like to see one feature added to that, the ability to upload documents by patients. Because I've done some reading and I've started doing certain things like I've reflected a lot on my past about…I'm suffering from a number of autoimmune diseases and these diseases take years to develop. So by reflecting on my past, I've noticed some things that happened even when I was a child that could potentially be start of these diseases. And I've compiled a file with all major health events, so all the diseases that I had,

all the medications that I took. And also, later on, I did some extra tests, I had some extra tests done on myself like DNA testing, tests for allergies, et cetera. And I would like to be able to feed that back to doctors, to supplement what they have on record about me.

GD: Okay. Well, that's a really useful suggestion, P30. So is there [voices overlap 0:08:24] way that you can add data, rather than [voices overlap 0:08:29].

P30: I don't know if anyone heard about the Living Matrix app. It's a platform, it's sort of a database where you compile a list of all the health problems of all the symptoms you've experienced, you describe how long have you had them, the severity, et cetera. So it builds like a whole profile, health profile of you. And I think it could be very useful to doctors and healthcare professionals to see what has been happening with you.

GD: Right, yeah. One of the ideas with this particular platform is that it's a mechanism really for people to choose how data is shared and what data is shared, although the data itself is not held on the platform. So potentially, it could be linked up to something like the Living Matrix or a particular app like we've just spoken about, the [Ramora 0:09:40] app.

I've just noticed that P29 and P26 were talking about the integrity of the data, that's a really interesting point. Does anyone have anything they want to say about that, so how can we trust people to input the right data?

P29: It's not…yeah, I mean, it's an interesting question, I've actually come across this before now. For a long time with my GP, I think because of my size basically, they put me down as a type two diabetic, which is wrong, I've always been type one, I was actually diagnosed when I was three. And it took a long, long time and in fact I'm not sure that they've ever actually corrected that mistake on my GP records. Now that's just one personal example but there must be lots out there of very similar examples where, you know, you actually trust health professionals to put the right data in, but mistakes are definitely made. So you'd have to be very careful about that and maybe give the patient a chance to review their data from whatever source it goes to, so they can look at consultants, they can look at GP entries, and so on and so forth, and actually correct that if it's incorrect.

GD: So maybe perhaps some kind of feature it sounds like people would like to be able to check on what data's held about them?

P29: Yes.

GD: Thank you.

P30: I'd like to add to that. I've had a problem in the past with updating the address of my current surgery. And because of that, all the consultant letters were being sent to the old surgery. And I've contacted so many people about that and I think it took me over a year. And I think that people

at the old surgery must have thought that I'm some kind of a jerk or worse for not taking care of that. But it was really annoying and it took a lot of effort, a number of letters and emails to have surgery address updated.

GD: Thanks for sharing that, P30.

P29: Another point that we haven't really touched as yet is things like the Ramora app, I mean, I agree with everyone else and it seems to be a great idea. However, it's very individual and I think I'm not sure you can actually take data from everyone that's using that and actually apply that to the disease as such. Because you're talking about individuals and how you're actually managing to get a good sample that is actually a sample of the whole population. It would be very easy to actually take the data that we get from those apps and then actually make an incorrect conclusion from it because you're not being that careful about who is using the app, whether they're actually representative of the whole patient population.

GD: Okay, I think that's an interesting point, P29. One of our other scenarios we were looking at a data donation platform. And something like this would enable much more data to be shared but the onus would definitely be on the research group, or whether it's a pharmaceutical company themselves, to ensure that they had a representative sample of people. But I guess what this particular platform would do would at least open that out and give people…for example, you could have something like this app, it doesn't have to be the Ramora app, it could be the FreeStyle Libre app where you had an option to consent to share your data with particular research institutions. How would you feel about that?

P28: My feeling is you're given the choice and I don't see that choice at the moment. My worry is, I think most people just simply don't ask where their data is going alone, that's both from a professional point of view. We collect masses of amount of data from patients and people just simply don't ask where it's going. Apps, I think it just brings it home to you and I think when you see just using social media how they infiltrate all these different platforms. I just think giving…you know, most people don't ask about benefits or drawbacks or where the data is going. So my feeling is that patients need to be asked. And if it was put into that app, certainly the FreeStyle Libre, you know, I think more people would probably agree to do it. I certainly would.

GD: Thanks, P28. Anybody else?

P29: I think that raises…that does raise an interesting question I think about, you know, where the data goes, because the NHS uses more and more outsourced [voices overlap 0:15:12].

P28: Absolutely.

P29: What are the outsourced companies actually going to do with that data. Because we've seen the latest information about, you know, the Excel

spreadsheet that we've used for the COVID apps, I mean, it's just…that was just really, really bad software design. I mean, no one would ever use an Excel app to…an Excel spreadsheet to do that sort of thing in the first place. So clearly, someone ought to have been looking at that far more, and that clearly wasn't happening there. There have been other examples I think where data has been lost in the NHS. So, you know, it does open up all sorts of questions about the data behind it, how it's being protected. And that really needs to be tightened up immensely I think from where we are at the moments.

VN: I think a few interesting points have been mentioned and I just want to open some more questions up. So we've been talking about a lot of the individual patient and then the question what happens if patients are not in a state of consent or they can't…they might not want to be burdened with the management of it. What do you think about that this kind of transparency might not only enable patients but maybe regulatory bodies to oversee and check, like, how data has been handled? So for example, if they are holding certain amounts of data and they're giving it out to research projects or to companies so that they can re-check what happened to that.

P29: That's an interesting question. As long as you are prepared to give…you know, that you can actually give them access to it and you can consent to do that, then that's fine, I think.

VN: I think P28 started also.

P28: Yeah, I think I was about to say the same as P29 really, I think the issue is consent to do that. I mean, I did read something, it was a while ago, I'm sure it was in…a very small amount of these companies actually follow…you know, they follow the standards…well, I don't know, are there any regulatory standards for these apps, I don't know, data sharing medical apps, I don't know if they're there or not. But I did read something that there's a lot of…well, there's a need for it anyway. And I think obviously medical [inaudible 0:17:57] sensitivity of data has huge…in my opinion, it has huge implications, particularly for young people who, you know, they may be putting stuff out there that can affect their life insurance, all kinds of implications.

Just to go back to the FreeStyle Libre, you know, it gives you an indication of what your HbA1c is, which gives you some prediction as to what…are you going to die early, you know. So there's huge implications about the data that we're sharing out there. So I think there needs to be some sort of regulation, I don't know whether there is or not.

P30: I think it's a good idea to have some regular independent audits conducted by a regulatory body, on top of regulations and legislation.

GD: Does anyone have on any views on who they would like to see there? So one of our questions is, what kind of information is it important for you to

know if you were to use something like this. Does anyone have any views around a body that would give them that kind of reassurance?

P29: I'm not sure there is one. Part of the problem is that when you look at things like Google and Facebook, they are outside of the medical profession as such, but they're still gathering vast amounts of data. And they will gather data wherever they can get hold of it, they don't really care about what it is, and if that happens to be medical data and they can make money out of it, they will do so. And I'm not sure how you can actually stop them from doing that. It has to be done at a government level I think, but I'm not sure how governments can actually…I mean, you know, the amount of money they'd have to spend and effort they'd have to spend to do that would be enormous.

GD: What about in terms of just this particular platform itself. So if there was the capacity for an overarching regulatory body to perhaps have a say over who was allowed to join and who wasn't, how would that sit with people?

P28: I think it's a start, it's something, yeah, I would welcome.

P30: Yeah, I agree, a new regulatory body could be introduced. And so I think eventually, all the small details could be worked out. But yeah, I agree that it would have to be on a governmental level.

GD: Okay. We're going to move on shortly just to have a look at some of the stakeholder perspectives. But I just wanted to quickly answer one of P26's questions. We might have to go back over these at the end. But you were asking if it could include social care as well. So there's no reason why not. This is a very exploratory piece of research and we're looking into, you know, what kinds of access people would need or want. So technically, there's no reason why not. Does anyone have any thoughts around expanding the platform so that it would include things like social care data? No?

P26: What have you thought about medication, Gail, you know? Because I'm thinking of scenarios such as when a patient's discharged from hospital and the medical regime can change, that can be particularly challenging.

GD: Yeah, I'd like to go back to that actually, P26, because that's included in our next scenario. So would it be alright if we…remind me if I forget, but that is in the next scenario, so that's something we're going to discuss then if that's okay.

P26: And I do think there are some no-no's with this, you know. I've put in the chat about sensitive issues, sexually transmitted diseases, I perhaps wouldn't have thought of that but I'm going to do a project on that shortly, so it's just flagged in my mind. You know, dealing…it's not going to be for everything, is it? There's going to be certain things that I don't think would be covered by it.

GD: Yeah, I mean, it actually raises a really important point that we need to underline to people is that any of the data that you share with somebody in the course of using this platform is done off the platform. So there is no physical data actually stored on the particular platform itself, it would be the platform gives you the capacity to enable someone to access your data in a different place, with your consent, but the actual data itself would not be sort of sat physically within the platform.

P26: Yeah, I still think if I was a 17-year-old girl that, you know, something like that [I would be backing off 0:23:22].

GD: Yeah, and that's a really important point, thanks, P26. It's something that we do need to consider, even when we're thinking about what information it's important for people to know, even when you give people that information it's still something as sensitive as, you know, a sexually transmitted disease, it might still put people off.

P26: Yeah.

GD: Okay. We did send you some perspectives, just to highlight really that people might have very different needs or concerns around using something like this. P28's mentioned that you wear two hats, a professional one and one as a patient. We're all patients and often people have other roles in life. So we just wanted to highlight a couple of comments that other people might make. If you don't mind, we'd just like you to choose one perspective that really interests you the most and just tell us what it made you think about really.

So we've got Ivor who's a GP. And this touches on a little bit around the integrity of the data that we discussed earlier. He's saying, you know, you can have two different kinds of activity trackers all saying that they're going to do the same thing. But one might say you've run for five kilometres and the other one might say 10. So he would like some kind of reassurance about how the data can be trusted, whether there might be some sort of accreditation process, for example.

And then Jade is mentioning, you know, if patients have complete control over what health data they share, that worries her a bit because if a patient decided not to share their BMI, for example, that might hamper her clinical decision-making process.

And then we've got Rose. She's worrying about if an app developer then goes on to sell her data to other companies without her permission.

And May is wondering if a company could actually say they're going to anonymise the data and then reveal her identity perhaps to the government, without asking her.

Finally, we've got Thomas who doesn't mind sharing his health data with his GP but he has concerns about the receptionist perhaps still being able to see his Fitbit data.

And then finally, Chang, who's a health researcher who thinks it would be really good if there was a lot more transparency around what happens to systems health data. Because at the moment, he thinks that people just, you know, don't realise, which I know is a comment that P28 brought up earlier.

So do any of those strike a chord with anybody?

P28: Yeah, just about, you know, the integrity of the data. Again, I found that quite interesting, particularly with my professional head on now. I'm an epilepsy specialist nurse, so there are lots of app sort of flying around at the moment and it's a huge problem, people come to clinic and they can't remember when their last seizure was, you know, and you're looking at potential drug changes based on that information. So a lot of patients do use some of these apps out there.

But again, one of the issues is that, you know, we only know what is shared. So you might have somebody, and it does happen occasionally where they won't enter their seizures onto an app because they want to drive. You know, and clinical decisions are made on that. Whereas maybe if you had that conversation face-to-face with a patient, you could tease out the information that you need that is not purely just on an app, nothing, you know. But you have to be…it is difficult.

GD: Yeah, that raises a really important both practical and ethical issue, it's quite a difficult one to navigate.

P28: Yeah, and I think probably a combination, I don't think it's something that will ever totally replace that face-to-face consultation, you know, where you're picking up non-verbal stuff that you can't get from an app.

GD: What about the element of the platform that gives people that control and choice over whether to share that data or not with a healthcare professional, does anyone have any views around that? So for example, we had the lady saying there if someone chose not to share their BMI, you know, that would cause some kind of difficulties. I've done an interview with a general practitioner who really likes the idea of this but has similar concerns in that you can only make decisions based on what a patient chooses to tell him, and if people have more control over how they share their health data, would that impact on their relationship then with their GP perhaps?

P29: I wouldn't have thought it would make much difference, to be honest, because, you know, if people decide not to share the data on the app, then actually it's no different to the situation they're in now. So what you're getting is, the GP would get the same data as they have now and they would continue to carry on that way. What this is doing is it's actually giving

them more data which will undoubtedly be able to give them a better clinical diagnosis perhaps, or certainly be able to give them more information to help the patient to manage their condition better. And I think that would be a conversation that you had to have…the patient had to have with the consultant or the GP. And it would be up to the GP and the consultant to actually say, well, you know, I could give you much better information if you do share this data.

GD: Okay. Thanks P29.

P30: I think I would do something like put a reminder of the website, you know, that the quality of data and the truthfulness of the patient will affect the treatment they get. And if someone doesn't want to reveal their BMI, perhaps put a warning that this is a crucial piece of information that your doctor really needs.

GD: I think that's a good idea.

P28: Yeah, I agree, that has to be built into it, absolutely.

GD: Excellent. We're going to take a break in a minute. Are there any other of the perspectives, I don't know if you want to just split them up, Victoria, that gave anyone cause for concern or they would like to comment on further?

P30: I am really worried about the safety of the data, that it may be breached or leaked and it may lead to a situation that, given my numerous diseases, I may be denied car insurance or travel insurance, or whatever. So I think the safety of data is crucial. And again, I wouldn't trust big tech companies with data because as we can see, for instance, in the States these days that companies like Twitter and Google are actively interfering in the presidential election, they are censoring people, [shadow burning 0:31:30] them, et cetera. So definitely wouldn't trust them.

GD: So if you had opportunities to make really sort of like specific decisions around what kind of data you want to share and with whom, would that help align some of those [voices overlap 0:31:51]?

P30: Yes, I think so.

P29: Yeah, I think so. Because the problem is not the data that's on the app or the data that gets to the GP, it's what happens to that data once it's passed on. And, you know, effectively, that's no different to the way that it is now. So what would be happening is they would actually be getting even more data than they are now. That's the concern, I think. Is that the sort of thing you were talking about, Gail?

GD: Yes. Sorry. Did you want to respond to that, P30?

P30: Yeah, so I guess that security is a priority and making sure that the data isn't used to any dishonourable ends. And a good feature that was

mentioned before, is to see a history of how the data was handled, [with whom 0:33:09], et cetera.

GD: Okay.

[56782]_CDIP_FG_27-10-2020_Part2]

GD: Does anyone have any strong views around this, what they think are some of the benefits maybe first of all?

P29: Oh, where to start. I have a lot of concerns around this.

GD: Okay.

P29: First of all, in terms of immunity. Looking at the way that COVID is actually being looked at, at the moment, you lose immunity fairly quickly afterwards. So if you're talking about an immunoglobulin test, immunity seems to disappear after about two to three months. So just because you have a test which shows that you've got antibodies at one time, it doesn't necessarily last very long.

Now that's obviously specific for COVID but it's probably also true of a lot of other things that we get. So anything to do with a vaccine, for example, you can go to the doctors, you can get a vaccine but it doesn't necessarily work, well, it doesn't work for 100 per cent of people. So in terms of actually having an app that does it, it tells you you've had an injection say, but it doesn't actually give you any real data, it doesn't give you any real protection against that.

The second aspect is that for these things to work on a population level, you have to have a lot of people actually using them. And as we've seen with test and trace, I mean, test and trace is a complete abject failure, a lot of the reason being that people just aren't using it. So those are my concerns.

GD: Thank you, P29. That's a good way of starting off. I just wanted to quickly sort of reiterate. In terms of the actual platform itself, if we think more sort of hypothetically around the fact that the platform's enabling you to prove that you've had something, so it proves that you've had…for example, if we move away from COVID just for a moment, that you've had an MMR vaccine, for example, sometimes when you go to other countries you have to have particular vaccines before they'll let you into the country.

And we're thinking around this as, because it's so difficult to change information on there and because it's stored permanently and it's immutable, that this would enable people to have some kind of digital-proof that's a bit more difficult to fraudulently create, so that you could prove in different circumstances your health status. And we've used COVID as, you know, an example because that's something that's very fresh in people's minds at the moment. And I completely accept the feasibility of the whole

kind of like immunity idea is a little bit…well, it is contentious at the moment and we have no proof. But if you can, I would like to just try and think around if that was possible, what would this app enable you to then do in your daily life?

P27: P27 here. I suppose [inaudible 0:03:28] for me, if it's a passport, if I'm getting it right, like a certificate, there'll be lots of countries in the future that if people want to travel to, they'll need to be showing or giving confirmation that they have x, y, z immunisations or treatment or support, because of issues that might stem from our current situation where we are with the pandemic. Implications about certain types of long term conditions or pre-existing conditions that might mean people are more or less likely to be susceptible in relation to COVID. So I think it'll be good for the travel industry, I can see that being good for travel and movement of people in different countries.

GD: Yeah, that's great. Thank you, P27. Earlier, P30, you were talking about wanting to share some parts of your health data but not other parts to other people. And one of the themes around this would be that in a social situation, sometimes we might want to share some of our health data but we're not sharing with a healthcare professional. I mean, we wouldn't want to share, or people might not want to share their entire medical history, for example, but there may be occasions in the future, you know, in the COVID situation or an occasion where you would want to prove to somebody else that you have either had a vaccination or that you are…for example, your blood type, or you are receiving a particular kind of medication. So this is, like you say, P27, more like a certificate, a passport, so you can prove to other people certain aspects of your health, without having to share absolutely everything.

P30: I think it's a question of how reliable that health certificate and how widely adopted it would be. It could certainly be used for travel purposes but it would have to be adopted and recognised by a host of countries. The other use I could see for it is occupational health, if you are applying for a certain job that requires some vaccine from you, that could be a proof of that.

GD: Any other drawbacks?

P29: The problem with vaccines though is that vaccines themselves are not always 100 per cent. So just because you have a vaccine doesn't mean that you're actually immune to whatever you've been vaccinated against. I think the best vaccines only takes about 75 per cent of the population, that's a really high protection rate, a lot of them are only between about 50 to 60 per cent. So what it's doing, it's saying that you've had something, you know, you have had this procedure that is acceptable in lots of countries but it doesn't necessarily mean you're actually immune to it, in terms of vaccines.

VN: I think that's a good point that P29 makes but that's not something that we can consult with any kind of technology if we want to show at least the

likelihood of immunity. But I have another follow-up question around travel and mobility. Because in previous focus groups when we had this scenario, some people voiced concerns if this would be widely implemented, if we think about that as a possibility, that it might [inaudible 0:07:48] people from accessing certain public spaces that are now open to all. So I wanted to open this up to you what do you think of that?

GD: Are you thinking here, Victoria, around accessibility, so if some people don't have access to this kind of technology, would they not be able to enter a country? Or are you thinking more in terms of if they hadn't had the vaccine or what have you, that people actually could be prevented from travelling?

P29: I think it's a concern. I mean, I do happen to know someone, a friend of mine that actually eventually relocated to New Zealand. And one of the problems they had there was that he actually had a cancer and they would not allow him in because he had cancer and he would need some sort of…a lot more health care from the country. And they actually said, no, because you've got that, then we're not going to allow you to settle. Now obviously, if you were doing something like that with this, you know, are you…have you had a particular disease or whatever, that would be a real concern to people if, you know, years down the line they decided to move to another country.

GD: Yeah, that's a really important point, P29, and, you know, huge life-changing thing for somebody. I wonder if something like this could be set up though to just prove what countries need to know. So that kind of…again, we're getting back to this controlling what you share and what you don't share. So rather than handing over your entire medical history to a potential host country, if they ask have you had x, y, z and you could prove beyond reasonable doubt…you could answer all of their questions without sharing all of your health data, would that help?

P29: I'm not sure it would. To be honest, if I was being told, right, well, any country that you go into insists that you have this in the first place that, you know, you have that data, I think a lot of people would be very unwilling to share that data anyway. And I think there's an awful lot of people who would just say, no, I'm not going to use that. And I suspect…I mean, actually we're seeing this I think with the COVID situation, there's a lot of people not bothering to get a test because they're going to be told you must self-isolate for 14 days. So they think, well, I can't do that so I'm just not going to get a test. I think it's the same scenario, I suspect.

P28: I suspect…sorry, I missed quite a bit of the earlier part of the discussion because I kept losing connection, but I suspect this information, P29, has always been required, or a lot of it has. But I guess on a positive with an app, it would speed things up, you know, with ease of getting that information to whoever needs it, that is one of the benefits I see. I know that, you know, in my professional head again, we get all kinds of letters to go for people's travels and it takes weeks and weeks and weeks to get this

sort of information out to where it's needed. So if there was an ease of getting that information for people, that's a good thing.

GD: Thank you, P28. I'm just trying to keep track of what P26's been saying, sorry, and listening to you all at the same time. So P26's wondering whether we should perhaps incorporate whether a patient's had a bad reaction to a vaccine. And some views around in the sort of COVID situation that national population rights might need to override a person's basic rights. And also, P27, yes, thank you, people who don't have access to phones with apps, they'll not be able to benefit, might be excluded from using things like this. That's a really important point, thank you.

P28: That is, yeah. And just to add to that, you know, people with learning disability, hugely under-representative group of people who miss out on all of this sort of technology, you know, needs to be sort of taken into account.

GD: Yeah. In fact, actually you've just reminded me, P28, a really important point that's been brought up in quite a few focus group discussions so far around consent, about capacity to consent, that if someone has an app and they can give their consent over the app. How do people feel about that?

P28: I think a lot of people…there's assumed capacity, isn't there, with lots of this and you don't really know how much of that that the person who's using the app has understood and what the implications are. And it's very difficult to know how to solve that, I think. You know, I mean, just to think of a scenario of Facebook users who put their false age in, it is a minefield really, capacity over social media [and apps 0:13:58].

GD: Are there any safeguards that people would like to see?

P30: I just want to raise another point about a backup system. Because as we've discussed, there are a lot of people who are excluded from these digital innovations and so there has to be some kind of a backup system. And it sometimes annoys me because there is, like, too much reliance on online solutions. And I know that's the future for the NHS, et cetera, that's the trend I see that now go online, diagnose yourself. Oh, you can't sleep, well, do a CBT course, that will help you. So although I'm very optimistic about some innovations, I still think that there has to be some balance and other ways of doing things.

GD: Absolutely, that's a really important point. Thank you, P30. One of the things about this is, without digital innovation, I suppose people in the past will have had paper records of their immunisations and paper medical records, which are much easier to fraudulently create. and with a system like this, it would be very difficult for an unauthorised party to get that information on there.

I just wanted to show you a couple of perspectives that we've devised relating to this particular scenario. And I'm just wondering, like last time, if

any of them really sort of shout out at you, if there's something that you want to comment on. If we just run through these.

So we have Clare. This is what I was thinking about actually when I mentioned that. So as an employer, this particular kind of app would help her take safety measures without running into problems with data protection. Because as a boss, she will be able to access the information she needs but she wouldn't…and check that her staff are immune, but she wouldn't see the rest of their medical record. But she's also thinking about obviously a future sort of scenario. If unemployment's really high, people might be trying to get fake immunity certificates just to get a job. So as an employer, for somebody like her, this kind of app might be quite useful.

And then we have Robin. So he's got some concerns about who's going to check and verify this. For example, who can the public speak to if something goes wrong. What happens if he forgets his password and there's no central authority there managing the situation.

And then we have Antonio who thinks it sounds a bit too good to be true to have all that privacy and transparency at the same time. And he's thinking about being a recent victim of a bank account scam and actually he could get his money back but what would happen if someone got hold of his health record in the same way. So that's a concern of Antonio's.

And then we have Emma who uses wearables quite a lot to manage her conditions. And sometimes she finds it embarrassing to share all of that with different people, especially…sorry, even with healthcare professionals. And what she likes about the app is that she could perhaps share some of the information that she wants to share, so that she can go to places and prove her immunity status but she doesn't have to share her entire record.

I just wonder, does anyone have anything that they'd like to say about any of those perspectives?

P27: I'm interested in Antonio's comments about…so who's responsible, who's the producer…or who's responsible, so if something gets lost, who's responsible for that? Who's the backup, you know? So like you say, who's the bank in this situation? I'd be quite interested in that particular aspect, yeah.

GD: Brilliant. Thank you, P27. Yeah, that's a really important point. Does anyone else have any concerns around that?

P28: I'd just like to say again about the…you know, apps are fantastic and I think it is the way forward but you have to have that people…there has to be a person or somebody who you can speak to when things go wrong. And as you say, if data doesn't look right, if something's gone wrong, you know. And I think we all have been in situations whereby you can't find anywhere, there's no contact details, there's no telephone number on these things. And it's almost like they don't want to be contacted, you know, it's really

difficult. And I think if anything, it has to have that person bit built into it, I think.

GD: Does anyone have any views around who would be a good person, who would you have faith in to kind of oversee something like this, or organisation [obviously 0:19:45]?

P28: Well, just to go back to the NHS track and trace. It has the NHS logo on it but I don't know if people actually trust that. I think we all trust the NHS bit of it but, you know, truth be known, it isn't the NHS, is it. So obviously, my…I would prefer it to be an NHS focus, platform or whatever you want to call it. My feeling is people have less trust when things are sort of, you know, contracted out to companies. But that's just my opinion.

GD: Thank you, P28.

P29: I think it's interesting, it was mentioned I think right at the beginning of it that it was very much local data, it wasn't actually centralised at all. That does beg the question who actually is responsible for the data in terms of I suspect it's probably just your GP. So I guess the protection would have to be put in at GP and user level, so it's actually up to the GP and the user to make sure that that can't actually be shared with anyone else.

I don't know, it's an interesting question, I mean, you'd have to go and get your…it has to be verified somewhere so you have to trust the people who are doing the verification. You know, have they been approved by the government, presumably so, or who do they actually get approved by. So you've got at least three sort of levels there that are obvious potential data leaks that therefore have to be trusted, which makes it easier than letting the central people have it because once it goes out of local aspects then, you know, it's open to anyone to get hold of the data potentially.

GD: Yeah. You've raised some really important points there, P29, that highlight some of the different features actually or possibilities around this particular platform. So potentially, you could have a situation where you are just in a direct kind of relationship, you're having a transaction with another party. So as we said before, none of the data would be on the platform.

So if we go back to our earlier example with Ramora and you have [Malika 0:22:22] and she's choosing to share that data with her GP, via the platform. But like you say, P29, so there's maybe two particular scenarios around that. It could be verified by some kind of outside organisation or it could be your trust would be in the person that you're transacting with to look after that data. And I'm just wondering what…how people would make those kinds of decisions, what would they think about.

P27: I think you have to think about the law and how it stands around data protection and data sharing. I mean, there's national guidance around what can be shared and who with and when are we able to…when is our data able to be shared more widely without our so-called consent. And that's

usually if it's about our own care, the GP can use our data to pass on to secondary care to improve our own care. But all those things collapse when we have a situation like this. If there's a national public health disaster or concern, then permission, that goes out of the window.

So I think it would be a bit retrospective to go back to the transactional relationship between myself and my GP about managing my care and sharing my data because there's a whole raft of information governance around how data is to be managed, shared and stored. I don't think you can push it back to the individual. It sits within the law about how it's shared and who manages and who's responsible. For me, it's about if the data gets lost and there's any leaks and breaches, who's responsible for…who do you turn to about that. And it won't be the app company, it has to be the person that commissioned it. And as P28 said, it should be something that we have interest in, we're stakeholders in, which is the NHS, we all pay our taxes into that, we pay for that service so it needs to be doing things that protect and look after us, that's what we pay for. It's what we pay for.

GD: That's all really valid, P27, and that is the current situation. And we will always be falling back on the more in circumstances where things don't quite go to plan. I guess with this kind of platform, what you would have, however, is a permanent record of you're either giving consent for specific kinds of data to be shared and then if that person does not do what you consented to do, you have a permanent record there that proves that.

P27: Thank you.

P29: I think that's one of the questions. I think the other question there is how do you actually get the genie back in the bottle. So once your data's out there, is there any way you can actually get that data back once…should there be a data breach. I think that's rather more…I mean, that's effectively what we were talking about with the bank scenario. You know, the bank scenario was that you actually lose the money and the bank actually takes that loss and puts the genie back in the bottle. With health data, I don't think you can do that. You can't actually…once it's been let out there, you can't get it back again. And that's a real cause for concern, I think.

VN: Can I just ask, because I think P27 and P29 both make very valid points on the question of responsibility and accountability as well. I was wondering in this particular scenario, if somebody, for example, would take a phone with an immunity pass on because no identifiable data will be shared. And then I take my roommate's phone because she's immune and I don't want to do that, and then I go to a hairdresser. And it turns out that one has COVID or something like that and it's like, but then it wasn't my roommate, it was me who went there.

So this kind of idea that people might try to cheat the system, not only like big breaches and security leaks might be happening, but also that people might try to cheat. And then who would be accountable to kind of keep a track on that and who's been put in the responsibility position and who's

burdened by it as well, because we don't want everybody to become little police trying to check each other's information all the time. I was wondering what other people think of that as well.

P30: Well, wouldn't that then fall within the realm of identity theft and computer crime?

VN: Yeah, definitely.

GD: A couple of people have mentioned gatekeepers. I'm just wondering maybe if everyone could comment on this. The idea of this platform could run with everybody being gatekeepers of each other and no outside authority being involved. Or there's a potential to make it a bit more private and have a gatekeeper. I wonder what people's feelings are around that.

P30: I would very much be in favour of having an official gatekeeper, someone that could be held accountable for any leaks or breaches, and someone who could deal with any queries that people may have.

P28: I agree, I think there needs to be a contact point for anything like this. Because things will go wrong, you know, it's not infallible. So there has to be a point, somebody needs to be accountable and responsible for this, I think.

P30: I think this is too serious an issue to be left alone, it has to be monitored and regulated.

GD: What about you, P26, do you have any feelings around that?

P26: I've just put in the chat that personally, I feel, you know, uncomfortable about the whole telling tales during COVID, neighbours reporting each other if they do something wrong. And I know that's perhaps the way that a pandemic is controlled in society but it just makes me feel very uncomfortable. If I saw somebody using somebody else's app, would I report it, you know, I'm just not sure about these things at all. Ethical issues isn't it, there's no easy answers.

GD: No, not at all. What about if that responsibility was taken away from people and there was a central gatekeeper, would that help or hinder that?

P26: Yeah, it would. That would reassure me but I don't know who that gatekeeper would be. There doesn't seem an obvious candidate for that at the minute.

GD: Okay. Very diplomatic, P26. I've just noted your comment there, P29. So you're wondering if this could be solved by keeping the data elsewhere, i.e., nothing is saved on the phone or tablet?

P29: I mean, going back to what we were talking about, you know, you have a test from someone at the pharmacy or whatever and it's sent to the GP.

The obvious answer, I would have thought, is to actually keep the data somewhere in a very secure database that you, the GP, whoever needs access to it has got. And actually what your phone does, it just looks at the data. So you've actually already got security built into that, you know, you have a password, you have fingerprint technology or whatever to actually access that data. So even if someone stole the phone, they would still not be able to get that data unless they had your password or fingerprint.

GD: Yeah, that's an important point. I think perhaps because we're thinking around something new here, there are also a lot of existing either legal safeguards or technological safeguards that are built into some of these technologies that we already use. And I guess, I'm wondering what people's feelings are around, you know, whether they should stay in place as they are, and all this particular platform does is open up more opportunities to kind of have communication or to share data or to choose what's happening to their data, without any extra sort of reassurance, so we rely on what's out there currently, as in, you know, the General Data Protection Act or it's the security that's inbuilt into your phone. Would people want this platform to offer something extra?

P27: Well, I think I'm sure there are…I mean, if you were to take the hairdresser scenario. I'm sure there's stuff that can be put on there that could identify you. I don't know whether it's even an address, I don't know, something that's not highly…hugely sensitive data that local businesses could use. I don't know, I don't think it needs to be that complicated. I think, I suppose, I hope most people wouldn't abuse it, I don't know. But I'm sure there are things that could be built in, an NHS number, I don't know, that you could link to some of the paperwork that you've got, if you had to do that, you know. I think there are probably ways round it.

P29: I think generally, the NHS data that's there in the first place is actually pretty well protected, certainly at a local level. Once it gets outside into other companies, that's a different matter. But generally, I mean, you know, the stuff that the GP has is reasonably well protected, I think. I've got a couple of apps that I use from the NHS for things like prescriptions and so on and so forth, and I'm comfortable that even if someone stole my phone, they wouldn't be able to get on to that sort of stuff because it's password protected. Now, you know, if you choose to use a very easily guessable password, you have to take responsibility for some of your own actions, I think. You know, you can't say, well, okay, someone's stolen my data, I had a password that was password, you have to take responsibility for that yourself, the individual does. So it's actually up to the individual to protect their own information and to take relevant steps to protect their own information.

GD: Is there any kind of feature that would be helpful to support people with that, do you think, that could be incorporated into the platform itself?

P29: I think modern phones are actually very good at doing things like, you know…well, a lot of smartphones these days allow you to use fingerprint verification.

P27: Face verification now as well, isn't there?

P29: Yeah. I mean, that seems to be the easiest way to do that.

GD: So rely on sort of the technology rather than the actual platform itself?

P29: You've got to rely on people to actually get a decent password, you know, a good, strong password. That's ultimately what you have to do. Now whether that password's your fingerprint or whether it's a collection of characters, it doesn't really matter, it's got to be a strong password. And that's up to the individual to take that decision.

P30: Yes, I agree and perhaps implementing a two-step verification process on top of that on top of a strong password.

P29: Yes, agreed.

VN: So…

GD: Go on.

VN: Sorry. Just so that I understand it correctly, are people kind of like in favour of more identity checking and using biometrics like fingerprints or facial recognition to do that? Is that…because that's kind of like the opposite of the scenario where it's like boiling down to not sharing a lot of data and especially not personally identifiable data.

P29: I think it's more of a question of, you know, well, these days on your phone, you have an awful lot of data that's nothing to do with health and you need to protect that data, that's what it's all about. And this data should be protected in exactly the same way as things like bank data and so on is protected. Having said that, banks are particularly bad at protecting data, you know, you've got a cash card with a four-digit pin on it, it's absolutely useless, there should be much more protection around that. As P30 said, you know, using the two-factor verification is a much more safe way of doing things.

P30: I think fingerprint and face recognition would be just used to unlock your phone if it's supported. And for the app, for the platform you would still use password or a code from a text message or email.

GD: There was a couple of points in the chat as well I just wanted to highlight before we wrap things up. So P27 is wondering what the benefit is perhaps to the company producing the immunity pass, as they would have all of this data. How do people feel about a company having all that data through the platform?

P29: Are we talking about the company writing the app or a company checking whether potential employees have immunity?

GD: Do you want to just clarify that, P27? I'm guessing the company producing the app.

P27: Yes, that's correct.

P29: I don't know, I don't know what their benefit is. I suppose doing good for society. I mean, I can't see they're going to make any money out of it.

GD: I guess the same goes for all of these companies, app developers, because these are professionals that we're also interested in talking to, in that if somebody produces an app that helps society in some way, whether it's at an individual level like the Ramora app that we looked at initially, or it's a unity certification app that patients…general systems will be in a relationship then with that app developer and sharing health data with them. Essentially, we do that already with Fitbit and things like that.

VN: Yeah, I think that we have to kind of clarify that we don't have a specific business model or something in mind with that app. So this could be something that is a public service that could be paid by a similar…or if it would be integrated in the NHS. So this is just a scenario which we kind of made up and we don't have a business model in mind for that, so we don't want any kind of ulterior motive just to make money with that kind of app or scenario. So it could be something that is being paid by the public and then it gets integrated into already existing services such as the NHS. If that clarifies it a bit.

P27: Yeah, clearly, you wouldn't want an app, a chargeable app for something like this, you know, because it defeats the object, doesn't it?

P29: Well, I guess that depends, I mean, that would have to be looked for in the model. Actually, if you could say, you know, right, you can download this app, it's going to make your life a lot easier, rather than having to go to the GP to get letters and so on and so forth, it costs you 50p to actually download an app, I think a lot of people would be prepared to pay that.

P28: And it's the equity I think, you know, people…without smartphones, it's not equitable. People with learning disability, you know. I have issues with chargeable apps for health-related issues. Again, my opinion.

P29: Sure, yeah. I mean, I think as we said before, there has to be some sort of backup, so you couldn't just use this app, but it might be in addition to paper-based…you know, presumably paper-based stuff. But yes, you'd definitely have to have a paper-based backup.

GD: Thanks, P26. She just mentioned she would prefer it not to be chargeable. It's a really important and interesting digression actually, we've not even

thought about that, so that might be something that we mention in the next focus group. Because as we're going along with these different discussions, people are raising really important and interesting concerns or views around things that we haven't thought of, which is exactly why we're doing this today. So that makes it really, really useful to us, thank you.

P26: Can I just say about the exploitation aspect of it because, you know, there may be a company who are very altruistic and that's the way it's developed initially and, you know, that might happen for several years. But if there was a change of company and they were perhaps not so altruistic, is there a potential…I mean, I don't know because I don't know how these things work, but could there be a potential for exploitation?

P29: Definitely. That's exactly what's happened with Facebook. Facebook initially was privately owned and they didn't have that many adverts. Now it's being publicly funded, now it's on the stock market you can actually see that they're actually out to make more money by advertising [inaudible 0:43:28] and the platform's changed completely.

GD: Yeah, that's a really important point. Thank you, P26 and P29 for raising that. I wonder, would you feel reassured if you had some kind of regular notification to remind you about your consent, would you want to monitor it on an annual basis, so that you could perhaps change your mind if circumstances changed?

P29: Yes, I would.

GD: Do you have any ideas around how often, so not to burden the patient?

P28: I think every time you log onto it, you should be asked about your consent. I don't see it as a huge issue, just a tick box, you know. That's my opinion.

GD: Great. Thanks, P28.

P29: Yeah, I think it would have to be slightly different to that, I think. Because looking at my own particular scenario, we don't go abroad every year, so actually I might use it once if we went to a country that needed it. And then actually I would…normally speaking, for things like that I would actually just remove the app from my phone, once we got back, I'd just delete it.

GD: What about you, P26, would you want any kind of like reminder to sort of check your consent preferences, or do you think that should just be left up to people's own [voices overlap 0:45:07]?

P26: I think I liked what the lady said before, that it's there every time you log on. But I've got to raise the question of data up to that point because that crops up a lot in research whether the retrospective data, you know, whether it's still kind of accessible and then it stops at that point that you stop consenting. And, you know, I'm just a little bit concerned about the past data.

GD: Okay. Yeah, that's a really important point for us to think about. What would be your preference?

P26: Well, in research, we sometimes do ask patients to consent for that data to still be used. But again, in research, it's for altruistic purposes, so I'm not really sure. P27's a lot more ahead of this sort of thing than I am, I'm just not sure about that.

P27: No, I'd agree, I think that whole consent, at what point. I think P28's suggestion is a really good way forward that every time you log on, you agree that the information you're using or putting in, you're happy for that to be shared or to be seen or whatever, you're giving consent at that point and at each point going forward.

GD: Thanks.

P26: And what about the past stuff, P27?

P27: I think it's at that point isn't it, so it's got to be contemporaneous hasn't it, so it's at that point you're giving permission. So it has to be there from the time you started from go live, anything you put in at this point going forward, I'm happy with. So I think from go live, from the minute you start, you're building your profile going forward.

GD: Okay.

End of transcript

**File Name(s)**	57062_CDIP-FG-06-11-2020-part1<br>57062_CDIP-FG-06-11-2020-part2
**Total Recording Length**	83 minutes
**Number of Participants**	8 (P31-P36)
**Final Word Count**	13,390
**Total Number of Pages**	28
**Date Completed**	19 November 2020

[57062_CDIP-FG-06-11-2020-part1]

GD: First of all, maybe if we can consider what you think might be the major benefits or drawbacks of using this? I don't know if anyone has any strong views. Is that you, P31, are you putting your thumbs up?

P31: Yes, I was just going to say a major benefit would be that you can send your data to your doctor before your appointment, it means it could be a quicker appointment because assuming they've had time to read the data before you go, you can get straight down to the nitty-gritty of the appointment rather than waffling through all the, oh, this happened on that day and that happened on that day, so that would be beneficial and also it would allow for more, as we're doing now, online appointments to share data for that.

However, drawbacks, you're saying that the GP could save it to your NHS record, my GP is really not computer efficient. What are the chances of them saving it to somebody else's NHS record? And my other query is Victoria's saying about it's an immutable record, what happens if your GP has added something in error? That's a big drawback.

GD: Thank you, P31, did you want to come in there, P35?

P35: Sorry, yes, I was just going to say what I was thinking, what happens if the patient recorded something in error and they thought, oh, I shouldn't have put that in the app, and then it's gone through onto this platform and you're saying it can't be removed. What happens then if the patient has made some errors?

GD: Yes, they're all really valid concerns. Does anyone else have anything to say about that?

P32: I like the idea of it. I think it would be so much better instead of having to sit there trying to remember everything all the time. It's that constant thing, somebody asks when did something happen, I mean, I've been in hospital recently, it was only days before something had happened and I couldn't remember exactly what day it was. Had I got that on my phone, I could have just brought it up and said there it is, this is exactly what happened on this date and I would have found it really useful because I actually use…I had a problem with my blood pressure at the time and I did use my data off…I've got a smartwatch and I could use the data off that and show them which, although it's not that accurate, it was actually really useful because I could just find the information there and then and it stops you from sitting there and wondering, I can't remember exactly when it was. So I do like the idea of it and I think it would be really useful.

GD: Great, thank you.

P34: Yes, I'd like to add to that, if I may, with regards to tracking past history, with my experience with my auntie, one of the things we were asked to track

is how many falls she'd had, and there were various types of falls, ones where she'd put herself on the floor or she'd literally fell but trying to remember that three weeks down the line when you meet with the falls team, was it day or night, how many times and typically we wouldn't know, we'd have to guess at three or four or whatever it may well be. So if you have an app where you could record that. I mean, I suppose you could write it down on a piece of paper but I just think that would be useful information to go back on.

GD: Great, thank you for sharing that, P34. Has anyone else got similar…?

P33: I agree. I think it's a really good idea. I think especially from the smartwatches and the Fitbits to be able to…I know that they're not 100 per cent accurate but to be able to share your BP and your activity or whatever it might be, whether it's prior to a meeting with the doctor or at the time, I do think, yes, it's very helpful, but I agree with the other comments as well in terms of if you can't change anything on there that would be…I mean, some people are a bit more technical than others and it's very easy to make a mistake, not only for the GP but for the user as well.

GD: P36, did you have your hand up?

P36: Sorry, I had an incident where I actually got a hospital letter from the doctors with my medical records and being the nosy person that I am, I read them, and I found on there a totally inaccurate statement which I could prove, and when I did challenge the doctors over this, they said, oh, we're very sorry, it must have been when they were typing it in upstairs, i.e. a clerk was typing this in, which is an appointment which is absolutely nothing to do with me which could be detrimental to my record. That bit I do worry about, by not being able to get anything retracted, it doesn't sit well with me.

P33: Yes, I think somewhere there's a note at least that would pop up where you can amend it to say that this is incorrect, almost like it flags it up, that might make a bit of a sense, but yes, it's that worry that something's in there and it's incorrect, and I think if as a user you could see it and you could read it yourself that would make it a bit easier because at least you could challenge it without worrying that all the data on there is incorrect. I suppose as it stands, the GP might at the moment have incorrect data on the system and we wouldn't know it because we wouldn't get to see that.

VN: Maybe I can come in for that. So while it is currently not about deleting, but what we can do is add of course a correction to the data, so instead of deleting incorrect data, you would have a correction that then pops up higher than the incorrect record, so that's a possible intervention for that.

P36: I think it will be essential.

GD: P31?

P31: Would it be better to have the pop-up next to the incorrect data? Because my GP just looks at the one bit in front of him, they don't scroll through to

find if there's any addenda further on, so it would have to be actually look at that bit of data, and it would immediately flash up that this is incorrect, otherwise it's not going to get noticed.

VN: Okay, yes, that's a good point.

GD: How do people feel around that? I mean, it's a benefit and a drawback, really, of the blockchain technology itself, so if you want to go back and check what somebody's done with your data, nobody could ever delete, so if maybe an unauthorised party was trying to access your data or something like that, you could actually go back and check because the blockchain actually records all transactions on the actual chain itself. However, as Victoria's explaining, you can never delete anything, really, without great difficulty, but what you can do is always have the correct data. I'm just wondering how that sits with people?

P35: May I, please? I was just thinking with my GP, they're very, very poor at following things up and it worries me that they wouldn't have the capacity to get on top of information adequately because they're not doing a very good job at the moment, and if there's more and more information stored, there needs to be some sort of…I don't know, maybe funding of GPs so that they can get on top of this technology, you know, that comes, and so that they're more able to, well, for one thing follow things up rather than just relying on the patient to ring up, let's say, for blood tests results or to follow something up, a condition, because my GP, they just don't follow anything up. They're very poor, really. So if they're not getting on top of things like that, they don't seem to have any alert in place at the moment on the computer system that says to them, oh, the patient hasn't rung up about this blood test, you know? They should be ringing you up, I think, and following it up.

So they haven't got on top of that so far so that worries me that the more and more information that would be on such a platform, you know, they're not geared up for it.

GD: Yes, so maybe to summarise and reflect back, P35, you're concerned because you don't have a lot of faith in them already, in…

P35: Yes.

GD: …following things up with regard to data, so you're perhaps looking for more assurances that they would look after this extra data properly.

P35: Yes.

GD: Did you want to come in there, P31?

P31: Yes, following on from P35's point, there are over 9,000 patients registered at my GP practice so that could represent a significant portion of data coming in each day. Which doctor's going to have time to go through that?

I would not be happy if there's some junior assistant going through and interpreting my data to highlight to the doctor, oh, look at this, because they're completely inefficient. Now you get a sample, I have to do regular sputum samples and I get some junior…oh, it's come back as positive, so what does that mean? I don't know. Well, what shall I do? I don't know. I'll contact the doctor. And then the doctor never gets back, by which time I've got pneumonia.

So to be deluging GP practices that are already incredibly overstretched with further data would be a massive concern because it's time-consuming, things could be missed and who would be looking at it? Would it be a GP looking at it? In which case that's taking away from patient care, or would it be some specially trained office junior who I wouldn't want looking at my personal data?

GD: How would you feel if you could have a lot more control around who can actually view this test result data? So as Victoria highlighted earlier, there's a possibility around smart contracts which is an automated contract which would only let specific parties access specific kinds of data in a contract that you would set up yourself. How would that sit with you?

P31: That would be absolutely essential, but then would my GP actually have time to look at the data? That's the problem.

GD: That's a fair comment. So that leads us into the choices and controls. Does anyone have any other ideas about are there any other controls that you would want to have over this kind of data?

P35: May I raise something?

GD: Yes.

P35: I've got an elderly aunt, there's only me and my aunt left in our family, and she's not tech-savvy whatsoever. I have tried to get her onto a smartphone but she just will not retain anything. So how would this sort of thing affect my auntie if she doesn't have any technology already and she doesn't want any technology? Would somebody have to write to her and say, you know, what information would you like us to share from the GP or…? That's something I was wondering.

GD: So in that particular instance, how would you feel about her being able to give that authority to somebody else perhaps to support her with that? What would be helpful in this instance?

P35: I'm not sure, really. I think that comes along with power of attorney issues as well when somebody's getting elderly as well, doesn't it? You know, authority to help them that way.

P34: Yes, if I could chime in on that, with regards to, I get what you're saying, my auntie, no chance of using a phone or anything along those lines, however

even if the data that was collected wasn't 24/7 100 per cent because my auntie's not logging it herself, from my perspective, even half the information would be better, so even if I logged it, my sister logged it, the health professional that was seeing her at the time logged whatever issue, that amount of information would still be more useful than what we've got at the moment which is nothing. And I personally wouldn't have an issue with social services, the healthcare professionals inputting that information, depending on whatever the information is, into an app to update and share as and when they saw her.

And interestingly, blood test results is a problem that we've got at the moment because she had a blood test done in [City 1] and [City 2] can't get the results, so we're having it done in [City2] again.

GD: So how would that affect you if perhaps on her behalf with your power of attorney you were able to authorise access for other authorities to access that data?

P34: Absolute, it would save so much time and effort, simply sharing between boroughs would save a lot of time and effort. And between social services, DWP, et cetera, and the healthcare professionals is a very useful tool.

GD: Thanks, P34. P36, were you trying to say something earlier, sorry?

P36: Sorry?

GD: Were you trying to say something earlier, sorry?

P36: I've lost the train of thought now.

GD: Don't worry. We're not putting you on the spot, it's fine. I just thought I might…

P36: Old age.

P33: One thing that I'd add to it is we're talking about obviously people who aren't tech-savvy, who can't do these things and maybe have power of attorney or have a member of a family who can help. What about those who don't have that support, who don't have anyone they can rely on? A lot of individuals who are vulnerable who their GP is the only person that they go to, so those types of people would potentially miss out on this because they wouldn't necessarily understand or want to have this information and there's nobody to explain it or do it for them. I think that group, you know, would miss out on technologies such as this.

GD: Absolutely, that's a really important point, and it's something that lots of other people have raised in other focus groups, and we've been thinking around how could we make this a bit more inclusive. Some people have talked about things like maybe making videos, YouTube videos on how to

access this. I don't know if anyone else has any other ideas or concerns around that.

P34: I just think that generation, the octogenarian generation, they wouldn't even know how to watch a YouTube video, so I think any form of…and to be honest, having tried it with my mum numerous times how to work a mobile phone or an iPad, hours and hours of training and it got nowhere, so I think there's just a… I think maybe moving forward we won't have a generation of people who aren't tech-savvy, so maybe it's a moot point because in 20 years' time or ten years' time, it's likely to be a completely different kettle of fish from a tech point of view.

GD: Yes, thanks for that. P31, did you have your hand up?

P31: Yes, so we've got the older generation who may not be tech-savvy but we also have a lot of people who actually can't afford mobile phones, haven't got the data on their cards because they choose food or data, and so that would actually be excluding a section of society which generally have greater health needs because of their poverty or whatever, their situation, and they would not be able to be included in it, where actually they're probably the more important people to be included, and that would be a massive issue of how could you get them…?

And then also people…my husband, when he was working, he was working 80, 90-hour weeks, he's done that all his working life, when he comes in he would not have time or the energy to put anything onto a phone, so again time issue is another thing that would have to be taken into account and it would have to be very speedy, very easy, but accessible to all is really important.

GD: Yes, navigating this digital divide is an ongoing problem with lots of different digital health applications, and I know the NHS are very keen to try and address this issue and there is no quick fix answer. But yes, it's a really important point and we are really conscious of that. I just wonder how would you feel for somebody that didn't have that kind of access, if they were able to do this through their GP, for example, in their local practice? When they were registering they could decide how they wanted their data shared, and they could access the platform via their general practitioner.

P31: But again if you've got a person suffering from mental health, they may not want to go near their GP, whereas it's really, really important to log that they're taking their medication, how they're feeling, especially if someone's paranoid, they're going to look at that phone or computer and go, oh, no, thank you.

GD: Good point. Okay. Just thinking around…sorry, go on.

P36: Even if you go to the GP when you initially register and say, yes, I want the data kept, it's only going to be the current data that everybody has nowadays. You as the person won't be adding to it.

GD: Yes, I mean, there's various different ways in which this particular platform could be used, so this is just one of our ideas and scenarios to be thinking around. So if somebody doesn't have either a digital device or some kind of app where they're monitoring their health, it's a bit of a nonstarter, really. When we come onto the next scenario, we're going to have a think about sharing health data more generally and more widely, and it might be more of an issue around that. But just before we do, I was just wondering if somebody was to ask you to use a platform like this, what sort of things would you take into account? What information would you want to have? What kind of reassurances would you want in order for you to even consider using it in the first place?

P32: I think you'd want to know who owns it, who it's run by and what the security controls are, so passwords, where it's going to be stores and who you're going to give permission to, you know? Is it a pharmaceutical company, the NHS, I think that's a big thing so you can see to trust that person and the app in the first place because I wouldn't just download an app without knowing exactly who it is that's behind the app, so if it was something like the NHS, I'd be more likely to trust it than a company that might want to make money out of using my data. So I think that's part of it, where it comes from.

P36: Yes, I think that was a problem when, a couple of years ago the government tried to force everybody to allow their data to be shared and it was just purely a commercial gain business which I reneged from, I wouldn't allow it and a lot of other people. This is my only fear is that…although I'm fairly free with my health records, anybody can have a look at them, I don't care, but it's who are they selling the information to, because it is a commercial proposition at the end of the day. Because unless the NHS is running it, it's got to be a private company who's going to make profits.

GD: So how would you feel about if you knew that this particular platform on it you could track who has accessed your data, so if it has been gone on further than that, you'd have some kind of mechanism and proof of who has accessed the data? How would that make you feel?

P32: I think it's a good thing, but it doesn't stop the fact that if that's happened, the damage potentially is already done, so I'd want that trust in the first place to know that that's not going to happen and then I can see that it's not happened. It would be more reassuring to know that I can see nothing's gone wrong rather than trying to fix it when it's happened.

P31: But also I'd want assurances in the future that it's, okay, it's all nice and safe and secure now but what happens if it gets sold to China or somewhere like that? I don't want my data going there. And as P32 said once your data's been accessed, it's accessed, there's no good pulling the plug after it's been accessed, I'd want to know before it was going to be accessed.

P32: I think there's been breaches as well in data in the last number of years. I mean, look at Facebook and Google and all the rest of them that have had their accounts hacked, I think people would really…you don't want to be able to have to look at it and see that something's happened, you want to make sure that the privacy is in place before you even step into it, because it would just be a minefield, wouldn't it?

P35: I just wanted to raise about with the pandemic situation and the COVID app, you know, the NHS COVID app, there's been some errors on that when they put it out and I even have them on mine until it was updated, and I don't think the NHS itself actually built the app, and that concerns me that there's some loss of trust there, certainly from my perspective, because I think, well, I used to be in computer programming many years ago, designing software programmes, so it does interest me, this discussion, and that worries me that there's been a loss of trust with something associated with the NHS. So I think that's a big priority that you would need trust as the number one thing, you know, for a platform.

GD: What kind of information would be important for you to know in order to make that decision about trusting something? And, P32, you were saying something around it's key that you are able to trust, if we're asking you to trust in the technology rather than a person, so if you think of this as almost like a cooperative where everybody involved in the platform can access or can speak to each other, can communicate via the platform but the data itself is not actually held on the platform, what you're doing when you're using the platform is you're enabling someone, you're giving them permission, so it's like a way of recording what permissions you have granted. How would you feel about trusting the technology rather than somebody like the NHS looking after your data? What would you want to know?

P35: I was just going to say you'd have to be trusting that they're competent. Another one that springs to mind, the Post Office scandal where people went to prison because of the Horizon system, and how a body such as the Royal Mail could get into…well, have such a…not the Royal Mail, the Post Office, have such a system that led to people going to jail, such a prominent public company, I'd want to know that whichever company was building this platform was top-notch. I think I'd want something…possibly some pop-up every time I used it to remind me who would be using this data, I think, not just a sign-up, you know, some way of just reminding me, the patient, of where the information is going perhaps.

It might make it too complicated.

GD: Has anyone else got anything they want to add?

P34: Yes, I'm just going to chime in in a completely different sort of way again. Personally I think an app would be a lot more secure, I completely agree, it needs to be done in the correct manner with the right company and so on and so forth, but I don't know whether anybody's experienced with regards

to the NHS that you have had your records mixed up, you have got somebody else's information, they have given you somebody else's discharge papers, or somebody else's DNR, which is what happened to me. So personally I've got more faith in an app than I have with the admin team in a lot of…but particularly [City 1 Hospital], not that I'm dissing [City 1 Hospital], it's a great hospital but their admin is poor.

So I've personally got more faith in an app regardless of the concerns around an app than I would have in somebody pressing a button and sending a letter out.

GD: Thanks, P34.

P34: Which I know can get mixed up on numerous occasions.

P33: Yes, I suppose you'd have more control over it, wouldn't you, if it was an app, because you'd actually be able to see it physically and if there was an issue with it, you'd be able to address it but you'd actually be able to see everything on there and show that to the people that you needed to show it to rather than paperwork.

P32: Yes, I think when I've used that patient access which I've only recently got with my new GP, that's actually quite nice because I can now look at test results and things that doctors have never flagged issues before that I can now see is a problem, so if I had an app that I could look at stuff I myself would be a bit more in control to be able to follow up and say what does this say and what do I need to worry about this for? Whereas in the past, the doctors haven't had chance to look at it, so it would be nice to be able to, yes, rely on the app or a person as such.

P31: Again that's assuming that the test results get… Our GP doesn't put test results up on patient apps, you're still none the wiser, so again that's a further onus on GPs or whoever to be putting the data on, and if it's…I'm not being derogatory to medical receptionists but if they're not medically trained, they can make errors in values and things like that, and units, and again that's an added concern.

P35: May I just add that as well, echo that point? My GP, they stopped using a lot of the patient access areas on our system, so for example, during the pandemic, you can't actually book your appointment now on there, which I find really odd when you would have thought there would be more reason to be able to do that during a pandemic, but they've taken that off and they don't put test results on my records either. They don't use it to the full capacity.

P31: All we can do is order a repeat prescription. That's all we do on patient access.

P35: That's right, yes, very, very limited what they use it for.

P36: Same, you can't even see my emergency drugs on the patient access. You can see the general prescription, but you can't see any emergency drugs that I have, and like you said, there's no other information other than the prescription on patient access. So this is my biggest fear is that you develop a good system, but it falls down at the GP being unable to input the information.

P32: I think that's where the app could be something that doesn't rely on a person to input it, if it was an IT thing which obviously can have its errors, but at least if it didn't rely on a person, that would be a better way of doing it, so it's automatically on the system.

GD: Just to clear up, so this type of platform wouldn't store that kind of information…excuse me… What it would enable you to do is to make decisions around who can access that. So for example, something like test results, if you wanted to let a consultant see them as opposed to your GP or you wanted them to show them…maybe with your example, your auntie who's accessing social care, you're wanting to share them with them, you could give permissions on this, and I just wanted to go back to something somebody said about trust. How would you feel if the people that were transacting or communicating on the platform itself, if there was some sort of rating mechanism or some way of establishing how trustworthy a particular entity was, whether that's a pharmaceutical company, a pharmacist, a GP, maybe a research company, if there was some sort of mechanism whereby people using the platform were rating them…

So, for example, we have things like Amazon and you have customer reviews or Trustpilot and things like that. How would that sit with people?

P32: I don't know if I'd trust it because I know people leave fake reviews online anyway and some people can be really harsh about things when there's no need and people have hidden agendas, so unless it was sort of a peer-reviewed thing, I wouldn't rely on it as much personally.

P34: No, and if you look on…because people do leave reviews via Google et cetera for GPs and if you look at those, there won't be a nice review on there because nobody goes on there to say things that are nice, they just moan about it taking three weeks to get an appointment. So I think that wouldn't necessarily be that useful, a bit like Checkatrade, isn't it, it seems a little bit…I don't know.

P35: I was just going to say I think there's a possibility it could be abused as well for, let's say, a research company wanted some funding for something and they wanted to get a particular contract, you know, there could be some fraudulent aspect to it with fake reviews.

GD: Okay, that's great. I'm wary of the time. I just quickly would like to show you something that we've also developed, how it's maybe different perspectives…some of these you've actually brought up already in conversation, but we're very aware that people might have very different

needs or concerns around using a platform like this, and we want to take a very wide range of stakeholder views into account before we design it, so I'm just going to highlight a couple of views and see what you think, and I'd just like you to maybe choose one perspective that interests you the most, and just tell us what it made you think about.

So we have Ivar, who's a GP, and he's a bit concerned because he's thinking around, well, you can have different activity trackers, and I think as a few of you mentioned earlier, they're not always that reliable and one might say you've run for five kilometres, and the other one might say ten. He actually wants to know whether this kind of data can be trusted or whether there's some kind of…sorry…accreditation process that would give him more confidence about the actual data that patients are giving him.

We've also got Jade, who's a consultant. She's a bit concerned about patients being in complete control of their data because she thinks that if they choose not to share something like their BMI, it might hamper her ability to make decisions in a patient's best interest.

And then we've got Rose. This has been brought up before, she's worried if an app developer sells her data onto other companies without asking for her permission, what can she do about that?

And then May's also wondering, well, if a company say they're going to anonymise the data but then reveal her identity, say, to the government, you know, can they do that without having to ask her?

And then finally we've got Thomas, who's quite happy to share his health data, but he doesn't want the receptionist that lives down the road to see his Fitbit data.

And Chang, who's a health researcher, who thinks it would be a really good thing to have more transparency around what's happening to citizens' health data because at the minute he feels that people don't really know what happens to their health data.

I'm just wondering if any of those perspectives made you have any particular thoughts about the platform, made you think about it in a different way, any concerns?

P31: Accuracy of data sharing, it's like the consultant mentioned not being given the BMI, what if the person doesn't put in an honest BMI? Unless they're measured by their doctor or a nurse, who knows? It would have to have a BMI calculator built into it because not everyone knows how to calculate BMI. People might underplay their symptoms because they don't want to go into hospital and things like that, so there is a real conundrum about accuracy of data input.

P34: I kind of feel like that made it feel like we were getting rid of GPs face to face and that actually everything was going to be done via an app, and that

isn't the case, so the customer, whatever, with the BMI situation, surely that patient would be sat in front of the doctor at some point and BMI would be to some degree obvious. I feel like there's a lot of talk like it's going to take over the world and we won't be able to see a GP again. My perspective of it is in addition to having your normal GP appointments. It's the outcome of those appointments and that information and where it goes after that, and I agree with the whole Fitbit 5K, 10K thing. I think that would have to be on your own…you know, they reckon Fitbit's 88 per cent accurate, it's the most accurate out there. My watch is around about 60 per cent accurate, so if you're going to input your own information, in your own head you've got to understand that it isn't 100 per cent accurate, but it's more information than you'd have if you didn't have a Fitbit.

GD: Okay, thanks, P34. Anyone else? I can't see anybody.

P36: I think there should be sufficient security that the practice secretary, or receptionist, I should say, can't mine into the data that's held for the person. There must be a block somewhere to stop them getting that far.

GD: So you're saying you'd like to have the facility to choose who can actually see this kind of data?

P36: Yes.

GD: Fine, thank you, P36.

[57062_CDIP-FG-06-11-2020-part2]

GD: Same as last time, really. Any initial thoughts, concerns, what you think might be the major sort of benefits and drawbacks of something like this might be?

P34: I think there's some definite benefits for sharing certain data, again going back to my auntie who has Alzheimer's and dementia, currently we have…well, we've not had one at all this year, but every six months at the memory clinic, and that's the only data that they collate to help towards getting to the bottom of Alzheimer's and dementia, and it's nowhere near enough information, a half-an-hour appointment twice a year to discuss how much you've deteriorated or whatever is just nowhere near enough. So for me, being able to share more frequent data certainly with regards to dementia and Alzheimer's would be a great case study.

P33: Yes, I completely agree. I think from the scenario that you gave there, the case study, that person's very much in control of what they were doing with their data and they knew where it was going, they wanted to be part of it, and I think that, yes, research as we all know is just so invaluable, and if you can share your…whether it is just a common thing or it's something quite rare with, you know, and allow other external bodies to access that and do a case study on it, I think, yes, it's really important and something that people should be offered.

P35: Yes, I was just going to say I'd be interested in that side of it, you know? To give that information to research companies or scientists who are researching particular conditions, I really like that idea that, you know, it would give one base, one platform to access to scientists that can access that, rather than relying on recruitment, which I don't know how that works but I would imagine it's a lot more difficult than having a platform where they could access that and, you know, get that information easier.

P32: Yes, because I don't know how people were recruited for this, but I'm on a diabetes group where I've signed up to be part of research and things and that's how I came across this, so if there was an app where you could get that information for different things it would be great and really handy and obviously it benefits us and everybody else, so it would be a great place just if it was in one place? Rather than emails and opting in and stuff and sending back, you could just say, yes, I agree and opt in or something on the phone, it would be great.

P31: So conceptually I think it's a really, really good idea. However, my concern is that if the pharmaceutical companies are able to approach because it's been flagged up this person's got this rare disease, we've really got a limited pool of participants for our research, how many times are they going to be approached? Does it mean that they're going to be under tests the whole time? Is that actually going to be beneficial for them? Is there going to be a marker on the record that they've taken this trial? Would other drug companies be able to see, well, we've done this trial which actually is contraindicatory to what we're doing? Would they be having to rely on that coming up in form-filling or conversation?

So although it's absolutely brilliant, I feel it does put the patient, especially sort of a less-informed patient, it could put them potentially at a risk.

GD: Thanks, P31, that's really important for us to think about, the kind of burden on the patient themselves. So what kind of things do you think would be helpful in terms of having some kind of control and safeguards around this?

P31: I think even if it had to go…again back to the GP who may or may not notice it, but an external advocate who can say, well, look, you've done that one, maybe you shouldn't be doing that one. Because we all know how hard it is to get participants for anything, it's a nightmare, and if you've got a pool of 40 people with a rare disease, a treatment for that would actually be a multi-million pound earning because it could be extrapolated to other diseases as well, that pool of people is going to be, well, we need you, we need you, and there may be a burden of guilt with them to think, well, you know, I need to share my body or my health problem because it will help other people, being very altruistic, but it could be an overburden. So there needs to be some sort of control as to how many trials and what trials you could do. If it's just like a paper-based trial then bring them on, but if it's actually clinical then cause for concern I think.

P36: Now you do one trial and then you just get bombarded with emails to go on other trials that are not even relevant to you.

GD: Sorry, were you trying to say something there, P32?

P32: I was going to say I think the reality is with some trials anyway, you don't really get that information. When I went into hospital initially they thought I might have COVID and I was given information for trials as to…I think there was some kind of trial that Oxford University were running and whether I would want to be part of it, and it was just a three-page A4 document, and because of the way COVID is at the moment, there was no one to discuss it with, there's no family members, you've just got to make a decision there and then, do you want to be part of that trial or not? And it is quite overwhelming and scary to think, right, well, this is the only information I've got here, I think it's about making sure that that person has got as much information and someone they can speak to to discuss exactly what it is they're going ahead with. Because I found that very daunting, thinking, I haven't got a clue about this, it would be nice if there was somewhere you could go to read up some information, even if it was the internet to give you more…

I think it's about how much information you give that person and how you can support them to make an informed decision to go ahead in the first place and knowing at any point you can say, you know what, I've changed my mind about this and what happens if you do do that. So I think it's about the support that you need to put in place and where a person could go if they needed help and who they could speak to.

GD: Thanks, P32, that's really important.

P33: I think on the basis of trials, I think…and we might be going a little bit off track here, by the way…personally I don't think there's enough information where you can access it and see what's available, because even though you might not have a condition that means you want to be part of a trial but you might want to be part of some study, whether it be because of your gender, because of your age, I don't think there's much out there, unless you obviously went out there yourself. I do think that having an app or something where it's actually bringing it to you and saying did you know this study, do you want to be part of it?

Certainly when my mum was alive, I know that she was part of a few sort of…like mammogram or something else, and stuff that she didn't really need to do but she did studies for like five years and was part of these trials to give the data back. She wasn't trialling anything, they were just testing her, you know, just to gather the information, and when she was diagnosed sadly with terminal cancer, she pushed them for a trial drug and said is there any trial drugs that I can take? And actually she got one.

Now we obviously don't know whether she got the actual drug or she got the placebo but she certainly lived a lot longer than what the initial outlook

was. Anyway, as I say, I'm going off track with that, but I think in terms of the trial side, I think if there is an option to have that on your…whether it's on your GP app or whatever it is, where you can access them, I think it's a really good idea.

GD: Thanks, P33:.

P36: GP surgeries tend, in my case, the GP contacts me if there's a particular trial that's relevant to me, and asks me if I'm interested in it. Again, it's gone away from the app because it's a phone call normally. But it is proactive. I'm doing a cholesterol trial now and it's so secretive, they won't even tell me what my cholesterol is when I go. So it just shows how tight-lipped they are at the research base.

GD: So rather than relying on your GP to give you that kind of information, how would you feel about registering yourself and being in control of accessing that kind of information?

P36: Yes, I would quite happily do that as long as it's relevant to the problems I've got. What I don't want is the double-glazing salesmen at the door every two minutes with irrelevant stuff. That's the danger. On a general app, again, unfortunately if it's run by a public company they've got to get some money in and they do tend to diverge from where they initially going, and that's the only bit that bothers me. If it's within the premises of my illnesses, I'm quite happy. I have no problem whatsoever.

GD: Thanks, P36.

P31: Sorry, P35, you go.

P35: I was just going to say I love the idea of being able to sign up so that scientists can ask you to take part in trials, because at the moment, I've looked myself, I've got a [medical detail removed] disability that I had to research myself because the doctors didn't seem to know very much about it at all, and I relied on Google and looking at all these research papers from all over the world, really, just to try and get more information. And I wanted to find trials for that and, you know, I couldn't find any, and you're left with then just sending an email to a consultant saying have you got any trials or anything? So this would appeal to me to sign up, you know, for trials.

P31: Again being the queen of negativity…

GD: We want all…

P31: Sorry…

GD: No, it's really important, go on.

P31: If drug companies or pharmaceutical companies can approach you via the information you provide on your app, if they're the sort of companies that

offer financial incentive which you would not have known about at all as a general member of the public, you may well, if your financial position is poor, feel compelled to have to undertake that thing, and so the only thing I'm going to say is Northwick Park which puts me off any drug trial whatsoever. I'll have a go at most paper-based, but I won't do medication.

So yes, financial incentive offered via this would have to be seriously considered by ethics committees, I think.

P33: I think as well it would be something that you would request it, so it's kind of like you sign up to something and it says are you okay for us to send you emails about this, it would be more of a would you like to take part in a study group or a trial if anything came up under your condition, so you would be able to tick that and then you would…like you say, obviously the money side of it is slightly different but I think if you had control over whether or not you wanted them to contact you, like the scenario you said, rather than people just contacting you just because you're now part of an app and your information is going to be put everywhere.

P34: Yes, and I also think there's a lot of research out there that is happening on people that haven't necessarily got conditions, you know? If you've not got a specific medical condition, you probably wouldn't look at doing research for someone, but they need to gather data on everyone and everybody's fit and healthy, et cetera. So I think being able to sign up and say, yes, that's something I'm interested in, and you put in some of your details, you know, your height, your weight, whatever, blah-blah-blah, and that actually could be interesting to somebody that you'd never even think of because you don't have a condition, you don't look for these research things. So I think that would be a…

P31: Again with the financial ones, I used to be a [Specific role deleted], a lot of my students used to fund their education by doing medical trial after medical trial after medical trial that were all they got paid for and so to be able to access that via this app, oh, I need some money, I'll look for a medical trial that will pay me…worrying.

P32: But couldn't they do that anyway on the internet now? It's a similar thing that you can sign up for that anyway, so possibly if they're going to do that, they're going to do that either way just because they want the money, aren't they, so…

P31: Absolutely, just if it's easier, I've just got to scroll through this app rather than I've actually got to sit down and do a Google search or whatever, or sign up with various companies or whatever, it's just one step easier to do numerous trials, which isn't necessarily wrong, but sometimes you just need to stop and think. I'm sorry I'm so negative.

GD: No, we need…it's not really very helpful if everyone's going yes, that's brilliant. So we want to unpick this and explore everybody's different views and knowing about what concerns people have is hugely helpful because

we can try and plan and design around those, so, you know, it's going to make us think about, well, what safeguards can we put in place, for example? But it does go back to a much deeper ethical question, who does this data belong to and do people have the right to sell their health data if they so wish? But that will digress things massively so I'm not going to go into that. But I would like to just maybe introduce a couple of other perspectives, so the financial one is something that we have had a bit of a think about… Do you want to just bring those up? That's it.

So we've got Pavo who's thinking about, well, how can he be absolutely sure that these organisations are genuine that are asking him for his health data, saying that they're part of a trial? And then Jonas is concerned because he's got three children and he's a bit worried that he's got all the responsibility for managing this data. What if he makes a mistake or shares their data and his children are not happy about that when they're older? And he's also thinking around the GDPR regulation, you know, will that help if some data needs to be corrected, or if his children want their data to be deleted at a later stage?

And then we've got Michelle, so she's saying that actually finding participants is really expensive, takes a lot of time and really slows down the research process, and a platform like this could be really invaluable, especially if people would add specific data about their condition, it might help them to guide their research in a more patient-focused way so that they're researching something that patients are saying that they want and need. And as you mentioned earlier, P31, she's saying the company's quite happy to pay for this data, for example, to make a donation to a charity.

And then we've got Mahmoud who's a lawyer, and he's got some concerns around the consent process, so if someone's consenting to this completely independently and there are no other witnesses like a nurse or a GP, how can we be actually sure that that person is competent enough to give their consent? Or for example, it might even happen when someone's drunk on a night out.

So I wonder if you've got any thoughts around that.

P32: I think with the concern about being drunk on a night out, if it was something like sign up to a study, for me I'd want it to be a two-step process, so you might initially on the app say yes, I'd like to be part of this and then obviously there'll be a telephone call, say, with somebody later who goes through it at a later stage just to make sure that that person's fully aware. I think that's the kind of thing that can help make sure that the person understands what they're doing. So always being able to speak to a person I think helps, so you take that initial step and then somebody comes back to you and has a chat with you, I think that would kind of help some of that. It wouldn't get rid of it completely but at least it would be a bit more of an informed consent.

P31: With Michelle's comment about our company's happy to pay money for the data, I don't want my data sold. I wouldn't be happy with that. Eg to a charity,

well, how would you define the charity? Who would audit that? That's just a minefield. I wouldn't be happy with that at all.

GD: Okay.

P36: Yes, along with P31, I wouldn't take part in any study that had any financial gain in it.

P32: See, I wouldn't mind as long as I knew it was a trusted company that was behind it so I'd want to know that it was somebody's been validated to be on there in the first place, so if they were respected enough, and I could make that decision myself, I'd be quite happy to do that.

GD: That's a really important point, P32, and something we've been considering in a few of the focus groups, and I'm just wondering, I want to go back to everybody's perspectives in a minute, but just if we just stay with that for a moment and think about…so when you say you'd want to know it was a respected company, what kind of decision-making process would you go through to decide whether they're respected or whether you can trust them or not?

P32: I think it's a difficult one, really. I think I'd probably rely on the app itself being trusted and only allowing people on that have been approved. Almost like when you've got an app on an Apple phone and it's gone through a certain rigorous procedure for that person to come on board in the first place, so I'd kind of almost expect them to have to go through a lot to be on there in the first place and then, yes, I'd probably do a little Google search maybe and have a look and see if I could find anything about them. But yes, I'd kind of expect a lot of checks to be done to allow them to be on there in the first place because if they can access some kind of data and contact people then you don't want anybody on there that can contact you, because that's open to huge abuse if that's the case.

P33: If it was safeguarded and they'd only have approved…the NHS or whoever it is that's running this app, would only have approved partners that have access…you're allowing access to, you're not just saying yes, anyone can access my data or do whatever they want with it, and contact me.

P32: Yes, I agree.

P35: I was just going to say, maybe there needs to be layers and types of trials, you know, different levels, I suppose, and different types and to give the person who's giving that information in a simple form what these layers are, you know, all levels, and types of trials that could be available and make it simple to understand.

GD: Are you thinking something around maybe, you know, I consent to sharing a diary, for example, or I want to take part in a clinical trial or sort of a range of different types of participating? Is that what you're saying?

P35: Yes, and I'm aware now with this discussion today that this could be so complicated, it could easily be so complicated with so many types of information but if you maybe could do it in levels of participation or trial, then I think that would be simpler.

GD: Okay.

P35: The main thing, I think, is for people to understand what they're signing up to, you know, to make it simpler, because it could well be a minefield of what people are actually consenting to or signing up to, in my opinion.

P36: When you sign up to these things, looking at it from the research point of view, they need the maximum amount of information, so by withholding a lot of information, it's not helping the research.

P34: Yes, if I can add something into that, so again from my point of view, I would be more than happy to sign up for research for the Alzheimer's and dementia which obviously would be my auntie's answers and so on and so forth, but there'd be absolutely no chance whatsoever that she could participate herself. It would have to be through me, so that's just a point to make is that there'd be quite a lot of people that would be useful for a trial that aren't actually capable or competent of making that decision or giving consent but I'd be more than happy for her to be part of it. But if you asked her, you wouldn't get anywhere with it. So I suppose I'm saying again from a power of attorney point of view or a third party access point of view, how would that work? Because I wouldn't want people contacting my auntie directly.

P33: It comes back to one of the points that was made earlier about how do you know that the person that you're engaging with is capable, is able to make these decisions, because not just…I mean, I obviously know your auntie, [P34], and I know what her condition is, but you've got people who can communicate or are of sound mind but maybe have, I don't know, mental health issues or can't make the decisions on their own, but they do live on their own, so they're vulnerable again. You know, how do you safeguard against people such as that who do generally rely on other people to make big decisions for them, but they can suddenly have access to this and do it all themselves.

GD: Does anyone have any views on how that consent process might be safeguarded or managed?

P32: I think it probably relies on the family member having the app on their phone for that person, really. I don't think people who've got certain health conditions or are elderly, they may not be tech-savvy and they may not have the mental capacity, so I think it would rely on a family member setting it up for them and having it on their phone maybe. I don't quite know how it would work but at least that person would be more in control of the data, and they could know that what was going in and out was correct, really, and that that person's being looked after.

P35: I was just thinking maybe there could be some kind of consent between the…if someone's got Alzheimer's, the relative, and their GP, maybe they could sign up to something together to say we're happy.

P34: Yes, I mean, ideally if power of attorney is registered then the power of attorney would get the same notifications as the actual patient, so it can be monitored, so if my auntie got a notification of a potential trial, it would also come to me on her behalf. So maybe something…having a sort of second signatory for want of a better word.

P32: Yes, I suppose if the app was able to be…say, you have a user's account, a person's account and that person's got a power of attorney, maybe it's one that can be shared between different people so the patient has it on their phone and then a family member also has access to that same information maybe, something along those lines?

GD: What about our comment from the parent? Does anyone have any thoughts around the use of children's data and parents facilitating the sharing of that via this kind of platform?

P32: I don't agree with the deleting information later down the line. You would hope that the parent is sensible enough to look after the children anyway that there wouldn't be that need to worry about the data. I'd be more concerned that the data that's on there is protected and doesn't go anywhere with my child's name on it, that would be my concern over thinking about later down the line, what's on the health records, really.

GD: Anyone else have any thoughts about that?

P33: I think on the child side it just opens a whole can of worms, doesn't it? Because you say you're relying on the parent to be in control of that, but what if the parents are not together and then who's going to be in control of it then? I think it could potentially, yes, open up…and not thought about the child's side of it, we've just been talking about this, but yes, I think that potentially could be a real minefield of issues.

P34: Yes, I would suggest it was an over-18s situation.

GD: A few people nodding there, is that…?

P35: Yes.

P31: Yes.

P32: Yes, I think it depends on what you use the app for, doesn't it? Unless there's a version of the app that's for children, for example, if they're diabetic or something and they use an app to log their blood sugars and stuff, and they're in control of that, that's great that that's there, but I think there's got to be a lot of controls in place to make sure that the parents

know exactly where that data's going and what happens to it, so you'd expect a lot more controls in place for children.

GD: What if someone took part in a trial and then decided not to carry on with it and they wanted to withdraw and a record of them actually taking part in that trial couldn't be deleted because of the nature of blockchain? Maybe something could be put on there to say they've withdrawn from the trial but that record of them actually volunteering to be part of the trial in the first place could never be deleted. How would people feel about that?

P34: I don't know how relevant that would be. I personally wouldn't be bothered. I don't really see any downside to that, unless it stops me getting another trial on the back of it, but then that should be the case, because otherwise you're lying, so I really don't see an issue with that.

P35: I've just thought of something that I would want to be assured that, for example, I'm just using another scenario here, let's say you took part in a demonstration of something and you got on a list or something and then another organisation came along and said, oh, well, you took part in such a demonstration, we'll put you on a black list. So that would worry me if there was some kind of…how can I put it…an organisation taking a view of you because you've left a trial or something. Some negative consequence.

P34: But that would have happened anyway, because it's happened, this is about how it's stored. So unless you were untruthful with the other trial company by saying, no, I haven't been in any other trials, then the data would still be there anyway, whether it's on an app or on a piece of paper, it would be down to the patient to either disclose it or not disclose it, I suppose what we're saying with the app is it's disclosed for you.

P35: I was just meaning that hopefully an organisation couldn't view it as a negative if somebody withdrew from some trial.

P31: I think when you withdraw from a trial, you don't have to give a reason, and it could be you withdrew because you didn't feel the trial was…you know, you weren't happy with the ethics of the trial, even though you'd read the participant information et cetera, or you didn't want to do it anymore. That won't go onto the records so the reasons can be, you know, if you get a black mark for withdrawing, that could be very…I've forgotten the word…confusing or misdirected because you don't know why the person withdrew. Maybe they had a family crisis going on and couldn't continue and that wouldn't be marked with it, and that's just a bit of a difficulty.

P32: I suppose it's like now if you go forward with something and then you change your mind afterwards, it's not supposed to affect your care or anything going forward so I suppose it's not going to affect anything that happens to you in the future, so the doctor potentially to say, oh, well, you've signed up for this trial so we're not going to treat you for this in future. I suppose that's the only concern that it affects your medical care going forward, so it wouldn't take place.

GD: That's interesting, I've not thought of that before. Just very quickly going back to the actual approval process, was it you, P32, I can't remember now who raised that in that you would expect…there's an expectation there that whoever is in control of the platform would have some kind of process. I just wondered if you could expand on that a bit for me, and let me know what kind of thing you were thinking about?

P32: I think it's just about somebody looking into the background and the ethics of the company and making sure that they've got everything in place, so almost like an isotype thing accreditation so there are set things that you must have in place before you can go on it, so it might be you're a registered company and you've not had any, I don't know, complaints against you. So it's just like an audit, really, of that company and I'd expect it to be a standardised process that every company goes through before it can be added to it. So yes, it's a standardised way, I suppose, of just making sure that it's a decent company before they go ahead. Exactly what, I don't know, but I'd expect ethics, whether they've signed up to certain research codes, that kind of thing, really.

GD: And what sort of organisation would you be happy to deal with that on your behalf?

P32: I don't know, really. Obviously not 100 per cent, but I trust the NHS so if it was NHS and it was from the GPs and the doctors, I would think to myself, okay, at least I know it's not privatised. It may not be perfect, but at least I know in general it's for me and the public's health so something like that. Although, I don't know, not that I trust the government, to be fair, but yes, I trust the NHS more than I would, say, Boris Johnson, but… Yes, it's a difficult one, really, I think, but somewhere like the NHS would be a good starting point for me personally.

GD: Thanks, P32. Does anyone else have any ideas around that or particular views?

P33: I mean, surely from the NHS point of view as well, I agree, I think I certainly would trust the NHS, you know, they work with a lot of the pharmaceutical, you know, healthcare and life sciences businesses, so they've already got partnerships with the suppliers, the people who supply drugs and medicines. I think they would perhaps be the first place, I know that there are obviously studies and trials done outside of…you know, your big corporations and a lot smaller charity-funded ones that go on, but I think possibly that would be a way to start engaging with people. I don't know.

GD: Thank you, P33:. Anyone else? What if nobody did that? How would that affect how you might use the platform? So what if it was just entirely open and any pharmaceutical company or app developer could access the platform and it was up to the individual to decide?

P33: I don't think I'd want to share…

P34: No, that would be too open, isn't it?

P36: Wide open to abuse.

P34: Yes, and also as an individual, if you say you can choose who to share it with, what if it's a list of AstraZeneca, GlaxoSmithKline, you might not know who those people are. You might not know who you're saying yes to and who you're saying no to. Granted if it said NHS, I think most people would get an idea on that, but AstraZeneca, for instance, they do a lot of the trials and so on, you know, a lot of people might not have a clue who they are and what they do.

P32: Yes, and then you might get people that sign up that call themselves AstraBeneca and it sounds like it's a bit of a scam, so I'd be worried about, you know, people being misled.

P36: Open to abuse.

P33: Yes, it has to be controlled.

GD: What about if you were given certain information about the companies to enable you to make some kind of risk assessment?

P34: I don't know what information you could give me that would make me think that's okay or not okay.

GD: It's also assuming a reasonable ability of the potential participant to actually risk assess, not everyone can do that.

P32: Unless it was a basic app where you literally put in you wanted to sign up for trials, for example, and you put in your name, address and conditions you'd be interested in sharing information, I don't think many people would share much more information than that. I wouldn't. I certainly wouldn't be putting any medical information in to share with people that I've got no clue about.

P35: I think if it was a platform that would encompass scientists, researchers, drug companies, I think the emphasis would be the trust in the platform itself to have vetted those companies rather than the individual having to think about, you know, are they a legitimate company or person or scientist or whatever.

GD: Right, I think that's really interesting. Victoria, did you want to…?

VN: Yes, I just want to go maybe back to P31's point about the financial incentives, because we had this in different other focus groups already talking through some different versions of how this could work so I just want to lay out a possibility here and want to hear your opinions. So let's say this is an NHS approved app and we know that everybody has been verified

and gone through approval auditing processes. Now there are different possibilities on how financial incentives could work because some of our other focus group participants said they would really like to have some kind of financial incentive, but instead of getting paid a compensation which a lot have in clinical trials, how would you feel about if your data has been incorporated into some kind of trial that then develops into a treatment and a company, let's say, GlaxoSmithKline then developed a drug from that that is then used by the NHS and of course the NHS is paying for this kind of drug.

How would you feel if the people who have been donating the data into this development of the drug also could get some kind of money for that, that has been gained by this company? Would you like it rather in a way that an individual gets back points that they then can choose to either donate to a charity or that they could use for themselves in terms of, like, I would like to access X, Y, Z, additional treatment that is not covered by the normal NHS or would you rule out any kind of financial incentives in this way?

P31: That's interesting. Certainly I would be very averse to people having to pay for a treatment that wasn't available on the NHS because that's the whole idea of the NHS. However, if GlaxoSmithKline are going to make millions out of my data, then I would expect if I was to take part in that drug trial, I would expect some sort of recompense from that. So…yes…it's quite a good question, really, isn't it?

P34: Personally I don't feel very comfortable with the financial gain for this sort of thing. I think it's everybody's responsibility to do research however you want to do it, that's the whole reason we've got the treatment and the drugs we've got now. I don't feel very comfortable with the whole financial recompense.

P32: No, my first thought was maybe when you say they use your data and then the NHS are then paying for these drugs, I'd prefer it if the money went to the NHS. I know at least I've been recompensed in some way, you know, they're not completely making money, at least it would go to the benefit to someone else. It just doesn't quite sit right, does it, that they're going to make all this money, which is obviously what happens anyway, but they're taking your data and then selling it off to the NHS so I'd rather it went into the NHS and helped improve things for everybody.

P34: Yes, that could work well, actually, that as a result of you taking part in this trial. AstraZeneca, GlaxoSmithKline are now donating X amount to the NHS, thanks for your participation.

P32: Or the drug was given to the NHS or something, or widely available, I don't know, at least it would sit better with you than how it…

VN: Instead of donating it to the NHS, how would you feel like donating it, for example, to registered charities? SO, say, well, any kind of data that got in, I could, for example, give to the Alzheimer charity?

P34: Yes, I was just about to say I think a specific charity for whatever, you know, whether it be diabetes, asthma, arthritis, Alzheimer's, a donation to their global research policy would work just as well.

P31: It would be good if the money could go to a charity which then could lead to further research on that specific illness, that would be good.

P35: I was just going to say I think that's an excellent way to improve the participation. If people know that it's going to go to a charity, or a portion or whatever, I think that would improve your levels of participation.

GD: P36, were you trying to say something earlier, sorry?

P36: Yes, I'm going to be really negative here. Yes, personally I prefer the money to go to the NHS, because for me charities have very expensive CEOs and is that money going towards their wages or is it going towards research? That's why I would prefer any money to go to the NHS.

GD: I'm just wondering, there's a range of views around this, how would you feel about being given the choice yourself to decide where that…who is compensated? So maybe if you had a range of options so you could choose to have it yourself, for it to be donated to the NHS or to a nominated charity of your choice? How would you feel if you had that element of control over what happens to…the benefits of you sharing your health data?

P31: I think that would be better, yes.

P36: Fine.

P32: I think especially depending on how much it is, it would be good to have the choice because if it's, say, £5, it might make a difference as to where it's going to go, if it's £200, so at least if you had that choice depending on what it was, you could make an informed choice and think, you know what, this would be more beneficial to go towards this or that.

VN: How would you feel about then if people would use it trying to get the most out of their data in terms of financial value? Of course we have the case that some people might be vulnerable and then would try to make money because they try to make ends meet, but what about the idea that some people who might not be in this vulnerable position would still try to get a financial gain and, for example, collect fitness trackers from 20 people and then go on a run or put this on a dog just to get false data out and then get a financial incentive, this kind of idea that people might cheat the system. How do you feel about that?

P31: I think that's quite a serious concern. But also if you've got the enthusiastic [inaudible 00:43:17], you're going to end up getting your data from a very limited pool [identifying employment data removed] And so that would be

similar thing to happen. You'd just get the same old people and not the breadth of participant that you potentially could need.

VN: So the other effect, not cheating, but the effect of having just a small group who are really enthusiastic and then skewing potential treatments for other people in that way, yes. Good point, thank you.

P36: But how do you police it in the general public? How can you police that?

P34: Yes, I was just going to say I don't think that's got anything to do with the app, again, because I think with or without the app you will have people who will do the research properly and the people who won't do the research properly. Having the app is irrelevant, that's just data gathering.

P31: Then if the app is [inaudible 00:44:28] participating in trials, then whoever's looking to recruit, it's like, oh, they've done five trials, okay, I'll leave them for a little while because although they might fit my criteria, they've done too many trials and I want new data.

P35: I was just going to raise a similar thing that it could be used by the people who are recruiting in a negative way, if they sort of say, oh, well, that person's only took part in trials that have paid them and such a person has only took part, and they've donated to the NHS and such a person's only donated to charity, and they could kind of, I suppose, discriminate against some participants purely because of the way they've, you know, consented to…you know, whether they've been paid or whether they haven't or where it's gone to, you know, some of the funds.

P32: That's where I'd hope that that much information wouldn't be shared though. I wouldn't…

VN: Yes, that's what I was going to say.

P32: They wouldn't share that much in the first place, it should just be there's a trial available that's suitable for you, do you want to take part and it's a two-way thing, it shouldn't be something that's shared anyway.

P35: Yes, so maybe whoever's recruiting perhaps would not have access to that information.

GD: Yes.

End of transcript

**File Name(s)**	57151_FG6-11-11-2020-part1<br>57151_FG6-11-11-2020-part2
**Total Recording Length**	83 minutes
**Number of Participants**	8 (P37-P42) GD/VN/CS
**Final Word Count**	12,253
**Total Number of Pages**	25
**Date Completed**	25 November 2020

[57151_FG6-11-11-2020-part1]

P40: One problem I can see is that the patient themselves may not know what data is best to share.

GD: Yes, that's a good point, P40.

P41: The thing about one of the benefits you said, about if the GP or whoever, whichever healthcare professional looks at the data beforehand, and most appointments are very short and it would maximise the time as you've said. And then also thinking about for any condition, really, if there was a kind of consistent constant thing where the symptoms or whatever health data was being shared, say, if the symptoms were not worsening, they were improving and then the patient might not need to have a clinic appointment as often as possible…sorry, as often as usual. Or the other way around, where they look at the health data being shared and they see that actually symptoms are quite bad at the moment and the next clinic appointment isn't for a year, so let's bring that forward. But obviously that would require a lot of time on behalf of the healthcare professionals but could be a benefit.

GD: Great, thanks, P41. I'd not thought about that, actually. You're the first person to bring that up in a focus group. So yes, that's definitely one of the benefits.

P39: Excuse me, working on [SIMILAR HEALTH APP], one of the big advantages people had, or one of the disadvantages, was getting the app right. So when you work on any kind of app, you've got to start asking appropriate questions and this is where the participation group, people like P41, came in, because they knew what questions should be asked. Which weren't the same as the questions the IT developers thought should be asked, so there's that gap between researchers and your patient participants. Once we'd started to work on that, the big advantage was that the patient group could actually put in their symptoms on a daily basis. This did become a bit intrusive but it was being monitored regularly and in the [SIMILAR HEALTH APP] itself, all the data went straight to the clinicians, so went straight to the consultant who was working on the project.

So when he met next time with the patient, he could see everything, could see how the medication was affecting the patients, from the graphs, and we saw anonymised data, but when patients weren't sure whether medication was affecting them or not, he could actually see it was, so that was a huge advantage.

And following on from what P41 said, because the data was being monitored, that could trigger an earlier appointment or a slightly long timescale. But the interesting thing was within that project, I can never remember data-sharing being discussed. It was more about the advantages of having this than people being aware of what was happening to the data.

The other thing that was quite important was when we couldn't roll it out nationally because it was only [City 1] Royal that had the technology to work with this so again that can be an issue with mismatches in technology.

GD: I wasn't aware that you'd been involved in the [SIMILAR HEALTH APP], so it's good to have that extra bit of insight there. I'm just wondering just very quickly about that kind of data being in the patient's control rather than being directly sent to the GP but the patient holding that data in their phone and having the option about whether to share that or not, and it doesn't have to be [SIMILAR HEALTH APP], it could be Fitbit data or any kind of health application.

P37: P37 here. My concern, I think, has been mentioned which is the patient having control of data, in other words allowing what is released to the clinicians and what isn't, and that makes them, if you like, the clinician themselves in making that decision, whether they should release some information or not, and, you know, will they be qualified to release that information to their GP or consultant that they have this sort of condition or this particular sort of pain or whatever, or keep it quiet? Very difficult, and I would suggest personally I would want my GP or my consultant to know everything, because that way they can make a rounded decision based on all the information that they've got, whereas if I'm giving them cherry-picking the data and giving them bits and pieces, then I'm not sure they're going to be able to get a full picture, and indeed could in fact misdiagnose from the limited amount of information I give them.

The other thing of course is if you lose your mobile phone or whatever device you have, how secure will it be if somebody really wanted to dig into it and your medical information that's on there?

The other thing, last one, I promise, then everyone else can have a go, I mean, I've just come out of hospital, I unfortunately got COVID and was in intensive care, et cetera, I have acquired during that visit in hospital something like five different numbers. So I have a hospital number, I have a COVID number, I have a GP number, I have a consultant number, I have loads and loads of different numbers. How do I know where they should all be? Why on earth can't they all come under my NHS number, for example? That's very unique. I think it is unique.

Well, no, my NHS number is not used, I'm given another hospital number, then a consultant identifier, then a GP number and so forth and so on. It's just very, very difficult to decide, you know, where that data should be stored and they're not necessarily…I said to my GP, can I have the results of my scan, and she said, no, because we didn't order the scan, it was your consultant at hospital ordered the scan, so they have those details. And I'm thinking to myself, this is nonsense, sharing data has to be sort of a little bit more formal and a little bit more organised, so there you go.

GD: Thank you, P37. Some very interesting points said. Did you want to say something, P38?

P38: Yes, so going sort of slightly further back to how the benefits and drawbacks of the [SIMILAR HEALTH APP] app, I think that when it comes to the access rights, sharing the data that you were saying, I think it's imperative that the user, the patient, has that ability to say, actually, no, I don't want to share that data, or I don't want to record that data. I totally get where P37 was coming from, you know, that doctors should know everything, but adding technology to the picture is just about making it more rounded, it's about making it easy, more simple, because there's a lot more people with a lot more things that we need to do. But if I went to my GP now and just for a face to face appointment which I know is very difficult at the moment, but, you know, they wouldn't have the picture of absolutely everything that's going on, they'd only see what I wanted to tell them in person, and I think it should be the same when you're giving them access to your day to day health data.

So, for example, I use Dexcom, it's a continuous glucose monitoring system and I think it's every 13 days, much to my annoyance, it will ask me to share my data if I want to, which is a good thing, because it's continuously reminding me that my data is being shared, but it's giving me the option to stop it, start it, whatever. But with that, I can pick and choose who has access to that, when they have access to that, but, you know, it's something, yes, okay, it's a pain to have to tick it, fine, but I think it is vitally important as well because it's your data, and your data in this scenario is your life, it's your health. It's not just a number, it's not just a piece of paper. It is how you are actually living your life, and if you suddenly don't want to share that with the doctor, that's absolutely fine.

I know especially from someone suffering with a chronic illness, and I'm sure P41 will agree at some point in her life she went a bit off the rails and didn't want to look after herself much at all, and the last thing you want is your doctor to call you up on the Wednesday going why haven't you done this, that and the other? You just want to sort of live your life a bit and then when you're ready to share that information, share it as you wish.

GD: Thanks, P38, that's really helpful to have that perspective from someone that actually monitors their own health with a similar application.

P41: I do agree with you, P38.

P42: Completely. P40's gone down…

GD: Do you want to go ahead, P41? I think he might be able to join us in a minute.

P41: Yes, and it's interesting that, you know, if we look at it from a…I don't know, a best interests perspective, then it's probably best for the healthcare professional to know the ins and outs of what's going on, what your blood sugar's doing or whatever condition, you know, the symptoms and as much data as they can, really, that's the kind of best thing in terms of your health

for them to have a bigger picture. But it should be your choice and your option to consent to allow that, and I think maybe 13 days is a bit much to constantly have that reminder, but it's a weighing up exercise, really, and…what was I going to say?

I've written something down and I don't know what it means. Oh, I was just going to kind of bring in about how in genetics when we get patients through and their referral maybe is sometimes quite limited information, and we need to know, I don't know, some test results or some histology of a cancer or something, as P37 was touching on, you'd think it would be all one system and you can just log in and just there's the NHS number of the patient and all their medical records, and it's not like that, and you have to ask them to sign a consent form and if they don't like that, you can't really get any further, which is frustrating but then I can't… I can't see another way.

I think the majority of people would be happy to just go, oh, yes, I've never had anyone say, no, I'm not signing the consent form, but again it's just about that choice, really.

GD: Thank you, P41.

VN: Maybe I could ask P41 and P38 and open it up to everybody else of course, but if you had these kind of consent forms and notices, what is a good timescale, do you think, of getting those? What do you say, how often should they come up or how little or would there be another way of doing that? Because we offer this automated one, how often would you want to be reminded that you're sharing your data there and do you want to change your settings or do you want to continue that?

P38: So I think personally ideally I'd want it at least every six months that you can actually choose how often you want it to be reminded, because of course healthcare in and of itself is a constantly evolving thing and if suddenly, let's say, not to get into politics too much, but let's say an American-style healthcare system ended up being imposed upon us, I might suddenly say, actually, well, I don't want to share my data anymore, so I need to be able to change things at any given point.

But I think being able to choose maybe a six-month period, a 12-month period, three-month period or like a whenever I choose to actually physically share data, but I do think especially with the Dexcom currently 13 days is rather infuriating.

P41: And I don't use a Dexcom, I use a FreeStyle Libre and up until…when was my last appointment? I think it was August, so up until then, there was a platform called diasend, where the night before my appointment every year basically, the night before I would just plug it in and this magic platform, don't ask me all the technical details of it, would take my data and send it to my consultant. It would be like a snapshot of the last few months and it's not an ongoing thing, it's just that's the data that I've uploaded and it's not

an ongoing thing. And then in August it changed…well, it changed, he said we're generally moving to a different system, and I think it's used by FreeStyle Libre so it's within that, but it's more of a consistent thing where if they were to, I don't know, think I wonder what P41's blood sugars are looking like, then I think they could just log in and see that, so it's a consistent sharing thing, and as I said, that was August.

I went through this rigmarole of logging in and registering and I'm sure there was terms and conditions, but to be really honest, I didn't read them, I would have consented, I've not received any question of whether you want to continue that since then. But I feel fine with that, I feel like if I…it is helpful to have reminders but then I feel like if you've got to a point where you thought I was happy to share and now I'm not, you should be easily able to log in and click a button or whatever without the reminder.

But I'm quite an easy-going, sharing data type of person so…

P39: One of the issues is the data-sharing, the explanations can often be incredibly complex and you often have to read them quite a number of times to actually gain understanding. They can be quite ambiguous too, so you're not sure sometimes whether you're answering yes or no, or whether you should be answering the other way round. I can see you nodding there, P38, and I think this assumes a certain level of education, language skills, you know, especially for people for whom English isn't their first language, for anyone with a learning difficulty or disability, and I just think sometimes not enough thought is put into this. Especially if somebody has somebody working with them, like an amanuensis or a carer, and it's about also who signs the consent form on behalf of someone else.

So it's not as easy sometimes as just clicking a button saying yes or no, and then sometimes you click the button and want to change it and you can't.

P40: Nobody's mentioned anything about IT security yet, which I'm surprised because the NHS has been hacked numerous times. It's not the most secure system in the world, and therefore you're giving that information and it's like putting your money in a bank that leaks money. You wouldn't want to do it normally.

P37: It's P37 again here. I mean, let me give you a personal example which I think are always the best. When I came out of hospital, I was quite unsteady on my feet, and had a fall. My wife had to call the ambulance which came very promptly and they did all medical on me, et cetera, completed a form which apparently is very good, it's completed in real-time and it goes to the ambulance station as the paramedic is completing the form, it actually goes up there and goes on their computer and database and so forth. Very impressed with that.

Having sorted me out and decided there was no reason for me to go to hospital, I said does my GP get a copy of this, and they said no. So I said,

why doesn't my GP get a copy of this? They said we're not allowed to because of data protection, and I said, well, can I give you my permission to send it to my GP, and they said, no, we don't have that facility. If you want your GP to know all about this, you will have a copy of what we've written, you must take it to the surgery and your GP can read it. Total nonsense.

On the form, the paramedic says we will refer you to the falls clinic, so a week later I get a call from somebody at the falls clinic saying, you know, we want to talk to you, et cetera, fine. What I don't understand is why on earth the paramedics can't send the information to the GP but they can send it to the falls clinic? So I've no idea who has access to my GP, why they have access to it, not that I'm one of those guys that's particularly worried. If anybody can help me, I want them to know what is wrong with me so that they can help me. But it just seemed very, very strange and peculiar, the way this data is shuffled around and of course the ambulance data won't go on my big file of medical notes at the hospital.

So there's loads of little databases, or big databases about me, dotted around here and there, with no direct line of access or search. And then another example, I'm on warfarin, I think that saved my life when I went into hospital with the COVID, I said to the pharmacist, I said, have you got a list of things that I can't have if I'm taking my warfarin, and I went…what's that website? He said, I can't tell you, I said, what do you mean? It's for pharmacists only and it's password protected, and I thought, well, how stupid is that? Here I am, a patient on warfarin, right, and there are things you can't take and you must do and mustn't do, and the pharmacist can tell you these things, but he can't give me the website because it's password protected for pharmacists only.

So data is exactly what P40 and the others have been talking about, but it also excludes outside that face to face clinician discussion, and you don't want to keep knocking on your GP's door and saying can you tell me what I can have and what I can't have, et cetera? That sort of information should be readily available. There are sites where it is available but the problem then is you don't know, if you like, how much fake news is in there, how credible are they? If it's got NHS on, I tend to believe it.

So the pharmacist had something which I couldn't have access to, and everything else I could find was, I don't know, American, foreign, whatever, which is really quite silly. Okay, me again, end.

GD: Thank you, P37. It is really helpful to have these individual examples to draw upon, and I just wanted to ask everybody, really, touching on what P37 was saying, so instead of having all this information in lots of different siloes, although the data is not held on the blockchain on the actual platform itself, but you have the ability to be in control of all of your health information and to choose who to share it with and who not to share it with, what kind of choices would you like to have around how you share that data?

P38: I think from a personal perspective, I think it should be open to the patient themselves, so you should have the option of being able to share everything like P37 wants, but also have the option to share very little. At the end of the day, you shouldn't be dictated by this medical machine that, you know, is just data. You should have the choice to go, well, actually, I don't feel comfortable with my data being out there that my doctor, my radiographer can see. What does it matter for them? You should be able to pick and choose very granularly, like [A SIMILAR HEALTH APP] does, similarly to [Dexcom 00:23:13] and other applications do as well. And I think that it's very similar to your data protection within a medical setting anyway, you know? Quite a lot of your information will get told many, many times that whatever we discuss today is confidential, except in certain circumstances, that you're at risk to yourself or others, or something else, I can't remember what it is, something again just as outlandish and ridiculous.

There is always the risk that, as P40 said, that it could get hacked, the NHS isn't the most secure because it isn't a cyber agency, it is a healthcare agency first and foremost, and people need to be aware that that is a possibility, and if their data did get out there, what are the consequences to them personally?

GD: Are you frozen?

VN: Yes, he froze for you as well? So maybe we just…

GD: P42, did you have something you wanted to add? I'll send P38 a little message.

P42: I think that all hospitals should be able to go to one place and pull down the information regarding a patient, say, I came in with a broken leg and a broken hip or something, they shouldn't be able to pull down…say, for example, if I had mental health problems, they shouldn't be able to see that part of things. So I think some parts of it should be restricted.

GD: Okay, I'm just aware of the time. We've got a few extra perspectives we wanted to share with you. We're quite aware that everyone has different needs or concerns around using platforms like this, so before we have a little break, we just want to have a quick look at some of the comments that other people might make around using a platform like this, and I'd like if you can you just to choose one perspective that interests you the most or concerns you the most or tell us something about what it made you think about.

So first of all, we have Ivor who's a GP, and he's a bit concerned about the integrity or validity of data and he says, well, you know, you can have two different kinds of activity trackers and one will say that you've run for five kilometres and the other one will say ten, and he wants to know whether the data that patients are giving him can be trusted, and he's thinking around, well, is there some kind of app accreditation process that would give him a bit more confidence about the information. Thanks for joining us,

P38, sorry, we're just moving on to a couple of perspectives and we're just asking if you wouldn't mind choosing one that particularly resonates with you.

We've got Jade, she's a consultant, and we've brought this up actually in earlier conversation that she's concerned that if a patient decides not to share their BMI with her then that could actually hamper her ability to make a good clinically informed decision.

And then we have Rose, so she's worried about app developers, what happens if they sell her data to other companies without her permission?

And May is thinking around, well, you know, can a company say they're going to identify data and then pass it onto the government without having to ask her?

And then finally the last two, Thomas, he doesn't mind sharing his health data with his GP but he really doesn't want his Fitbit data to be shared with the receptionist who lives down the road from him. And finally Chang, who's a health researcher, can see lots of benefits if there was a bit more transparency about what's happening to citizens' health data because he feels like at the moment people don't really realise.

I just wonder if there's anything in there from those perspectives that particularly struck a chord with anyone?

P37: The consultant that said about the patient not sharing his BMI, I mean, surely that's something that at consultation, you know, the clinician would weigh the patient, take height measurements, et cetera, and be looking at it, so your own calculation of BMI, I mean, I don't…and not necessarily would a patient know their BMI anyway to withhold, so that just seemed a bit watery and weak to me, but there you go, I tend to break scales when I stand on them.

GD: Fair enough. What about if it was something like how much alcohol they drank in a week, for example?

P37: Well, that is very difficult, isn't it, like smoking? You know, is vaping smoking? If you choose to vape, is that classed as smoking, and there are so many different opinions on that. But with alcohol, people say to me, you know, how much alcohol do you drink? I say, well, I might go to the pub today, I haven't been to the pub for weeks, so I might have a couple of pints of beer today but how do you classify that as drinking? And they tend to say not drinking but how many units do you…? And what is a unit? It's all very complicated and it's a very difficult scenario and I think work should be done on making these questions to patients an awful lot more easier that they can respond a lot easier, you know? Like do you drink? Well, yes, of course we do, but, you know, I might have a pint every six months or two pints every six months or whatever, and it's very difficult, isn't it, because the question is, well, could you say how many units of alcohol a day or a week?

Well, one week you might have lots of alcohol and then nothing for six months, so it is difficult.

But anyway, I’m sure you number crunchers will come up with something.

GD: Thanks, P37. Anyone else got anything to say about any of the other perspectives?

P39: Yes, could I say something about the accreditation of apps?

GD: Yes.

P39: I think there does need to be something done about that, or some kind of governance or monitoring system. I had an experience where I ran with my partner and we were running ten kilometres and when we’d finished, I’d run ten kilometres, we both ran together, but he ran one kilometre less, so he had to go off and run around the park again, so that really does make you a bit suspicious because we all assume that what they’re recording is accurate and just one small example like that just does make me question.

So anyone can set up an app and market it, and I’m not sure what accreditation schemes there are, so I think that’s a really valid point.

GD: Thanks, P39.

P41: And just to add a short point, to P37 on a short point and to P39, what P39 just said with the accreditation and how accurate it is and stuff, my Libre patch which is like a little coin on the back of my arm, sometimes when I’m asleep and if I’m lying on it, I don’t know how this works, but it kind of messes up and it looks like on my blood sugar graph that my sugar is very, very low, and it’s actually not, if I tested it. If I tested like a finger prick test or something and compare it, it’s actually not low, it just looks like that from when I’m lying on it.

And so, I’ve had it where I’ve gone to my annual appointment and he said, oh, have you got issues with night-time hypos, and I’ve said, no, it’s from lying on the Libre. So again, accuracy is not proven, really.

And then adding to what P37 was saying, just imagining a situation if you’re a dietitian and you want your patient to keep a food diary, I guess before all this technology it would be an actual diary or it could be on your phone or something, and it’s up to you what you write on that, you could be 40 stone and say I had salad every day for tea, I don’t know why I’m consistently putting on weight. You should have the right to keep a food diary on your phone or whatever and put in what you actually do eat and then still have the option to share that or not, because if it was without the technology, you’d still have the option to say what you really are or what you didn’t or what you really drank or what you didn’t, and I guess it’s just about the clinician using their clinical judgement really and thinking, well, it doesn’t really add up, you know? Everyone has the right to tell porkies, even if it’s

not in their best interests in terms of their treatment and disease progression and things, but it's still a choice.

GD: Thanks, P41. P40, is there anything you'd like to add maybe around IT or…?

P40: I thought the consultant made a good point because if these systems are going to work, then people like her have to have access to this information, and you could argue, I suppose, that the best system would be one where people didn't have any choices, it was all recorded automatically and sent to wherever and if you removed that element of choice, then you get rid of a lot of these troubles.

GD: Interesting.

P39: Can I make another point? I think when we're talking about this, one of my big concerns is access to technology. Certainly, when we were working on [SIMILAR HEALTH APP], a lot of people hadn't used…didn't have a mobile phone, and they never used a keyboard, and so it wasn't just about having…you know, we're talking about digital poverty, really, or just poverty generally like we've got at the moment with remote learning in schools. We're assuming that people first of all have the devices, secondly, you know, that they've got enough money to buy them and keep them up to date, and they have the ability to use them. So in all of these, if you haven't got a device, what do you do? You're excluded.

And the other issue we had, we had to train people how to use them which took quite a lot of time.

GD: Thanks, P39, that's a really pertinent point and something that quite a lot of people have brought up, and it's not unique to this particular kind of idea. The whole digital divide is an issue that we can't get away from, although there may be things that we can do around training and familiarising people with how to use a platform such as this, but thank you for making that point. It is certainly important for us to remember.

Has anyone got anything else they would like to add before we have a very quick break?

P38: There is a quick point. I'm sorry about before, I'm not sure what happened.

GD: That's all right.

P38: I suddenly realised I was talking to frozen people, right, okay, cool. But when it comes to the ability to share the data, the drive to want to share the data, I think it really does need to be in a way that you can, as the patient, pick and choose because, for example, I work with the NHS [specific role deleted]. Whenever people come in to donate blood, we have a very long list of questions that talk quite a lot about your sexual activity, countries that

you visited, you know, things that are highly specific for the purposes that we need.

Now if I was to go and get…if all of my data was shared across…you know, the entirety of my health record could be seen by any clinician that's dealing with me and I, say, broke my ankle next week and I had to go to the radiographer, I don't want my radiographer to see that I may or may not have had sex with somebody who potentially could have given me X, Y or Z. That's not relevant information to them. It needs to have quite a strict hierarchical structure in that, you know, low-level clinicians, like, for example, me, I don't want to see that this person's had a brain tumour in the past, although actually that's a bad example because I probably should know that. But you know, things like that, I don't need to know things about them that I don't need to know, I only need to know what I need. I don't need extra information, whereas some clinicians do.

You should be able to pick and choose that, potentially with a guide that sort of says, you know, best practice would say that you would share it with this person, this person and this person.

GD: Oh, right.

P38: Just blanket sharing I think is a dangerous game because, you know, your radiographer really doesn't need to know that you've been having sex with men from Africa, for example. It's just not relevant at all.

GD: That's great, P38, thanks for bringing that up and again a really interesting and pertinent point. So this is to everybody, not just you, P38, would you want the patient themselves to choose that and then, from what you're saying, then have some sort of alert that says this is sensitive information that could be useful for a, I don't know, a heart consultant or something like that.

P38: Yes, I think a platform that would collect all of your health data so it could be easily shared to anybody who needed it, I think if all that information is there, you shouldn't have the option of just blanket sharing, you should have to say, right, okay, well, my GP they can see everything, because it makes sense for them to know everything, but my dietitian they can only see stuff I've recorded about my diet and my exercise, and my podiatrist can only see things about lower limb injuries or posture, you know, being able to sort of specify different areas, obviously not in that much detail but I think that should be something that is available to people who want to do that. But then the people that don't know technology, don't know how to do it and don't really want to do it, there could be a basic set that is, you know, everything goes to your GP, some things go to this person, and then you can sort of go under guidance.

It is a difficult thing to try and say without not really sitting there with a piece of paper, well, a pad of paper for seven hours and scrolling through…they don't need that, but they could need this but yes…

P39: Can I make another point there, which links in with really what P38's said? You could say that something had to go straight to your GP but then you have a lot of people who work in a GP's practice, so you have receptionists, you have the practice manager and so on, and I think there's an issue around whether it's the actual person themselves, your GP or another partner in that practice and how you limit the information just going to that named person and not to other people who work around that person.

GD: Thanks, P39.

P42: Could there not be something that flags up when somebody's been in it that shouldn't have gone in it? You know, like somebody that's maybe a doctor but is not a doctor of podiatry, like you said, he might be something else, and he's been looking in your records or something like that, maybe something should be flagged up like a little flag and the name of the person.

P38: I was going to suggest that with an audit trail, which would be there anyway because it is sensitive health data, that if somebody did decide to look into your records, you might just get a push notification or an email or something, depending on how the person themselves have actually said, yes, we would like to be notified about that, because I would like to know if randomly, you know, I've not spoken to my doctor in months and randomly they just looked at my data, it would be interesting to see why they've looked at my data. But I think definitely having an audit trail that the patient can see so that you can see whether the receptionist has looked at it because then you'd be asking questions about, well, why?

P41: I think that's a good idea, but it should be that only the people that you've consented to viewing it, like the GP has seen it, and then if there's a situation where, I don't know, you've said no to receptionists or no to a radiographer, which is nothing to do with your sexual health, then that's a bit too late. If you get a notification saying they've seen that, well, it's too late then, they've seen it when you've said that you're not happy with that. So it's all well and good dealing with that situation then but it's kind of past the point then, I think.

P37: I think one of the problems is something that P38 brought up is that you're restricting various, if you like, health professionals to certain segments of your medical records, and I think you quoted the podiatrist that should only have access to lower limb, feet, et cetera. The problem with that is that the podiatrist needs to know whether you're a type one or type two diabetes, because obviously for a type two diabetes, then the podiatrist will normally at a visit take things like pulses and so forth in the feet and temperature readings just to make sure that there is nothing that the diabetes is doing that's affecting the circulation of the feet. It's so difficult to know how you can blank things off and segment it into various files so that only certain clinical professionals could look at it.

I take your point about things like the radiologist doesn't necessarily need to know about sexual health, for example, but there are certain trades within medical clinical practice that do need to know things that you don't consider that they need to know. So I think it's important, and how as patients we can differentiate or how somebody could sort of set various levels, the idea of the audit trail is fabulous, so you can see who's looked at my medical records, it's one of the main, I think, positives.

GD: Okay, I'm aware of the time. If you want to just take a really quick five minute break and come back at half past 12, we'll go through the next scenario then, is that all right? Thank you, everyone, it's been really, really good so far, you've all brought up some really interesting points and I'd like to continue this further, so if you want to switch your videos or your audio off and come back at half past, that would be great, thanks.

[57151_FG6-11-11-2020-part2]

P40: In a lot of research, the participants get the option, up to a certain point, of withdrawing their data for whatever reason, and I'm just wondering why does this have to be permanent? Why can't people withdraw their data if they want to?

GD: That's a good point, P40. So because the data itself is not actually stored within the platform, what's stored on the platform is a record of your transactions with different parties, so it might be a pharmaceutical firm or it might be a researcher at a university, or a GP, for example, on the earlier example. So what it can't do is retract data or change data, but it could add another layer of data which would say, P40 has now withdrawn from this trial on 11 November. How does that sit with you?

P40: Why would you build a system that doesn't allow you to remove data? Why? Because you might find out something about the company that's using it you don't like.

GD: That is one of the benefits and drawbacks of this particular kind of technology is that because it's so difficult to tamper and change, you have this immutable record, this permanent record of what has happened on the platform, but the spin-off of that is that you can never really remove that data but you can introduce more data or another transaction which says this is not accurate, or this person has withdrawn. I don't know, Victoria, if you want to expand on that?

VN: That is correct, so it is very, very hard to actually remove any kind of data, but what you have is so-called amend-only database, so you can then, if you add something like P40 has withdrawn from this study or P40 has ordered the removal of his data from, I don't know, clinic X, Y, Z or trial whatever. So it doesn't remove it but then it gives you the option to see where this access has been done and then you could have this as an audit as well to see if people have actually done what you've requested or if you'd

make an amendment that pops up as this kind of correction of something that has been…

P40: I mean, the research projects I've been involved in, they do have a clear statement which says if you want to withdraw then you can do up to a certain point, obviously once the data's published you can't do anything about that. And if it didn't have that, it would not get through an ethics committee.

VN: Yes, that is true, but again we're not storing any data on that platform. We just store kind of pointers so if you say you would donate data to the…here it's the Liver Trust, and then you'd be, like, yes, I want to stop doing that, or I want to retract that, it would record this transaction that you make.

P40: I understand that. I don't understand what you mean by…the data must be somewhere?

GD: Yes, so, sorry, go on, Victoria.

VN: No, so it's like a platform that sits on top of other data storages, so if, for example, you have the Liver Trust and I don't know, Manchester and Lancaster University sitting there, then they have data storages where different data is being stores for different purposes which we have in our current healthcare system anyway, so that unifies this under one platform, saying that you as a patient are in control of saying, well, I want this data to be donated but only to this organisation and then if you issue this kind of access permission that then allows the actual data storages to share their data.

P40: What's your rationale for not allowing the data to be removed?

GD: I can see what you're saying, P40. The actual research body or the pharmaceutical firm or whoever you're transacting with to say you want to withdraw and remove your data, it is up to them to withdraw it from the study, and that would still happen, but you would also have a record then on the platform you could check and see that they have actually done that. We're not saying that people wouldn't be able to withdraw from a study, for example, they would. You still have all the same rights and it's still the same ethical protocol that research studies have to follow, only with this particular kind of platform organising it, you would also then have the ability to check and see that they have taken you off the trial, for example.

VN: It just makes a record of what you've done, what your decisions have been.

P40: And it would remove your data.

VN: Yes, of course, yes.

P38: The way that I understand that is that obviously I've said that, yes, I would love to take part in this study, and then I've been taken away from the

platform which is directing me to it and it's basically just said that P38 has gone to this other website and is now interacting with them. That's the only information that's kept on the platform, and then I suddenly say actually, no, I don't want to do that anymore because I don't trust them, X, Y, Z has happened. The platform will then say P38 has now withdrawn his consent for this study. So the only two lines of data that you've got is P38 accepting to join the study, P38 no longer accepts to join the study. Is that correct?

GD: Yes. Is anyone else unsure around that?

P39: I don't know.

P37: Yes, I think I'm fairly clear on that. It just raised the question in the back of my mind whilst you were talking, in that I review patient information sheets for pharma, so would the fact that I have reviewed, made amendments to patient information sheets for pharma be recorded in terms of going on a database, so that in ten years' time, a bit like thalidomide and people were being born without arms, et cetera, could it come back and say, ah, but P37 reviewed those sheets, you know? I mean, I'm just throwing that up in the air, I don't know.

GD: That's potentially a function that we could include, and this is why we're having these conversations right at the very beginning before anything is designed to think around what would be the most useful functions and features to have for people, and one of the benefits of this kind of technology is it's very good at providing an audit of particular permissions, for example. So thinking around what you just said, if we wanted to have a smart contract which says this participant, P37, has read the participant information sheet and agrees to all the terms and conditions in that, it could be recorded somewhere.

But the main idea of the platform is that it enables you to interact with other people that might think that your health data is valuable and useful to them and puts you in touch with them. So, for example, here we have Chris, he has openly used the platform to say I'm really interested in liver research and I want to donate my data to anyone that finds it useful. Someone has then contacted him from a pharmaceutical firm and said do you want to take part in our trial, and he's sent a unique code. He then through the CDIP platform will have a record of saying, yes, I am interested, but the transactions that go between him and the firm doing the clinical trial then take place away from the platform.

P39: Right. Would the results from the clinical trial though be given to CDIP?

GD: Potentially, yes, that's something…so again although we can have an audit, we could also have a function where people are advised of the findings, so that in the features when you give your consent you might have an option to say I want to know what happened, what the results of this trial are and set up a function for people to access that information. Especially with clinical trials, there are a lot of different ethical protocols that people have

to follow and this might be one way of ensuring that particular things have been done.

So earlier Victoria was talking about smart contracts as well, that there is a capacity to set something up which is automated so there's no people involved but whereby you say I want to take part in this, this and this, but only under these X, Y, Z conditions and at the end of it, I want to know what happened, or I want to know that my data has been deleted in P40's instance.

P39: I think when you do take part in any kind of research or trials, one of the big issues is you never know what happens. At the end, you very, very rarely get access to academic papers or research or a lay summary or anything saying thank you for what you've done, this is what's happening now, and it doesn't necessarily need to go into your personal detail but I think really when most people take part in anything like this, we all want to know that what we've done makes a difference or has made a difference. You know, I think that's a good way of recruiting people too.

P40: Yes, it's rather like when you give blood now, they tell you where your blood went to.

GD: P41, I'm aware you've got to leave at one, I just want to give you an opportunity to add any comments?

P41: No… I don't really have too many comments on this.

GD: That's fine, just join in as and when you can towards the end. I'm just mindful that you've got to go.

VN: Maybe I can ask, what do you think about if instead of getting you in touch with research organisations there's already approved research organisations in that platform that you can then say you would like to donate to this organisation or to that organisation, but not to that one?

P39: Yes, maybe.

P41: And would you be able to change that over time?

VN: Yes, of course, it's always changeable, yes.

P41: Yes…I'm just thinking…again I always go off on a genetic tangent here, I'm aware, just thinking of situations of ancestry DNA and those databases where mainly in America, really, where people have had this direct to consumer testing and have all this raw amount of genetic data, really, and then been incidents where they've ticked no for sharing with law enforcement and then that's been overruled by a court. So I'm just thinking a bit of a different thing altogether, it's not a murder investigation but I'm just thinking whether that would ever be overturned if you had data that you were sharing and you'd said, oh no, I don't want to share with any big

pharma companies and then in five years' time it was decided that actually for the mutual benefit of the individuals in the country, we're actually going to send all the data off to so-and-so company.

I don't know whether that would ever happen?

VN: It's a good point, yes, thank you.

GD: P42, did you want to say something?

P42: No.

GD: Okay, well, we just want to show you a few other perspectives around this and see what you think. There are some other relevant things that Victoria just picked up on here, especially in terms of pharma interest. I just wonder what you think about these perspectives?

So we've got Pavo who says, how can you be sure that these companies that have access to his data are genuine? Are they who they say they are? And then we have Jonas who's thinking about his children, so he has three children and he's wondering how he's going to manage this data, what if he makes mistakes or shares their data and his children are not happy about that when they're older? Will the General Data Protection Regulation help him if he wants to correct or indeed even delete some data that he's donated on behalf of his children?

We also have P42, so she's a researcher and she says that finding participants is really expensive, it takes a lot of time, something like this kind of platform would really help if they could add very specific data about conditions and help to guide their research in a more patient-focused way so to do the kind of research that patients need and want. And she also says that they'd be happy to pay patients for their data or as an alternative maybe donate something to charity, a related charity, for example.

And then we have Mahmoud who is a lawyer and he's a bit worried about consent, so when somebody consents, if there are no other witnesses like a nurse or a GP, how can we be sure that that person was competent enough to give their consent and they weren't drunk on a night out, for example?

Is there another one, Victoria?

P37: P37 here, can I just comment on the lawyer's comments? Good Clinical Practice, GCP, will state that when a patient signs a consent form, it will have to be explained to that patient, and whoever has explained it, the GP or the research nurse or whoever will co-sign it as a witness. So I think we can discount that one, that he isn't just given a sheet of paper and said sign here. You know, GCP will make sure that that's done properly. I know there's some overlap between GCP, Good Clinical Practice, which is the

Declaration of Helsinki which you all know about in terms of running clinical trials, and what we're talking about here.

GD: What if though you were able to just consent on a platform like this to maybe not a clinical trial but you consent to maybe share basic anonymised data with a university, for example?

P37: Such as what would be the data you're referring to?

GD: Maybe just basic demographic data or lifestyle?

P37: This is something I was going to ask later in the ground discussion was what choices and control would you like to have over your own health data? I'm not sure that health has been defined because you're bringing in your exercise and this and that and the other. Is that health?

GD: Yes.

P37: You say that would be health?

GD: Anything that's to do with somebody's lifestyle, for example, so how they might…so exercise data on a Fitbit application, for example, might be shared either with a healthcare professional or outside with any institution that has an interest in that particular kind of data. We're very exploratory at the moment. There's no defined rules around what kind of data could or couldn't be collected or shared, but potentially that could be included.

P37: Health, if you like, is up for grabs in terms of what the parameters are?

GD: Certainly, for the purpose of this discussion, yes, it's so that we can get as wide a discussion and range of views as possible.

P40: Is there not any apps or Fitbit type things that are given away free on the basis that you allow that data to be uploaded somewhere else?

P41: I think I've seen…is it Vitality adverts on TV where you pay £20 for an Apple watch and they use the health data for insurance purposes, I think, not the NHS but…

P38: Yes, I think that Vitality one is life insurance and you need to have a certain level of activity for you to, one, be able to keep that life insurance and, two, for them not to charge you for the watch, which does sound very dodgy from a legal standpoint but clearly they've managed to jump through lots of hoops there so I don't know, I'm not entirely comfortable with that one. But yes…

P39: There are a lot of consent forms too where you just tick a box, you know, you just check a box on a computer screen so there's nobody monitoring what you do and certainly when I sign up for quite a lot of runs or races, they ask you all kinds of questions about your fitness, or they can do, and

you just tick the box and then you tick I agree at the bottom, but sometimes I do question about what happens to that information. So I think there are issues still around consent forms and how they're done and who is with you when you do them, because we know kids can do all kinds of things too without parental consent.

P41: And that's interesting, I think it was P42 the quote was from about recruiting participants, using this data and maybe a bit of an assumption to make, but I think if you're the type of person to actively input data and share that data, then you're probably more likely to be open to participating in research studies and helping in that sense, so I think that's a good way that they can use this, really, and I will have to go now.

GD: Thank you, P41, it's been great to have you here, thank you.

P41: No, thank you for having me.

GD: If there's anything else you want to add later or any feedback you want to give via email, you know, tomorrow or something, that's fine.

P41: Thank you, thanks everyone.

GD: Nice to have met you, P41, thank you.

P41: All right, enjoy your pork fillet.

GD: Thank you.

P38: Just touching on that, the lawyer again, it comes down to capacity and consent and within sort of healthcare as a general rule of thumb and just in general, actually, I think by law everybody has to be assumed to have capacity until proven otherwise, and it does raise a question in terms of now everything is going online, everything is more digital, who is making that clinical decision whether they do have capacity? I could set up a credit card account in five minutes, but how do they know that I am actually sound of mind to sign up for that credit card? All they're getting is some numbers from credit reference agencies that say, yes, you can give them it or no, you can't. But they're not actually checking my mental faculties, they're not checking anything else like that.

I think as P37 was saying there are standards in place which mean that you have to have it countersigned by a witness but that seems to be sort of fading more and more because people tend to trust what they're reading or they'll just get a massive document of terms and conditions that will be ever changing and they have to agree to them every so often, and it is a difficult one because you do have to treat everyone as if they've got capacity, because unless you're recording them as they do it, you can't say, well, they were clearly sober, they were clearly sound of mind when they did this, they weren't coerced into it, they weren't… You just have to trust it unless there is a way that you can maybe as like a validation process, you know,

you do have to get them just to read on video, you know, they say I consent to the above statements and would like to be involved. That could possibly be a way because then you've got that level of human sort of someone's checking over it.

But then obviously there's always the risk that that human's just going to go, yes, okay, yes, okay, whatever. It is a really difficult one, because you can't say for definite that you can definitely do it one way or another because legally it is a blurry line.

VN: Thanks for that input. It's true, it's really a blurry line, that's why we have all these questions in the focus group as well. I wanted to open up another thing from the stakeholder perspectives, so we had P42, which said they would be willing to pay for the data. How would you feel about if you would get some kind of return? And I want to ask you for two possibilities here, so one this kind of I get some money back, let's say there's a research organisation that develops a treatment and that treatment then will be used in the NHS and normally as it goes these kinds of…for medications or something like that, they would have licences then the NHS pays back to this organisation, and the idea that if you inputted some data then helped develop this treatment that you could get some kind of monetary value back.

And the other option instead of you getting something back that you can choose I want this money to go maybe to the NHS directly or to a certain accredited charity.

P42: I think that's a good idea, to give it to charity, I'd give it to charity probably, and join the research. If they're trying to find a cure for something like rare diseases, it should be looked at properly and I would sign up. If I had a rare disease, I probably would sign up for research.

GD: Would you like the choice, P42, or would you want that as a default option that you couldn't benefit financially individually, but it could be donated to charity?

P42: Yes, I probably would donate it to charity. I wouldn't benefit financially.

GD: Sorry, yes, what I was trying to get at was though that's your personal preference, do you think people should have the option?

P42: Yes, I think they should have the option but that would be my personal preference.

P37: I'm not sure.

GD: Sorry? I think he's frozen. Hello?

P39: Shall I come in while we're waiting…?

P40: It's like, isn't it, where you get paid for giving blood and yet most people think it was a…

VN: Sorry, P40, you will have to repeat that because we were kind of…

P40: Oh, right, the point I was making was is this a bit like in America where you get paid for giving blood and yet most people in this country would think that wasn't right?

P39: Can I support what P40 said? I'm very uncomfortable about payments for this kind of thing. I mean, most people will do it because they want to make a difference, it's altruistic, and I think actual payment, you know, where you've got countries like in India where people are being paid for giving some body parts and so on, and so forth, I just think it creates a whole different climate.

P42: Yes, I agree with that as well.

P38: I think it's not that dissimilar to what the market is currently though. I think what this app would do or this platform would do is just streamline the process and make it easier for people to find paid research opportunities. You know, I'm a member of quite a few different clinical research bodies, mainly because I do want to help, you know, I do want to help them come up with some different kinds of treatment options, you know, or what not. But it is quite nice to also know that there is an element of risk involved and there is sometimes a reasonable financial incentive, shall we say, and I think that's more in place, one, to sweeten the deal, to try and get more people to join it, but also as a way to say, well, you've now given us your…it's a transaction, it's an actual, okay, we're a big pharma company, we're Pfizer, we've got billions and billions of pounds of money that we can spend and we want to spend to create this drug that will make us billions in revenue that we are going to charge the healthcare system billions for.

So I think there should be…you know, if they're going to profit from you and these companies at the end of the day don't care about the individual, they care about the bottom line and the margins that they're going to make, you've got to look at it realistically, they don't care unfortunately, then there should be some transactional value. I think that if there was a platform like this that I would assume would sort of vet the research opportunities, who wouldn't just let every Tom, Dick and Harry go, oh, well, I'm doing a research project about people's fingerprints, or something really weird and stupid, I assume it would all be accredited, licensed and whatnot. Then why not?

But to give people the opportunity to either have it as a personal payment or payment in kind directly to a charity or directly to the NHS or whatever. Me personally whenever I have been paid for a clinical trial, I usually keep 50 per cent and donate 50 per cent just because it's a little incentive for me, but also, I am giving back to charity as well.

GD: Thanks, P38.

P37: I think those of us that do get involved in medical research in administration, if you like, there is a fee attached to that. I mean, I sit on a [health] committee and I get paid as a lay member for sitting on it. And over the years, you've grown up to expect now that you get paid for your involvement, and I don't see anything wrong with that whatever. I think that's perfectly right and reasonable and we're all sat here, little squares on a screen and we're all going to get, I can't remember now, is it £20 in shopping vouchers or something? So we are being paid being here and giving our advice and so forth. I don't see a problem with that. P38 chooses to divvy his up a little bit, but it's up to each of us individually. I might donate my vouchers to a shopping bank, who knows? But that's my personal choice and that is private, if you like. P38's chosen to share with us which I think is very good. Obviously, the pub won't take my vouchers so they'll have to go to a shopping bank or something like that, who knows?

But yes, payment is good, there's nothing wrong with payment and I now feel that any opportunity that comes up without a payment then they're cheapskates, really, because the National Institute of Health Research, the Research Council, all of these funding bodies say, you know, you should pay your PPI, pay your lay people. Anyway, there you go.

GD: Thank you, P37. I think one of the things about this particular platform that P38 brought up is that, yes, I think it's become common practice for any kind of research to thank participants or to give them a reasonable incentive for their time, but if somebody joined a platform like this, how do you feel about the fact that it might attract people that just want the financial incentive. So somebody might then take part in 25 trials because it's very easy. You can do that now, anyone can go online and look for clinical trials or look for research to take part in and make a decision based on how they're compensated for it, but with a platform like this, somebody could potentially just click a couple of buttons and say I'm up for anything, and then go and take part in lots and lots of trials. I'm just playing devil's advocate here, throwing that in there.

P39: I don't think there's much you can do about that. I mean, I'm aware of several people who actually do do that. But if you want public participation and you want real public participation then you're going to have to take whoever comes along. You're nodding there, P38.

P38: Yes, I agree. I think that any data that you can collect from people is still data, whether it's the exact demographic that you want, because people will lie, people will always lie about things to get what they want, but I think these research companies, they've done it for years, they know what they're doing, they know how to screen people out and they know how to say, well, actually, no, you're not what we want. They are very specific. Just because the platform is saying, oh, yes, you can apply for it, it doesn't necessarily mean that you'll get it, it just makes it easier for people to find…you know, and actually probably safer for people to find accredited proper clinical trials

that will pay them. They will get what they want out of it, but also hopefully the company themselves will also get what they want and be able to produce this new treatment, this new medicine, this new technique.

So it's a very difficult marketplace, I think, because everybody has different moral standings, but we're very lucky in the UK that we have free healthcare, whereas in America, P40, you mentioned before that they get paid for donating blood, but at the same time they also have to pay thousands and thousands for that blood transfusion back if they ever needed it, whereas we don't, we get that for free. I think that's the main difference between the US and us in that respect is that we do get it for free, they don't, and so I think, you know, yes, it is a very difficult and there's a lot of little tripwires that you can…

P37: I always want to take issue with people saying we get free healthcare. We don't, we pay a National Insurance contribution of which a sizeable chunk goes into the NHS, huge great chunk, so we don't get it free. We do contribute towards our NHS, so please, people, don't think it's free, it's not. Every week or month, those in employment get a deduction for the National Insurance which goes into the NHS.

GD: Okay, thanks for pointing that out, P37. Just very quickly, we've got four minutes left and there's one thing that you've touched on that's really important for us to explore a bit further and a couple of people have made comments about the verification of these companies, and I just wondered if anyone had any ideas or thoughts around what they would expect if they were using a platform like this, what are people's ideas around who should be allowed to interact in this kind of space and what kind of maybe regulations or processes should be in place before someone is able to use this platform?

P39: I think you're going to have to have some kind of governance system or process that actually monitors companies. There needs to be a formal process through which companies, in my opinion, would have to go before you would want them on the platform, and that is a big concern, especially with so many fake companies, companies moving around, going into liquidation and so on. So there needs to be a formal process.

GD: What would you be looking for, P39? A particular organisation or what about if the people using this platform were able to rate the people that they've transacted with in a similar way to say, for example, on Amazon or TripAdvisor or something like that?

P39: No, I wouldn't agree with that. I think it has to be a governance structure. I mean, I'm a governor in a local college, I'm vice chair of governors and we have a governance process, a protocol and we have instruments and articles, and I think that's the only way you would be able to regulate this because as we know on TripAdvisor people have given all kinds of false information, false returns and so on, and if you haven't got that, how do you get rid of people who are behaving unethically or shouldn't be there at all?

GD: Thanks, P39. Does anyone else have any strong views around that?

P37: Yes, P37 here, I sit on the [name deleted] clinical trials, which is the World [condition removed] Evaluation and one of the things is over the years these trials have been going, we've amassed a phenomenal amount of data, and what we have done with that data is set up a big database and basically clinicians can apply to us and we have a clinical committee and that committee then audits and looks at whoever's asking for this information and whether it fitted, but that's just one…I suppose it's a big database but it's one quite small area within the whole of health and it's only to do with hip fractures. But it's very useful for researchers and things like that, but they have to come to our committee and prove that they are worthy of having…worthy is the wrong word, but they're ethical to be able to have that data. But it is anonymised data anyway so it's not just from that data they can actually find out somebody's name, address, date of birth or anything like that. It's purely their anonymised data.

GD: Thanks, P37. I'm just aware of the time, it is now quarter past one. Has anyone got anything further they'd like to add before we draw to a close for today?

End of transcript
